# Quaternization of Vinyl/Alkynyl Pyridine Enables Ultrafast Cysteine‐Selective Protein Modification and Charge Modulation

**DOI:** 10.1002/anie.201901405

**Published:** 2019-04-09

**Authors:** Maria J. Matos, Claudio D. Navo, Tuuli Hakala, Xhenti Ferhati, Ana Guerreiro, David Hartmann, Barbara Bernardim, Kadi L. Saar, Ismael Compañón, Francisco Corzana, Tuomas P. J. Knowles, Gonzalo Jiménez‐Osés, Gonçalo J. L. Bernardes

**Affiliations:** ^1^ Department of Chemistry University of Cambridge Lensfield Road CB2 1EW Cambridge UK; ^2^ Instituto de Medicina Molecular, Faculdade de Medicina Universidade de Lisboa Avenida Professor Egas Moniz 1649-028 Lisboa Portugal; ^3^ Departamento de Química Universidad de La Rioja Centro de Investigación en Síntesis Química 26006 Logroño Spain; ^4^ CIC bioGUNE Bizkaia Technology Park Building 801A 48170 Derio Spain

**Keywords:** antibody–drug conjugates, bioconjugation, cysteine, microfluidics, protein modification

## Abstract

Quaternized vinyl‐ and alkynyl‐pyridine reagents were shown to react in an ultrafast and selective manner with several cysteine‐tagged proteins at near‐stoichiometric quantities. We have demonstrated that this method can effectively create a homogenous antibody–drug conjugate that features a precise drug‐to‐antibody ratio of 2, which was stable in human plasma and retained its specificity towards Her2+ cells. Finally, the developed warhead introduces a +1 charge to the overall net charge of the protein, which enabled us to show that the electrophoretic mobility of the protein may be tuned through the simple attachment of a quaternized vinyl pyridinium reagent at the cysteine residues. We anticipate the generalized use of quaternized vinyl‐ and alkynyl‐pyridine reagents not only for bioconjugation, but also as warheads for covalent inhibition and as tools to profile cysteine reactivity.

Chemical site‐selective modification offers a means to diversify the function and properties of the protein.^1^ For example, by using the targeting capabilities of an antibody, it is possible to covalently attach a very potent drug to the antibody through a precise chemical reaction to shuttle this drug to a specific tissue.^2^ The toolbox of reactions for protein modification has expanded significantly in the last decade.^3^ Of these, reactions that target proteinogenic amino acids seem particularly suitable to modify native proteins in the test tube. Lysine,^4^ methionine,^5^ tryptophan,^6^ and the N‐7 and the C‐terminus^8^ may now be targeted by using a variety of approaches. However, cysteine^9^ remains perhaps the residue of choice to produce functional and, in particular, clinically useful protein conjugates, namely antibody–drug conjugates (ADCs). This choice is a result of the high nucleophilicity of the sulfhydryl side‐chain combined with the low abundance of free cysteine residues, since many cysteines are paired as structural disulfides. Thus, many research groups have focused on developing efficient methods to chemoselectively modify cysteine‐tagged proteins. For example, electrophiles, such as carbonylacrylic acid reagents for Michael addition,^10^ arylation reactions based on transition metals,^11^ or a selective amino acid sequence,^12^ or conjugate additions at dehydroalanine^13^ formed from cysteine or thiol‐yne reactions using cyclooctynes^14^ have been developed. Each of these methods has relative advantages and disadvantages, but a method based on the simple attachment of a warhead‐like structure whose utility would go beyond bioconjugation and for example be used to tune protein pharmacokinetics, through changes in the overall net charge of a protein, or be used to design cysteine covalent inhibitors is missing from the current toolbox.

Herein, we report the computational chemistry assisted discovery of quaternization of the nitrogen of vinyl‐ and alkynyl pyridines to convert otherwise non‐reactive reagents into ultrafast and chemoselective cysteine‐modifying reagents. The utility of these reagents was demonstrated for bioconjugation and for modulation of electrophoretic mobility through charge incorporation by using microfluidics.

Based on computational predictions, we identified promising alkenes and alkynes through modelling the nucleophilic addition reaction between a simple thiolate or an amine with various electrophiles. As a result of the lower p*K*_a_ of solvent‐exposed thiols relative to amines, it is expected that at neutral or slightly basic pH a larger proportion of thiols would be deprotonated. Activation free energies (Δ*G* ^≠^) for the corresponding addition reactions were predicted by using quantum mechanical calculations (Figure 1 a and b). 2‐Vinyl‐ and 2‐ethynyl pyridines (compounds **1** and **3**) were calculated to be poorly reactive toward thiolates (Δ*G* ^≠^ >24 kcal mol^−1^) and unreactive toward primary amines (Δ*G* ^≠^ >31 kcal mol^−1^). However, when quaternized, the resulting *N*‐methylpyridinium derivatives were calculated to be exceedingly reactive towards nucleophilic addition, with dramatically decreased activation free energies. For instance, 2‐vinyl‐ and 2‐ethynyl‐pyridiniums **2** and **4** were calculated to be more than a billion times more reactive with thiolates (Δ*G* ^≠^=10–12 kcal mol^−1^) than their non‐quaternized analogues **1** and **3**, which shows predicted reactivities similar to those of maleimide^15^ and carbonylacrylic derivatives^10^ previously used for cysteine ligation. In all cases, a complete selectivity for cysteine over lysine addition was predicted, as reflected by the nearly 8 kcal mol^−1^ higher activation barrier calculated for the latter.

**Figure 1:**
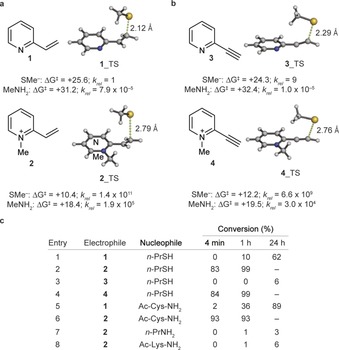
Computer‐assisted evaluation and experimental validation of a) vinyl‐ and b) alkynyl pyridinium electrophile reagents **1**–**4** for cysteine modification. c) Reaction of **1**–**4** with stoichiometric amounts of various nucleophiles.

We validated our computational predictions by reacting stoichiometric amounts of minimal cysteine and lysine side‐chain model compounds (that is 1‐propanethiol and 1‐propylamine, respectively) with commercially available **1**–**3** and synthesized reagent **4** (Figure 1 c), and monitoring reaction with ^1^H NMR spectroscopy (3 mm, phosphate buffer in D_2_O, pH 7.6; see the Supporting Information for additional data and a detailed discussion on the effect of using a slightly more acidic pH). In agreement with theoretical predictions, the reactions with non‐quaternized reagents **1** and **3** were very slow and gave 62 and 6 % conversions, respectively, of addition products after 24 hours (Figure 1 c, Entries 1 and 3). On the contrary, pyridine *N*‐methylated reagents **2** and **4** gave a conversion of 99 % of addition product after 1 hour with only 1 equivalent of electrophile (Figure 1 c, Entries 2 and 4). 2‐Alkynyl reagent **4** gave a 6:4 mixture of isomers as products, namely the *cis* and *trans* alkene adducts (See the Supporting Information for their structural characterization and thermal stability). The kinetic profiles obtained with a protected cysteine (*N*‐acetylcysteine amide, Ac‐Cys‐NH_2_) were similar, albeit slightly faster than with 1‐propanethiol (89 % adduct formation in 24 hours with non‐quaternized **1**, and 93 % adduct formation in <4 min with quaternized **2**; Figure 1 c, Entries 5 and 6). Also, in agreement with computational predictions, the fast kinetics observed with quaternized pyridinium reagents **2** and **4** were comparable to those of *N*‐ethylmaleimide,^15^ for which the reaction with 1‐propanethiol and Ac‐Cys‐NH_2_ were complete within the 4 min necessary to obtain the first ^1^H NMR spectroscopic measurements, although the formation of an undetermined alkene by‐product was observed in these reactions with maleimide (see the Supporting Information). With lysine side‐chain mimic 1‐propylamine (Figure 1 c, Entry 7) and *N*‐acetyllysine amide (Ac‐Lys‐NH_2_; Figure 1 c, Entry 8) the reactions with quaternized 1‐methyl‐2‐vinylpyridinium (**2**) were exceedingly slow (<6 % adduct formation after 24 hours with 1 equivalent of electrophile). This result agrees with the computationally predicted orthogonality of these electrophiles towards cysteines, with no expected reaction on typically solvent‐exposed lysines. It is interesting to note that the terminal alkyne hydrogen in compounds **3** and **4** quickly exchanged to deuterium in D_2_O at pH 7.6, particularly in the case of quaternized **4**, for which deuteration takes place within 4 min. H/D exchange at the vinyl hydrogens of quaternized **2** was also observed when [D_7_]DMF was used as a co‐solvent.

Accurate reaction rate constants could not be obtained for very reactive quaternized pyridiniums **2** and **4**, and *N*‐ethylmaleimide as a result of the ultrafast kinetics observed under the reaction conditions used (3 mm was the detection limit for ^1^H NMR spectroscopy in our experiments). Also, the high level of sensitivity to small reagent concentration changes on the observed rate for second‐order reactions, further complicates the derivation of the rate constants under stoichiometric conditions. Furthermore, although alkynyl pyridinium **4** was selective towards mono‐functionalization under strict stoichiometric conditions, it was found to undergo double thiol addition to the external position of the triple bond to give a stable dithioacetal when a slight excess of the thiol was used (see the Supporting Information). Once the dramatic effect of pyridine quaternization in boosting the electrophilicity of reagents **1** and **3** was demonstrated and given the very similar and ultrafast reactivity of quaternized reagents **2** and **4** towards thiols, we decided to use more convenient 1‐methyl‐2‐vinylpyridinium **2** for cysteine‐selective modification on proteins (to avoid terminal alkyne deuteration and formation of mixtures of potentially reactive alkene isomers upon nucleophilic addition).

Having demonstrated the superior reactivity of **2** towards thiols on small molecule models, we decided to test the use of vinyl pyridinium reagents towards cysteine‐tagged proteins (Figure 2 a). We selected four representative proteins, the C2A domain of Synaptotagmin‐I, Annexin‐V, ubiquitin, and albumin, which display either natural or engineered single surface‐exposed cysteines (see the Supporting Information). In all cases, and with stoichiometric amounts or only a small excess of **2** (1 equiv for ubiquitin and albumin and 10 equiv for annexin‐V and C2Am), under buffered (NaP_i_ pH 8.0, 50 mm) conditions for 1 hour at 37 °C, complete conversion into the corresponding thioether adduct was observed, as confirmed by LC‐MS analysis, and in high yield (>95 %), as shown by Bradford protein assay (Figure 2 b‐2e; see also the Supporting Information; no reaction was detected with either **1** or **2** under slightly acidic pH 5.5). Despite the use of 10 equiv to ensure efficient modification of even sterically crowded, and thus poorly deprotonated, cysteine residues, such as in the case of Annexin‐V, no non‐specific reactions at lysine were detected. Other studies that use vinyl‐substituted pyridine derivatives achieved a maximum of only 85 % conversion after 24 hours,^16^ which demonstrates well the effect of quaternization of the pyridine on the kinetics of the bioconjugation reaction. Furthermore, the final conjugates did not react with Ellman's reagent [5,5′‐dithiobis(2‐nitrobenzoic acid)] that showed that all cysteine had been consumed (see the Supporting Information). Importantly, we verified that the formed thioether bond is stable towards potential thiol exchange reactions, and the conjugates were also fully stable in human plasma in buffered pH 5–8 solutions (see the Supporting Information). Next, we confirmed that the conditions used are mild and do not induce changes in both the structure or function of the native proteins. In the case of albumin, conjugate albumin‐**2** showed no significant changes in its secondary structural content as determined by circular dichroism (CD; Figure 2 f) and retained its ability to bind to the neonatal Fc receptor (FcRn) as determined by Surface Plasma Resonance (*K*_D_=11.8 μm for albumin versus *K*_D_=8.88 μm for albumin‐**2**; Figure 2 g). Together, our data demonstrates the generalization of a method for cysteine‐selective protein modification based simply on the use of quaternized vinyl pyridinium reagents. The products are formed in high conversion and yield, are stable in human plasma, and both native structure and function activities are retained.

**Figure 2:**
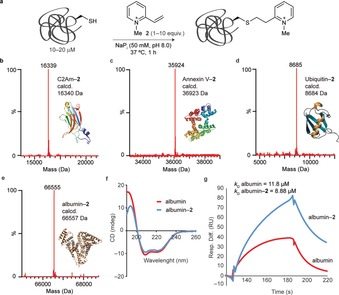
Cysteine‐selective protein modification. a) The reaction of a cysteine‐tagged protein with **2**. General conditions: a cysteine‐tagged protein (10–20 μm) is reacted with **2** (1–10 equiv) in NaP_i_ (50 mm, pH 8.0) at 37 °C for 1 hour. b–e) ES‐MS spectra of the product of the reaction between b) C2Am, c) Annexin V, d) Ubiquitin, and e) albumin with **2** (deconvoluted spectra; expected increase of 118 Da in the total protein mass). f) Comparative CD analysis of albumin and albumin‐**2**. g) Surface plasma resonance of binding to the FcRn receptor. *k*_D_ for rHSA and rHSA‐**2**.

Next, we decided to demonstrate that vinyl pyridinium reagents may be easily functionalized first through quaternization of the pyridine nitrogen with an alkyne linker followed by subsequent derivatization through Cu^I^‐catalyzed azide–alkyne cycloaddition reaction with a suitable synthetic motif that displays an azido group (see the Supporting Information). We chose to build a quaternized vinyl pyridinium reagent that features cytotoxic drug monomethyl auristatin E (MMAE; Figure 3 a). By connecting the cysteine‐selective reagent and the drug, we introduced the dipeptide valine–citrulline (ValCit), which is known to cleave on exposure to cathepsin B,^17^ and the self‐immolating spacer *p*‐aminobenzyl carbamate (PABC).^18^ This reagent **5** was then conjugated to Thiomab, an HER2 targeting antibody that has been engineered to contain an additional cysteine residue at position 205 in each light‐chain (Thiomab LC‐V205C).^19^ To our delight, the conjugation reaction proceeded to completion with 5 equiv per light‐chain of **5**, within 1 hour at 37 °C in NaP_i_ pH 8.0, 20 mm buffer (Figure 3 a). LC‐MS analysis showed a single modification in each‐light chain and no modifications in the heavy‐chain (Figure 3 b) to form a conjugate with a precise drug‐to‐antibody ratio of 2. Importantly, Thiomab‐**5** conjugate retained its specificity towards Her2 positive cells, as demonstrated by flow cytometry analysis (Figure 3 c and d). Our data demonstrate the use of quaternized vinyl pyridinium reagents to efficiently generate homogenous and functional antibody conjugates.

**Figure 3:**
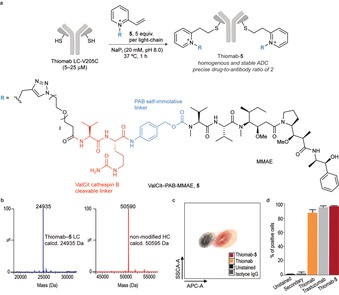
Cysteine‐selective antibody modification. a) Schematic for the bioconjugation of **5** to Thiomab and chemical structure of **5**. b) ES‐MS spectra of the light‐ and heavy‐chain of Thiomab after conjugation with **5**. c) Counter plot indicates the binding affinity of Thiomab‐**5** to Her2 expressing SKBR3 cells at 50 nm. d) Percentage of SKBR3 cells bound to Thiomab‐**5** at 50 nm. Trastuzumab and Thiomab were used as positive controls and IgG Isotype was used as negative control.

The conjugation of a protein can be monitored directly by following changes in its electrophoretic mobility because the quaternized vinyl pyridinium reagent introduces an additional charge of +1 per cysteine residue to the net charge. For instance, albumin features one single free cysteine and thus conjugation of **2** adds +1 charge to the overall net charge of the protein (charge ca. −7 at pH 7). We decided to test this hypothesis by using a microfluidic free‐flow electrophoresis device,^20^ which allows the charge of proteins to be determined under native conditions in solution and requires only low volumes of sample. The samples were introduced into the device with a flanking buffer, and the deflection of the protein samples was observed while a transverse electric field was applied by increasing the potential between electrodes in the device from 0–120 V (Figure 4 a). When the measured deflection in the field was converted into drift velocity (*V*_drift_) and the electric field inside the devices was calibrated (Figure 4 b), a clear difference in the electrophoretic behavior of albumin and albumin‐**2** was observed. Furthermore, the electrophoretic mobilities were estimated to be: −1.71±0.21×10^−8^ m^2^ V^−1^ s^−1^ to give a charge of about ≈−6.84±0.58 and −1.12±0.10×10^−8^ m^2^ V^−1^ s^−1^ to give a charge of about −4.64±0.12 for albumin and albumin‐**2**, respectively. The decrease in mobility can be explained by the direct proportionality of the charge (*q*) to the electrophoretic mobility [*μ*_el_; *q*=(*μ*_el_×*k*_B_ *T*)/*D*], which implies that if the charge is decreased, the mobility should also decrease as confirmed by the data. Moreover, because we are adding positive charge through conjugation of the quaternized vinyl pyridinium reagent **2** into a negatively charged albumin, the net charge of conjugate albumin‐**2** should be smaller, as observed in the results obtained with the microfluidic electrophoresis. These data thus demonstrate the application of site‐selective protein modification to change the overall net charge of a protein in a controlled manner, and consequently modulate its electrophoretic mobility.

**Figure 4:**
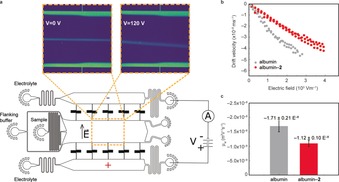
a) Representation and b),c) results from microfluidic determination of the electrophoretic mobilities of native albumin and albumin‐**2**. The device is operated by withdrawing the sample and flanking buffer from the outlets to create a laminar stream of sample that flows between two electrolyte streams, separated by the buffer. When a potential difference is applied between the electrolyte streams, a transverse electric field is created in the separation chamber and the deflection of the sample can be monitored by using the intrinsic fluorescence of the protein at 280 nm.^21^ Both samples have a unique response to the electric field and this can be seen by plotting electric field (*E*) versus the drift velocity (*V*_drift_) b), which is used to determine the representative mobilities (c).

In summary, an efficient and irreversible cysteine‐selective bioconjugation method is reported. This method is enabled by the discovery that quaternization of the nitrogen of vinyl‐ and alkynyl pyridines transforms these molecules into extremely reactive electrophiles towards thiols. We demonstrate the utility of these reagents for cysteine‐selective bioconjugation of five different protein scaffolds, including a clinically used antibody. Importantly, the conjugates formed are resistant towards thiol exchange reactions and retain their native activity. By using a microfluidic setup, we show that the electrophoretic mobility of a protein may be modulated through simple attachment of the quaternized vinyl pyridinium reagent at cysteine to introduce a +1 charge to the overall net charge of the protein. This property of the quaternized vinyl pyridinium may prove useful to enhance the hydrophilicity, half‐life in circulation, or internalization rates of ADCs. The simplicity of these reagents combined with their excellent reactivity and selectivity towards thiols, makes them ideal for protein bioconjugation, but also for use as warheads in the design of cysteine covalent ligands^22^ or to probe hyper‐reactive cysteines within the human proteome in chemical proteomic approaches.^23^

## Conflict of interest

The authors declare no conflict of interest.

## Supporting information

As a service to our authors and readers, this journal provides supporting information supplied by the authors. Such materials are peer reviewed and may be re‐organized for online delivery, but are not copy‐edited or typeset. Technical support issues arising from supporting information (other than missing files) should be addressed to the authors.

Supplementary
