# Supplementary material for: Quaternization of Vinyl/Alkynyl Pyridine Enables Ultrafast Cysteine‐Selective Protein Modification and Charge Modulation
Source: Angew Chem Int Ed Engl. 2019 Apr 9;58(20):6640–4. doi: 10.1002/anie.201901405 (PMC6618083; doi:10.1002/anie.201901405)
Supplement: Supplementary file 1 — Supplementary [file ANIE-58-6640-s001.pdf]

Angewandte Chemie
Eine Zeitschrift der Gesellschaft Deutscher Chemiker

Supporting Information

Quaternization of Vinyl/Alkynyl Pyridine Enables Ultrafast Cysteine-Selective Protein Modification and Charge Modulation

Maria J. Matos[+], Claudio D. Navo[+], Tuuli Hakala[+], Xhenti Ferhati[+], Ana Guerreiro, David Hartmann, Barbara Bernardim, Kadi L. Saar, Ismael Compañón, Francisco Corzana, Tuomas P. J. Knowles,* Gonzalo Jiménez-Osés,* and Gonçalo J. L. Bernardes**

anie_201901405_sm_miscellaneous_information.pdf

Supporting Information

Table of Contents

1. Reaction Kinetics S2
2. The effect of pH on reactivity S16
3. Quantum Mechanical Calculations S18
4. Synthesis S25
5. General Procedures for Protein Modification and Characterization S32
6. Ubiquitin-K63C Modification and Characterization S33
7. Annexin V-315C Modification and Characterization S36
8. C2Am-Cys95 Modification and Characterization S39
9. rHSA-Cys34 Modification and Characterization S52
10. Thiomab-LC-C205C Modification and Characterization S60
11. Analysis of Secondary Structural Content by CD S66
12. Determination of FcRn Binding by Surface Plasmon Resonance (SPR) S68
13. Cell Specificity Analysis by Flow Cytometry S69
14. Microfluidic Determination of Electrophoretic Mobility S70
15. NMR Spectra S73
16. References S77

1. Reaction Kinetics

pH 7.6

The second-order reaction constants of the reactions of electrophiles **1-4** and *N*-ethylmaleimide (**NEM**) with small-molecule models 1-propanethiol (**PrSH**), *n*-propylamine (**PrNH**), *N*-acetylcysteine amide (**Ac-Cys-NH$_2$**) and *N*α-acetyllysine amide (**Ac-Lys-NH$_2$**) were determined by ^{1}H NMR (400 MHz) at 298 K in sodium phosphate buffer in D_2O (pH 7.6, 100 mM). Electrophile concentration was 3.0 mM in all cases. A ^{1}H NMR spectrum was recorded every 85 s (number of scans: 16). Around 5 min were needed to record the first spectrum after mixing the reagents. The observed second-order rate constants k_{obs} (i.e. k_2) were derived from the slope of a linearly-fitted plot of the inverse of the electrophile concentration (1/[E]) *versus* time.

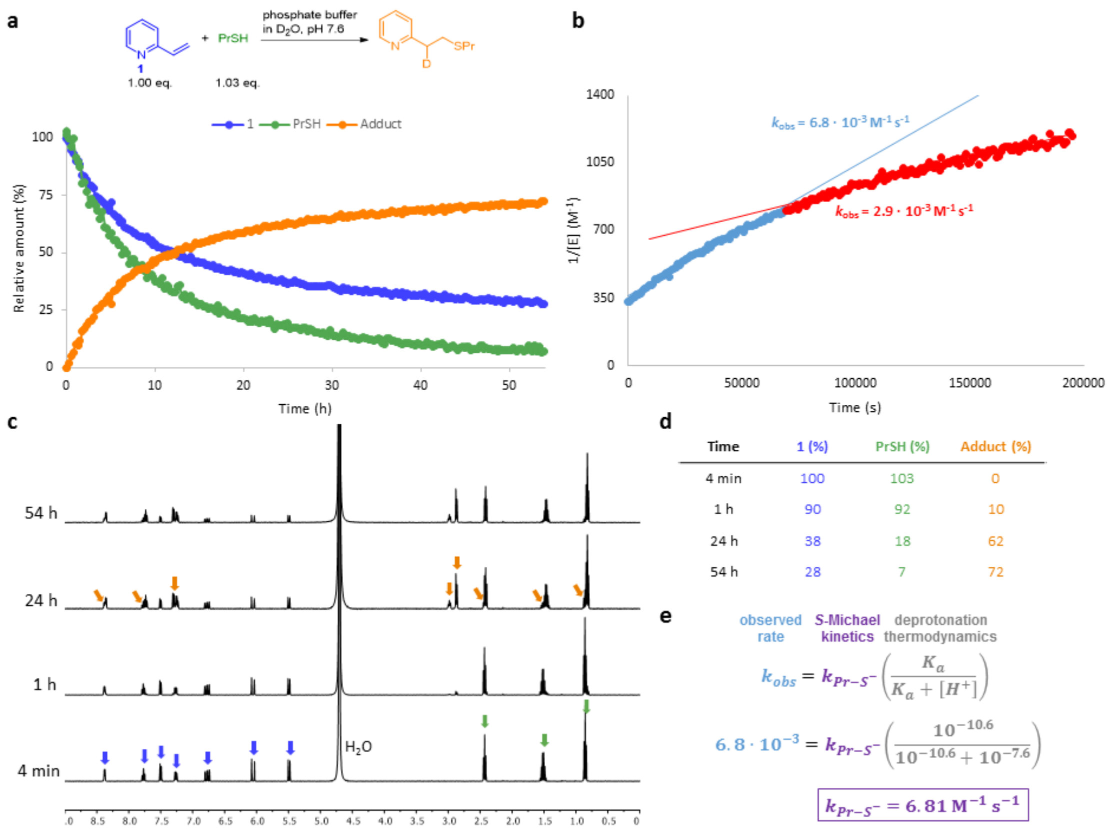

Figure S1. a) Monitoring of reaction between **1** (blue) and **PrSH** (green) in sodium phosphate buffer in D_2O (pH 7.6, 100 mM) by ^{1}H NMR (400 MHz) at 298 K. The corresponding adduct is shown in orange. b) Estimation of the second-order reaction constant (k_{obs}) using the linearly-fitted regions of the 1/[**1**]) *versus* time plot. c) Overlay of ^{1}H NMR spectra at different reaction times. Blue, green and orange arrows point to signals of compounds **1**, **PrSH** and the corresponding adduct, respectively. d) Relative ratio (%) of compounds **1**, **PrSH** and the reaction adduct at different reaction times. e) Estimation of the intrinsic nucleophilic rate constant (k_{Pr-S^-}) from the observed kinetic rate constant derived from the initial measurements (k_{obs}) (in blue), thiol acidity constant (K_a) and buffer acidity ($[H^+]$).

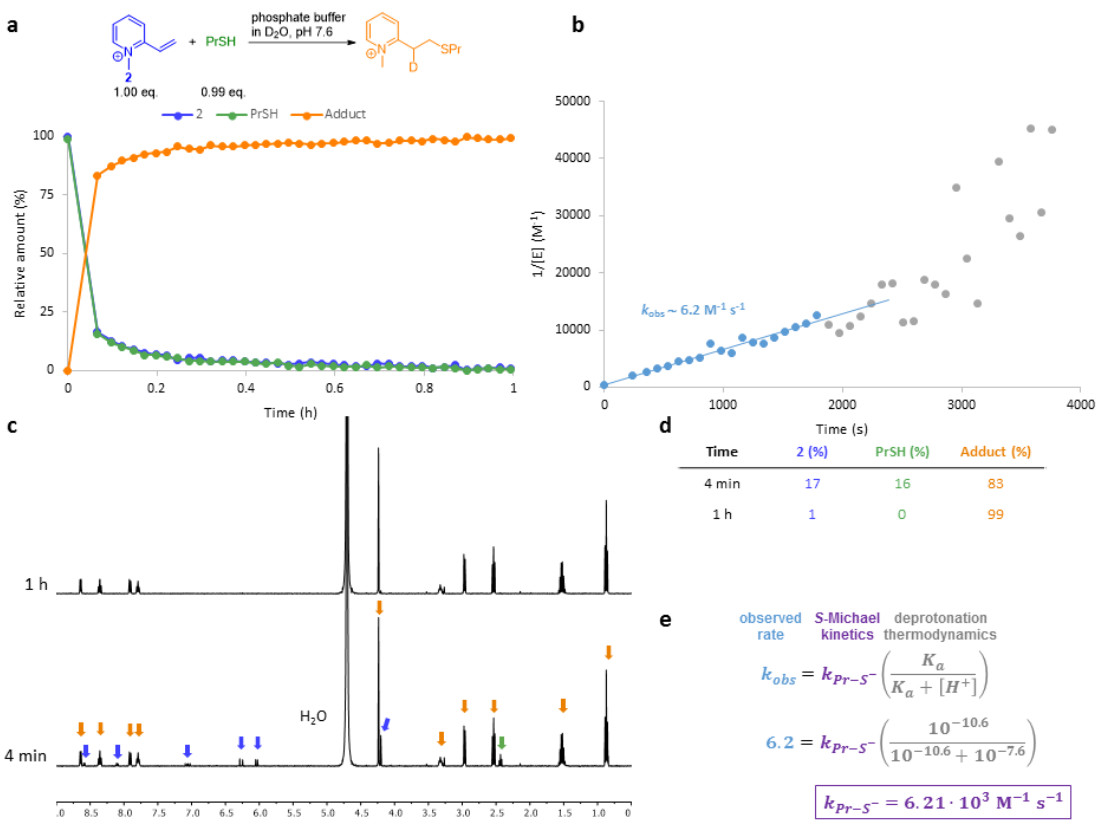

Figure S2. a) Monitoring of reaction between **2** (blue) and small-molecule model **PrSH** (green) in sodium phosphate buffer in D_2O (pH 7.6, 100 mM) by ^1H NMR (400 MHz) at 298 K. The corresponding adduct is shown in orange. b) Estimation of the second-order reaction constant (k_{obs}) using the linearly-fitted region of the 1/[**2**]) *versus* time plot. c) Overlay of ^1H NMR spectra at different reaction times. Blue, green and orange arrows point to signals of compounds **2**, **PrSH** and the corresponding adduct, respectively. d) Relative ratio (%) of compounds **2**, **PrSH** and the reaction adduct at different reaction times. e) Estimation of the intrinsic nucleophilic rate constant (k_{Pr-S^-}) from the observed kinetic rate constant derived from the initial measurements (k_{obs}) (in blue), thiol acidity constant (K_a) and buffer acidity ($[H^+]$).

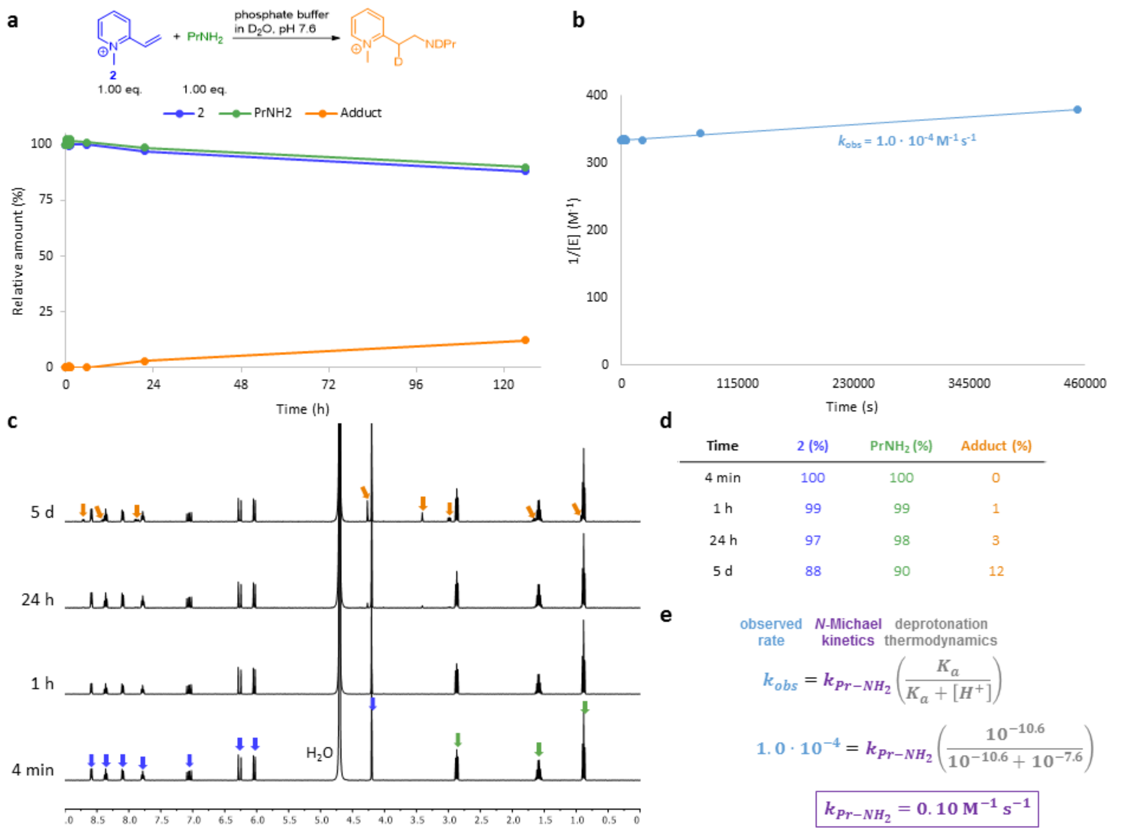

Figure S3. a) Monitoring of reaction between **2** (blue) and small-molecule model **PrNH₂** (green) in sodium phosphate buffer in D_2O (pH 7.6, 100 mM) by ^1H NMR (400 MHz) at 298 K. The corresponding adduct is shown in orange. b) Estimation of the second-order reaction constant (k_{obs}) using the linearly-fitted region of the 1/[**2**]) *versus* time plot. c) Overlay of ^1H NMR spectra at different reaction times. Blue, green and orange arrows point to signals of compounds **2**, **PrNH₂** and the corresponding adduct, respectively. d) Relative ratio (%) of compounds **2**, **PrNH₂** and the reaction adduct at different reaction times. e) Estimation of the intrinsic nucleophilic rate constant (k_{Pr-NH_2}) from the observed kinetic rate constant (k_{obs}) (in blue), ammonium acidity constant (K_a) and buffer acidity ($[H^+]$).

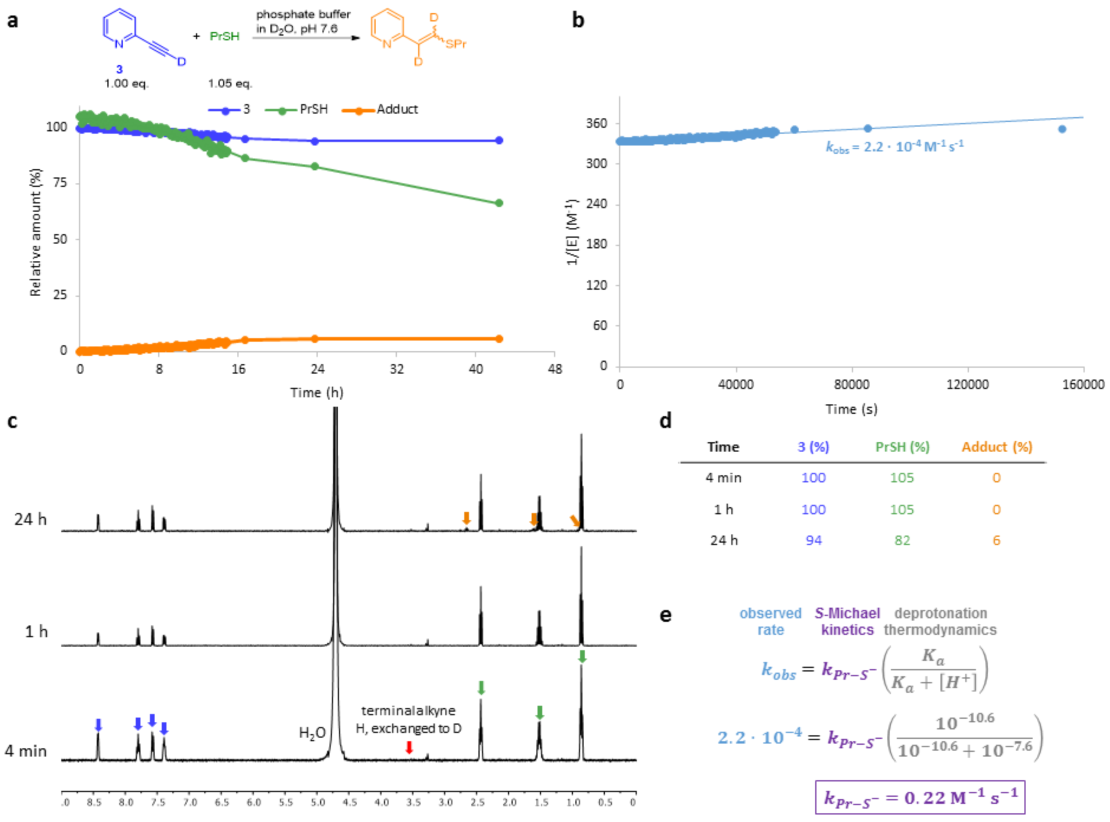

Figure S4. a) Monitoring of reaction between **3** (blue) and small-molecule model **PrSH** (green) in sodium phosphate buffer in D_2O (pH 7.6, 100 mM) by ^1H NMR (400 MHz) at 298 K. The corresponding adduct is shown in orange. b) Estimation of the second-order reaction constant (k_{obs}) using the linearly-fitted region of the 1/[**3**]) *versus* time plot. c) Overlay of ^1H NMR spectra at different reaction times. Blue, green and orange arrows point to signals of compounds **3**, **PrSH** and the corresponding adduct, respectively. d) Relative ratio (%) of compounds **3**, **PrSH** and the reaction adduct at different reaction times. e) Estimation of the intrinsic nucleophilic rate constant (k_{Pr-S^-}) from the observed kinetic rate constant (k_{obs}) (in blue), thiol acidity constant (K_a) and buffer acidity ($[H^+]$).

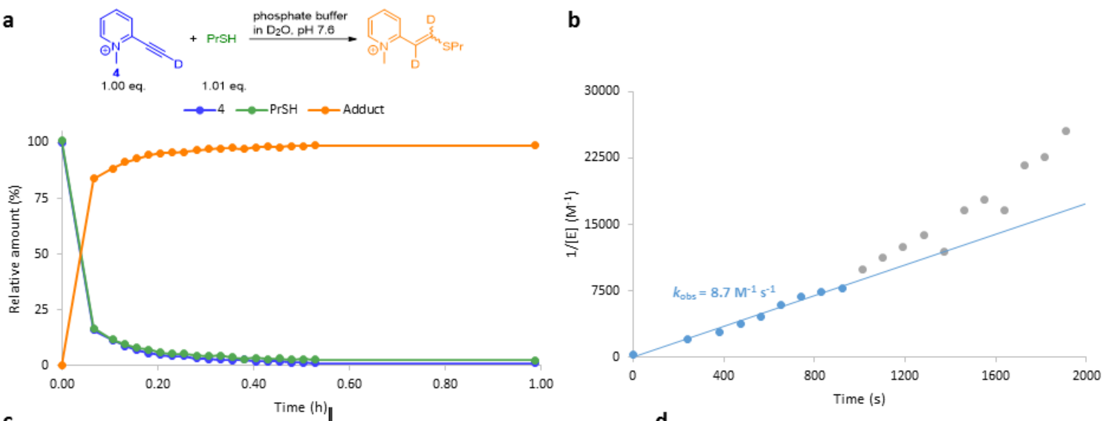

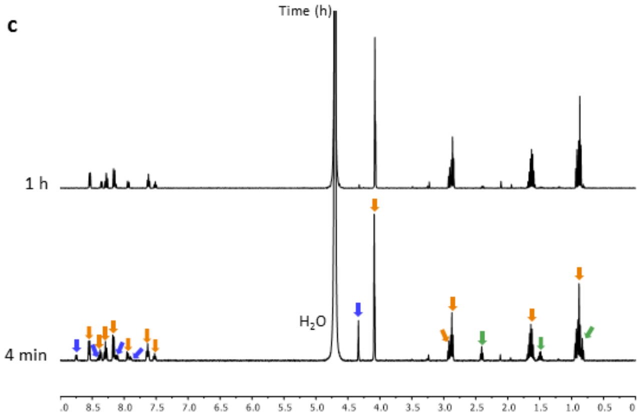

d

Time	4 (%)	PrSH (%)	Adduct (%)
4 min	16	17	84
1 h	1	2	99

e

observed rate — S-Michael kinetics — deprotonation thermodynamics

$$k_{obs} = k_{Pr-S^-}\left(\frac{K_a}{K_a + [H^+]}\right)$$

$$8.7 = k_{Pr-S^-}\left(\frac{10^{-10.6}}{10^{-10.6} + 10^{-7.6}}\right)$$

$$k_{Pr-S^-} = 8.71 \cdot 10^3\ \mathrm{M^{-1}\ s^{-1}}$$

Figure S5. a) Monitoring of reaction between **4** (blue) and small-molecule model **PrSH** (green) in sodium phosphate buffer in D_2O (pH 7.6, 100 mM) by ^1H NMR (400 MHz) at 298 K. The corresponding adduct is shown in orange. b) Estimation of the second-order reaction constant (k_{obs}) using the linearly-fitted region of the 1/[**4**]) *versus* time plot. c) Overlay of ^1H NMR spectra at different reaction times. Blue, green and orange arrows point to signals of compounds **4**, **PrSH** and the corresponding adduct, respectively. d) Relative ratio (%) of compounds **4**, **PrSH** and the reaction adduct at different reaction times. e) Estimation of the intrinsic nucleophilic rate constant (k_{Pr-S^-}) from the observed kinetic rate constant derived from the two initial measurements (k_{obs}) (in blue), thiol acidity constant (K_a) and buffer acidity ($[H^+]$).

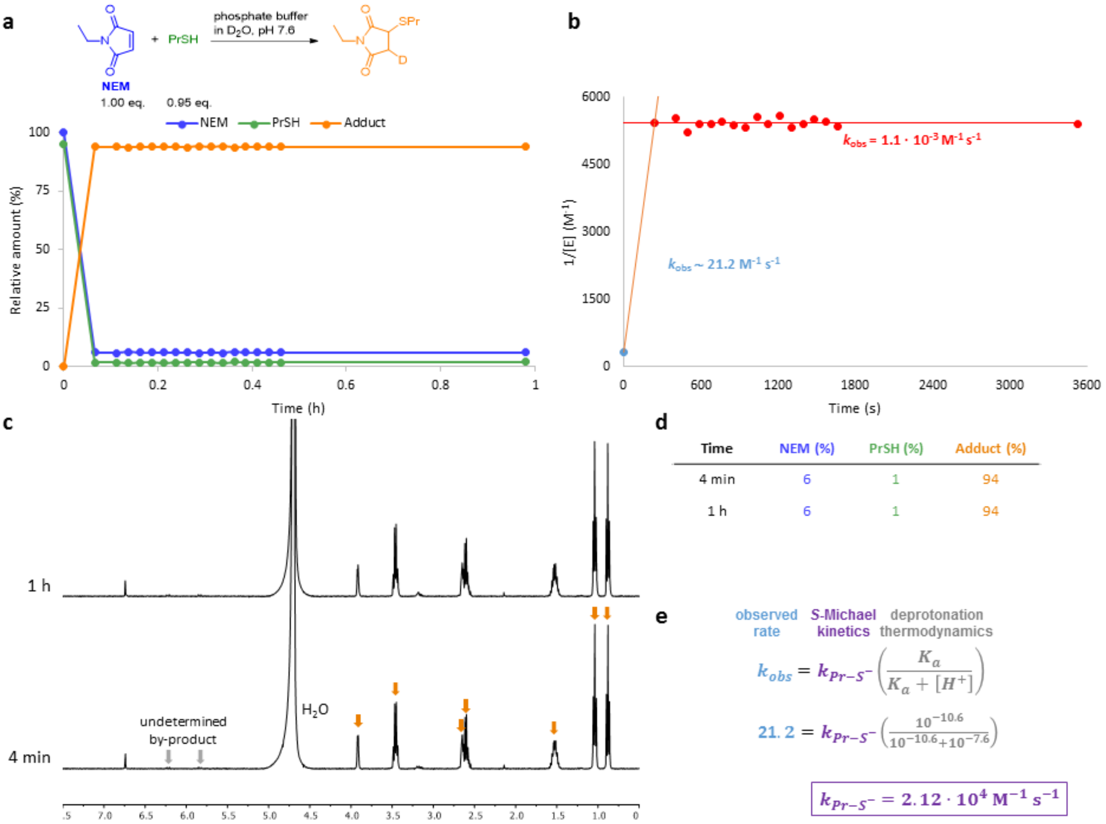

Figure S6. a) Monitoring of reaction between **NEM** (blue) and small-molecule model **PrSH** (green) in sodium phosphate buffer in D_2O (pH 7.6, 100 mM) by ^1H NMR (400 MHz) at 298 K. The corresponding adduct is shown in orange. b) Estimation of the second-order reaction constant (k_{obs}) using the linearly-fitted region of the 1/[**NEM**]) *versus* time plot. c) Overlay of ^1H NMR spectra at different reaction times. Blue, green and orange arrows point to signals of compounds **NEM**, **PrSH** and the corresponding adduct, respectively. d) Relative ratio (%) of compounds **NEM**, **PrSH** and the reaction adduct at different reaction times. e) Estimation of the intrinsic nucleophilic rate constant (k_{Pr-S^-}) from the observed kinetic rate constant derived from the initial measurements (k_{obs}) (in blue), thiol acidity constant (K_a) and buffer acidity ($[H^+]$).

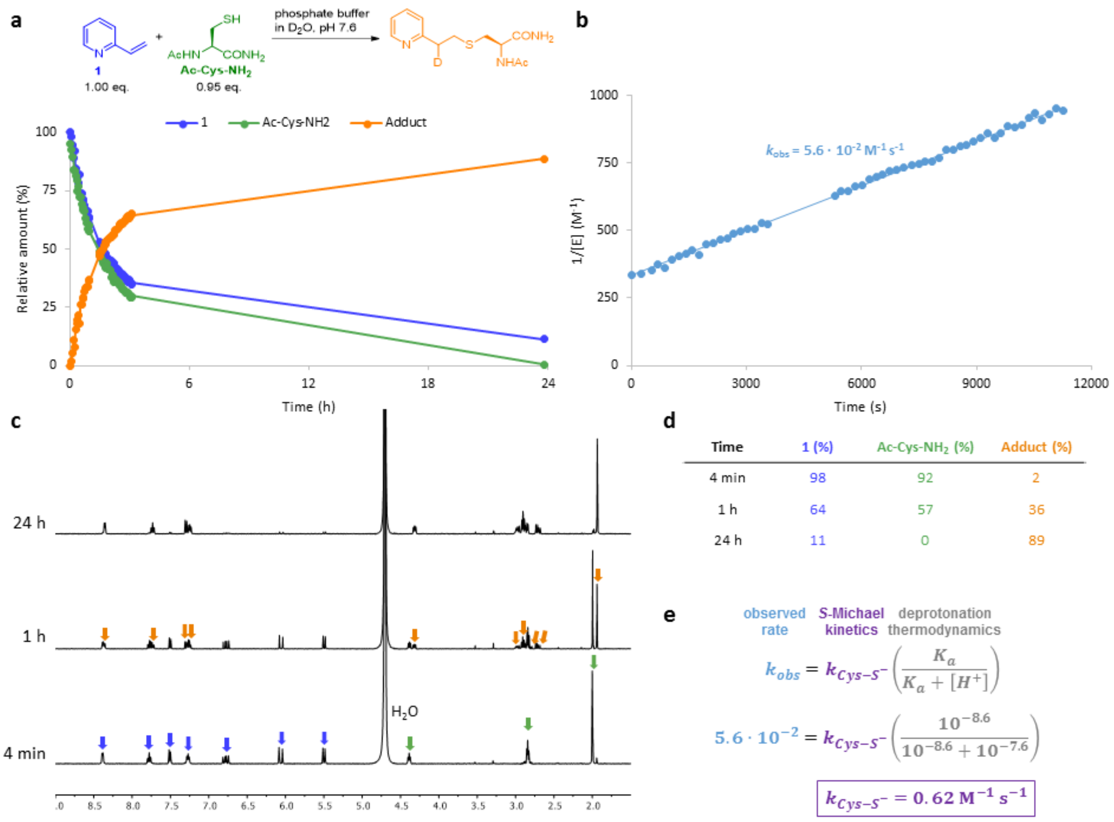

d

Time	1 (%)	Ac-Cys-NH2 (%)	Adduct (%)
4 min	98	92	2
1 h	64	57	36
24 h	11	0	89

$$k_{obs} = k_{Cys-S^-}\left(\frac{K_a}{K_a + [H^+]}\right)$$

$$5.6 \cdot 10^{-2} = k_{Cys-S^-}\left(\frac{10^{-8.6}}{10^{-8.6} + 10^{-7.6}}\right)$$

$$k_{Cys-S^-} = 0.62\ \mathrm{M^{-1}\,s^{-1}}$$

Figure S7. a) Monitoring of reaction between **1** (blue) and **Ac-Cys-NH$_2$** (green) in sodium phosphate buffer in D_2O (pH 7.6, 100 mM) by ^{1}H NMR (400 MHz) at 298 K. The corresponding adduct is shown in orange. b) Estimation of the second-order reaction constant (k_{obs}) using the linearly-fitted region of the 1/[**1**]) *versus* time plot. c) Overlay of ^{1}H NMR spectra at different reaction times. Blue, green and orange arrows point to signals of compounds **1**, **Ac-Cys-NH$_2$** and the corresponding adduct, respectively. d) Relative ratio (%) of compounds **1**, **Ac-Cys-NH$_2$** and the reaction adduct at different reaction times. e) Estimation of the intrinsic nucleophilic rate constant (k_{Pr-S^-}) from the observed kinetic rate constant (k_{obs}) (in blue), cysteine thiol acidity constant (K_a) and buffer acidity ([H^+]).

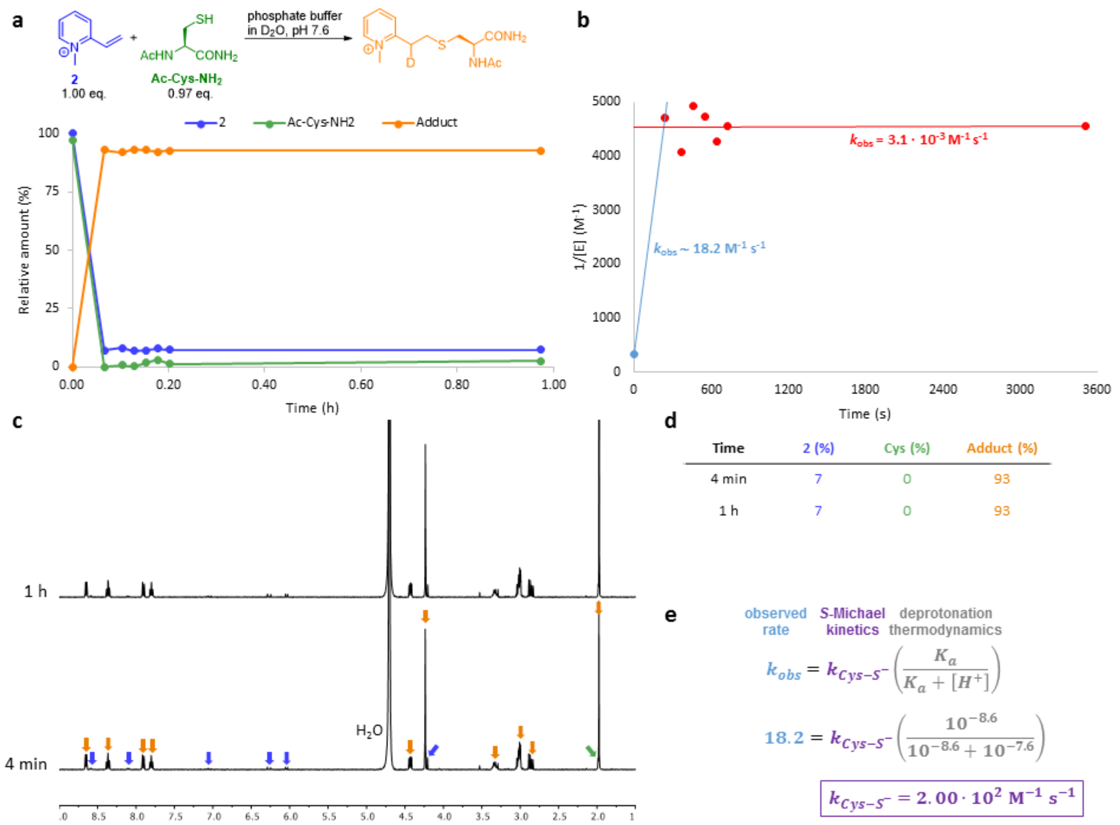

Figure S8. a) Monitoring of reaction between **2** (blue) and **Ac-Cys-NH$_2$** (green) in sodium phosphate buffer in D_2O (pH 7.6, 100 mM) by ^{1}H NMR (400 MHz) at 298 K. The corresponding adduct is shown in orange. b) Estimation of the second-order reaction constant (k_{obs}) using the linearly-fitted region of the 1/[**2**]) *versus* time plot. c) Overlay of ^{1}H NMR spectra at different reaction times. Blue, green and orange arrows point to signals of compounds **2**, **Ac-Cys-NH$_2$** and the corresponding adduct, respectively. d) Relative ratio (%) of compounds **2**, **Ac-Cys-NH$_2$** and the reaction adduct at different reaction times. e) Estimation of the intrinsic nucleophilic rate constant (k_{Pr-S^-}) from the observed kinetic rate constant derived from the two initial measurements (k_{obs}) (in blue), cysteine thiol acidity constant (K_a) and buffer acidity ($[H^+]$).

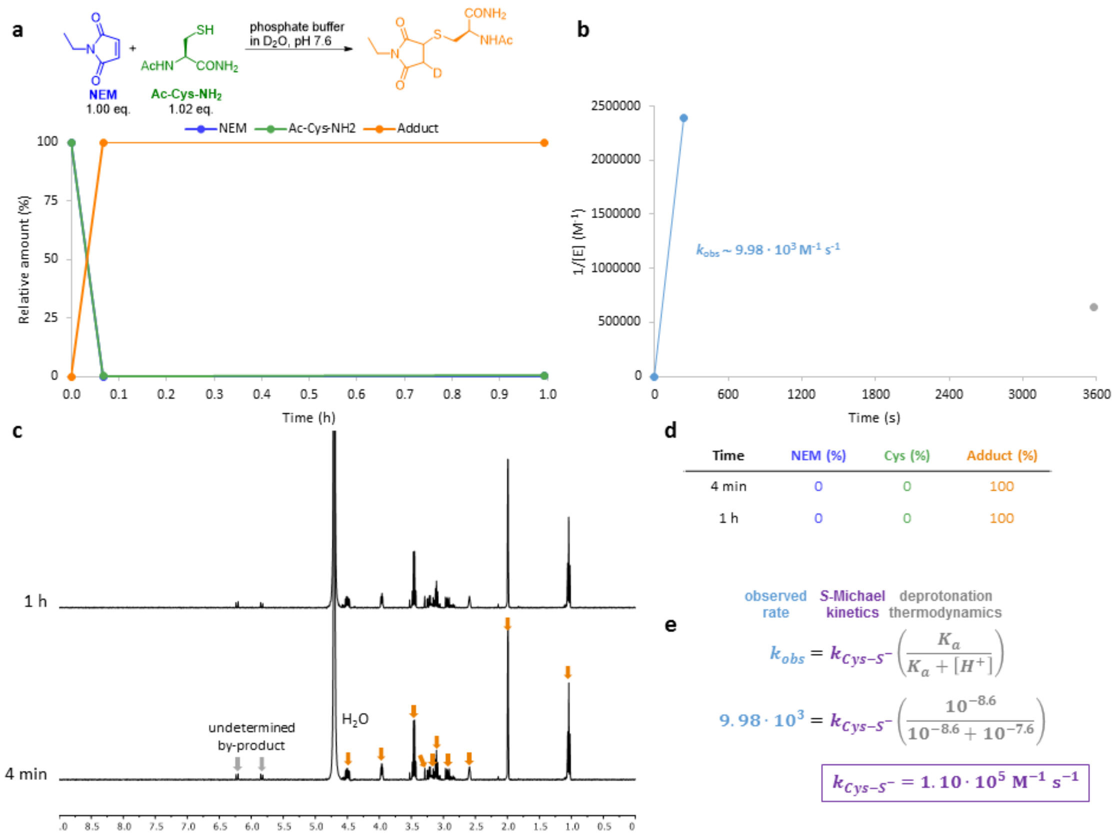

Figure S9. a) Monitoring of reaction between **NEM** (blue) and **Ac-Cys-NH₂** (green) in sodium phosphate buffer in D_2O (pH 7.6, 100 mM) by ^{1}H NMR (400 MHz) at 298 K. The corresponding adduct is shown in orange. b) Estimation of the second-order reaction constant (k_{obs}) using the linearly-fitted region of the 1/[**NEM**]) *versus* time plot. c) Overlay of ^{1}H NMR spectra at different reaction times. Blue, green and orange arrows point to signals of compounds **NEM**, **Ac-Cys-NH₂** and the corresponding adduct, respectively. d) Relative ratio (%) of compounds **NEM**, **Ac-Cys-NH₂** and the reaction adduct at different reaction times. e) Estimation of the intrinsic nucleophilic rate constant (k_{Pr-S^-}) from the observed kinetic rate constant derived from the two initial measurements (k_{obs}) (in blue), cysteine thiol acidity constant (K_a) and buffer acidity ($[H^+]$).

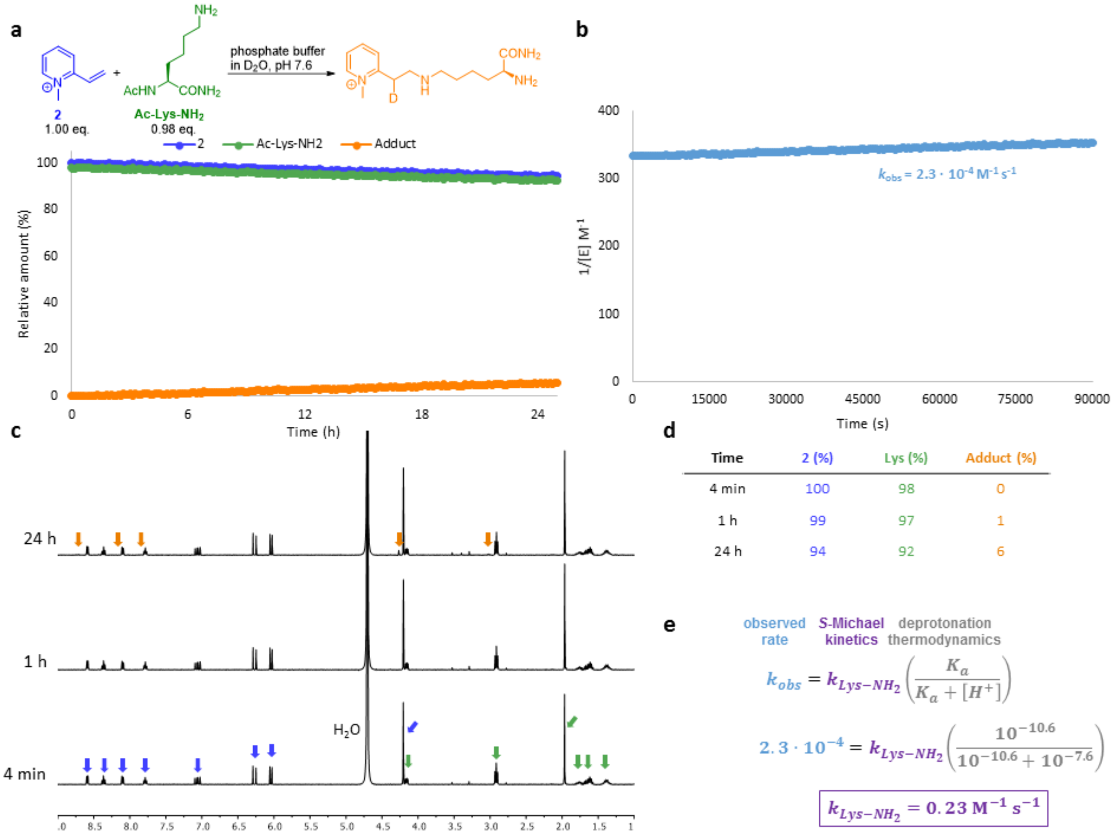

Figure S10. a) Monitoring of reaction between **2** (blue) and **Ac-Lys-NH₂** (green) in sodium phosphate buffer in D_2O (pH 7.6, 100 mM) by ^{1}H NMR (400 MHz) at 298 K. The corresponding adduct is shown in orange. b) Estimation of the second-order reaction constant (k_{obs}) using the linearly-fitted region of the 1/[**2**]) *versus* time plot. c) Overlay of ^{1}H NMR spectra at different reaction times. Blue, green and orange arrows point to signals of compounds **2**, **Ac-Lys-NH₂** and the corresponding adduct, respectively. d) Relative ratio (%) of compounds **2**, **Ac-Lys-NH₂** and the reaction adduct at different reaction times. e) Estimation of the intrinsic nucleophilic rate constant (k_{Pr-NH_2}) from the observed kinetic rate constant (k_{obs}) (in blue), lysine ammonium acidity constant (K_a) and buffer acidity ([H^+]).

pH 5.7

The second-order reaction constants of the reactions of electrophiles **1-4** with small-molecule model *N*-acetylcysteine amide (**Ac-Cys-NH$_2$**) were determined by ^{1}H NMR (400 MHz) at 298 K in sodium phosphate buffer in D_2O (pH 5.7, 100 mM). Electrophile concentration was 3.0 mM in all cases. A ^{1}H NMR spectrum was recorded every 85 s (number of scans: 16). Around 5 min were needed to record the first spectrum after mixing the reagents. The observed second-order rate constants k_{obs} (i.e. k_2) were derived from the slope of a linearly-fitted plot of the inverse of the electrophile concentration (1/[E]) *versus* time.

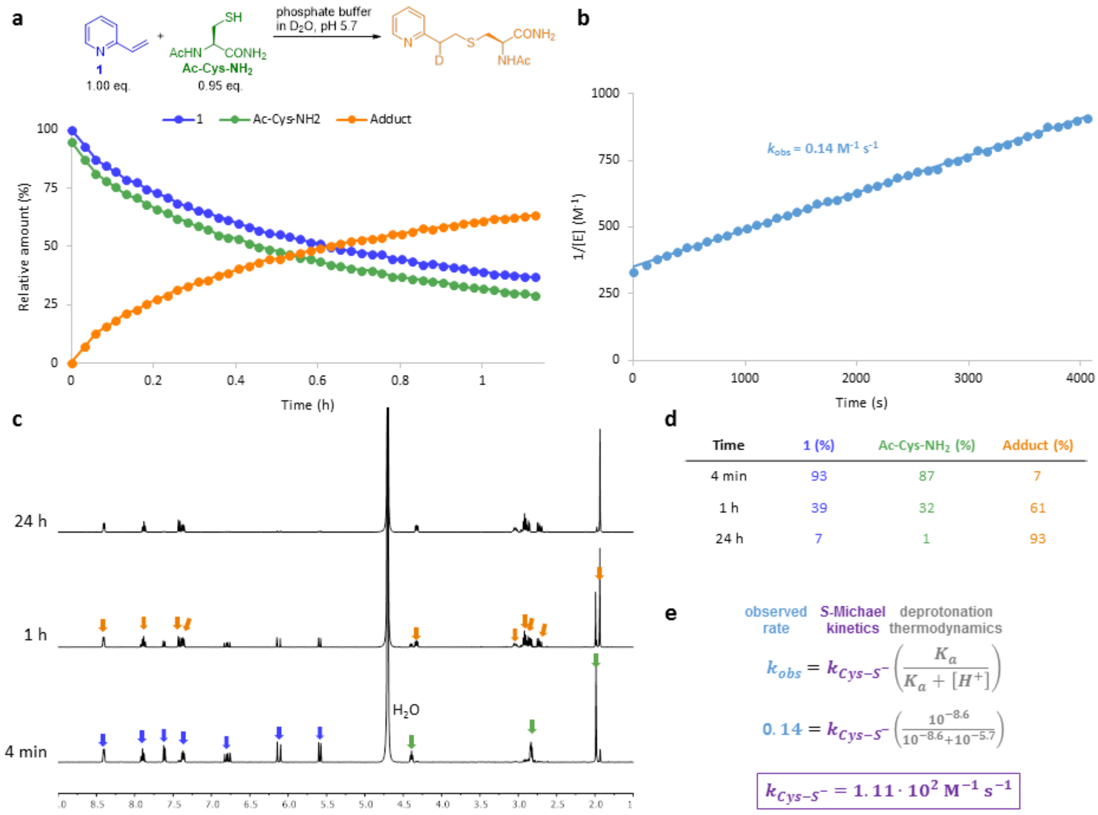

Figure S11. a) Monitoring of reaction between **1** (blue) and **Ac-Cys-NH$_2$** (green) in sodium phosphate buffer in D_2O (pH 5.7, 100 mM) by ^{1}H NMR (400 MHz) at 298 K. The corresponding adduct is shown in orange. b) Estimation of the second-order reaction constant (k_{obs}) using the linearly-fitted regions of the 1/[**1**] *versus* time plot. c) Overlay of ^{1}H NMR spectra at different reaction times. Blue, green and orange arrows point to signals of compounds **1**, **Ac-Cys-NH$_2$** and the corresponding adduct, respectively. d) Relative ratio (%) of compounds **1**, **Ac-Cys-NH$_2$** and the reaction adduct at different reaction times. e) Estimation of the intrinsic nucleophilic rate constant (k_{Pr-S^-}) from the observed kinetic rate constant derived from all the measurements (k_{obs}) (in blue), cysteine thiol acidity constant (K_a) and buffer acidity ($[H^+]$).

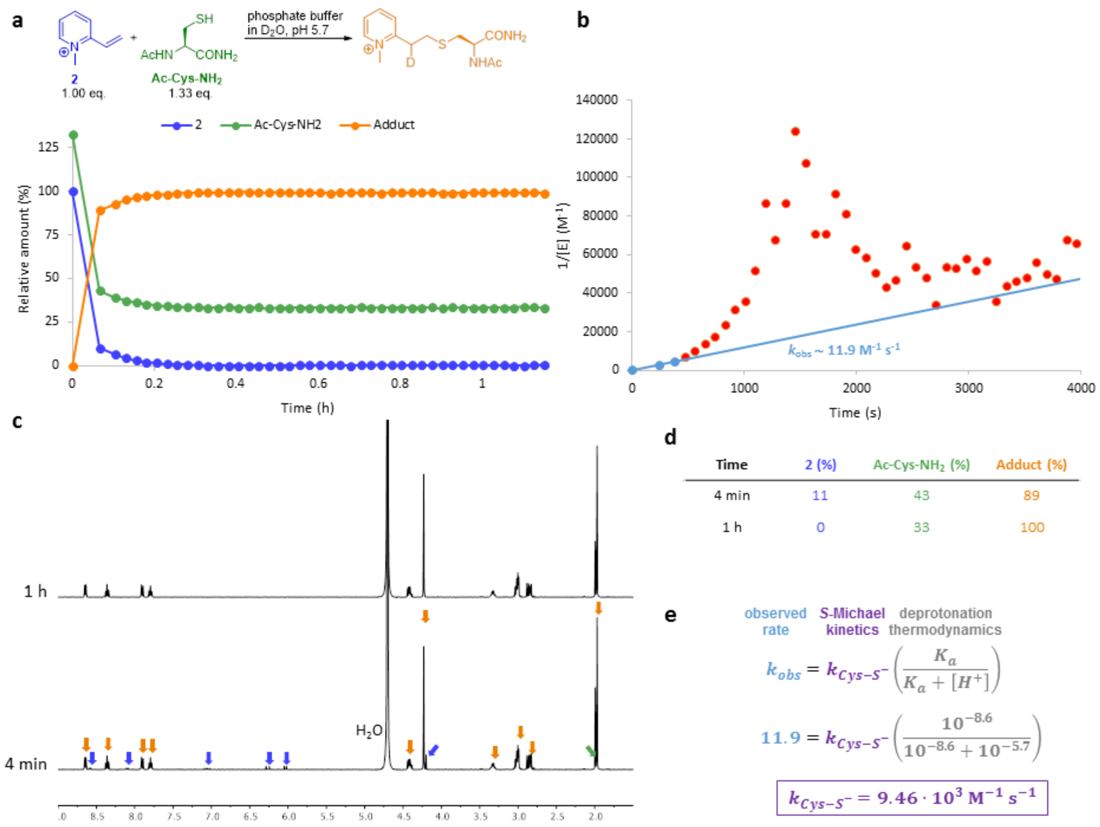

Figure S12. a) Monitoring of reaction between **2** (blue) and **Ac-Cys-NH₂** (green) in sodium phosphate buffer in D_2O (pH 5.7, 100 mM) by ^{1}H NMR (400 MHz) at 298 K. The corresponding adduct is shown in orange. b) Estimation of the second-order reaction constant (k_{obs}) using the linearly-fitted regions of the 1/[**2**] *versus* time plot. c) Overlay of ^{1}H NMR spectra at different reaction times. Blue, green and orange arrows point to signals of compounds **2**, **Ac-Cys-NH₂** and the corresponding adduct, respectively. d) Relative ratio (%) of compounds **2**, **Ac-Cys-NH₂** and the reaction adduct at different reaction times. e) Estimation of the intrinsic nucleophilic rate constant (k_{Pr-S^-}) from the observed kinetic rate constant derived from the initial measurements (k_{obs}) (in blue), cysteine thiol acidity constant (K_a) and buffer acidity ($[H^+]$).

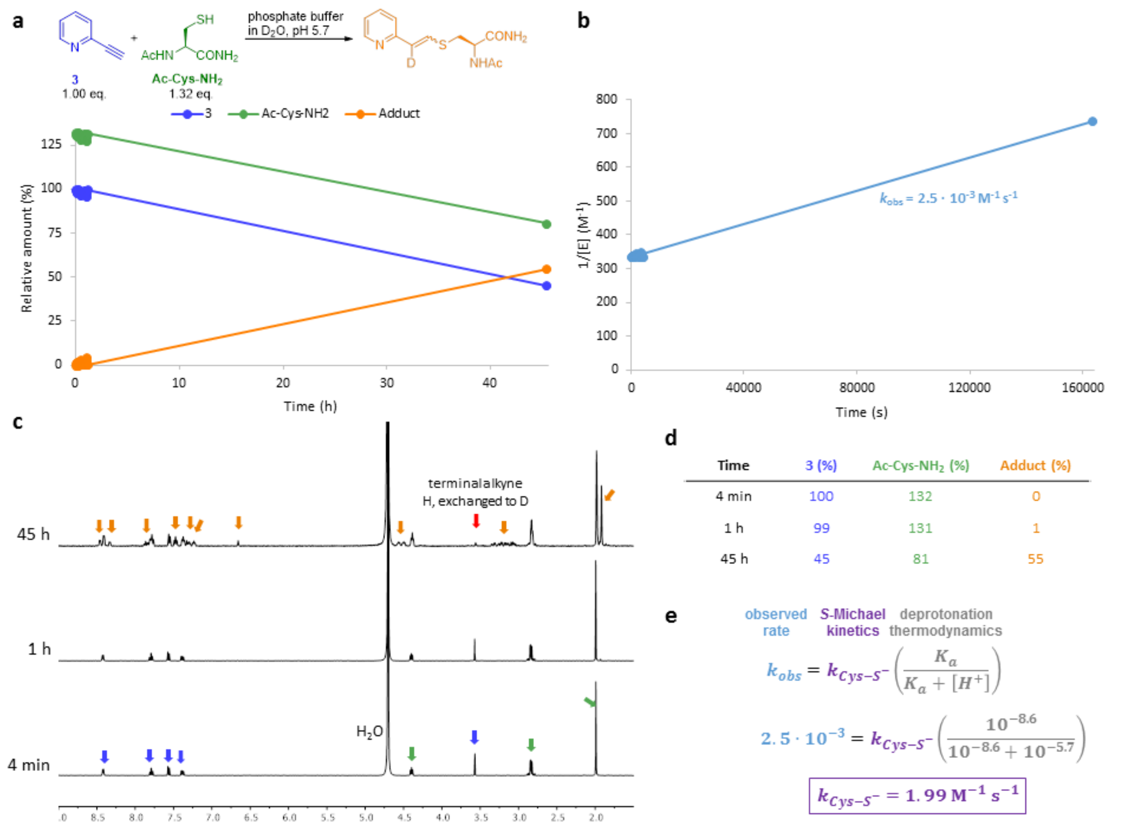

d

Time	3 (%)	Ac-Cys-NH2 (%)	Adduct (%)
4 min	100	132	0
1 h	99	131	1
45 h	45	81	55

e

observed rate — S-Michael kinetics — deprotonation thermodynamics

$$k_{obs} = k_{Cys-S^-}\left(\frac{K_a}{K_a + [H^+]}\right)$$

$$2.5 \cdot 10^{-3} = k_{Cys-S^-}\left(\frac{10^{-8.6}}{10^{-8.6} + 10^{-5.7}}\right)$$

$$k_{Cys-S^-} = 1.99\ \mathrm{M^{-1}\ s^{-1}}$$

Figure S13. a) Monitoring of reaction between **3** (blue) and **Ac-Cys-NH₂** (green) in sodium phosphate buffer in D_2O (pH 5.7, 100 mM) by ^{1}H NMR (400 MHz) at 298 K. The corresponding adduct is shown in orange. b) Estimation of the second-order reaction constant (k_{obs}) using the linearly-fitted regions of the 1/[**3**] *versus* time plot. c) Overlay of ^{1}H NMR spectra at different reaction times. Blue, green and orange arrows point to signals of compounds **3**, **Ac-Cys-NH₂** and the corresponding adduct, respectively. d) Relative ratio (%) of compounds **3**, **Ac-Cys-NH₂** and the reaction adduct at different reaction times. e) Estimation of the intrinsic nucleophilic rate constant (k_{Pr-S^-}) from the observed kinetic rate constant derived from all the measurements (k_{obs}) (in blue), cysteine thiol acidity constant (K_a) and buffer acidity ($[H^+]$).

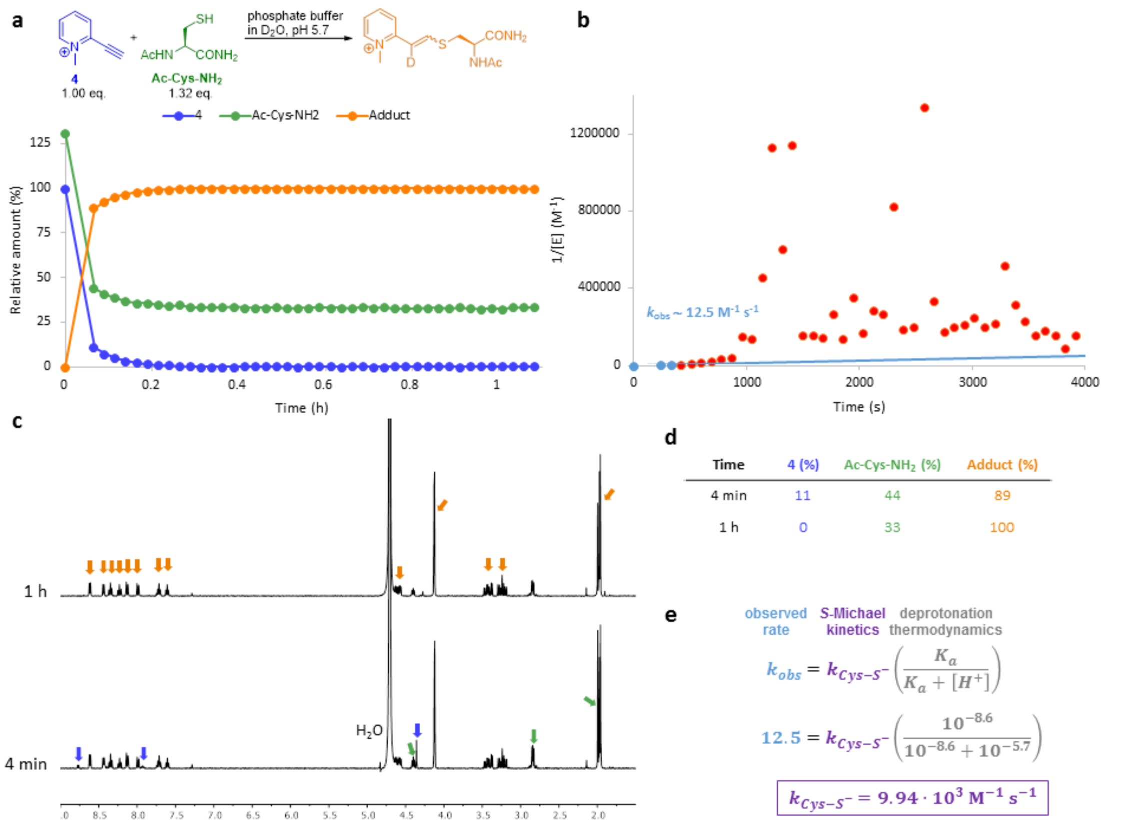

Figure S14. a) Monitoring of reaction between **4** (blue) and **Ac-Cys-NH₂** (green) in sodium phosphate buffer in D_2O (pH 5.7, 100 mM) by ^{1}H NMR (400 MHz) at 298 K. The corresponding adduct is shown in orange. b) Estimation of the second-order reaction constant (k_{obs}) using the linearly-fitted regions of the 1/[**4**] *versus* time plot. c) Overlay of ^{1}H NMR spectra at different reaction times. Blue, green and orange arrows point to signals of compounds **4**, **Ac-Cys-NH₂** and the corresponding adduct, respectively. d) Relative ratio (%) of compounds **4**, **Ac-Cys-NH₂** and the reaction adduct at different reaction times. e) Estimation of the intrinsic nucleophilic rate constant (k_{Pr-S^-}) from the observed kinetic rate constant derived from the initial measurements (k_{obs}) (in blue), cysteine thiol acidity constant (K_a) and buffer acidity ($[H^+]$).

The effect of pH on reactivity

Pyridine protonation might, in principle, accelerate the reaction due to nitrogen quaternization; however, thiol protonation would slow it down due to the decreased concentration of nucleophilic thiolate in solution. Therefore, as pH becomes more acidic, the concentration of the more reactive pyridinium electrophile increases while the concentration of the more reactive thiolate nucleophile decreases. Hence, the reaction outcome in terms of kinetics would strongly depend on the relative pKa's of both reagents, providing an opportunity to fine-tune reactivity and, potentially, chemo- and site-selectivity.

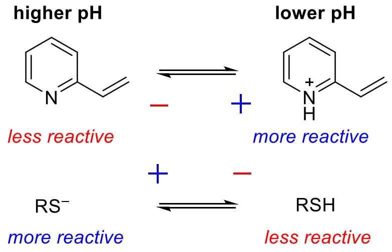

Figure S15. Predominant electrophilic (pyridine/pyridinium) and nucleophilic (thiol/thiolate) species depending on their protonation state as a function of the environmental pH.

To test this concept, additional calculations on the nucleophilic addition of 1-propanethiolate anion to protonated versions of small-molecule models **1** and **3** (labelled as **1'** and **3'**, respectively) were first performed. Of note, PrS^- was able to spontaneously deprotonate alkynyl pyridinium **3'**, giving a largely thermodynamically stable neutral complex (**3'_preTS_SMe**). The activation barrier from this complex to a zwitterionic -and probably unrealistic- transition state (**3'_TS_SMe**) was calculated to be slightly higher than that calculated for the unprotonated derivative ($\Delta G^{\ddagger}$ = 26.1 kcal mol^{-1} for **3'** *vs.* $\Delta G^{\ddagger}$ = 24.3 kcal mol^{-1} for **3**).

On the contrary, PrS^- was computationally unable to deprotonate vinyl pyridinium **1'**, instead forming a less stable hydrogen-bonded complex (**1'_preTS_SMe**). The activation barrier from this complex to the corresponding zwitterionic transition state (**1'_TS_SMe**) was calculated to be much lower than that calculated for the unprotonated derivative, and virtually identical to that calculated for the methylated analogue ($\Delta G^{\ddagger}$ = 10.3 kcal mol^{-1} for **1'** *vs.* $\Delta G^{\ddagger}$ = 25.6 kcal mol^{-1} for **1** *vs.* $\Delta G^{\ddagger}$ = 10.4 kcal mol^{-1} for **2**).

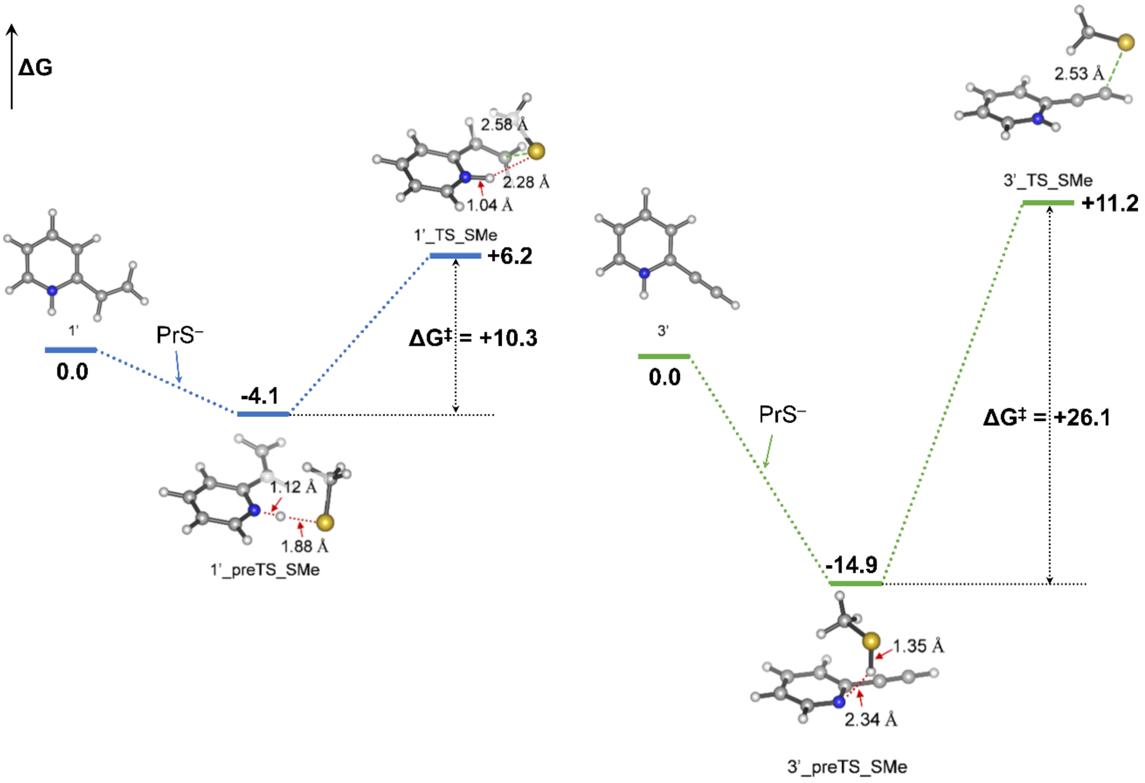

Figure S16. Formation of pyridinium-thiolate complexes and nucleophilic addition transition states for protonated vinyl pyridinium **1'** and protonated alkynyl pyridinium **3'** calculated with PCM(H_2O)/M06-2X/6-31+G(d,p). Relative free energies (ΔG) are in kcal mol^{-1}.

These computational predictions suggest that alkynyl pyridines should not be very reactive at slightly acidic pH likely due to their higher acidity, while vinyl pyridines should be quite reactive do to (partial) quaternisation.

We verified these predictions by performing the reactions between electrophiles **1-4** and cysteine model **Ac-Cys-NH$_2$** at pH 5.7 (see section *1. Reaction kinetics pH 5.7*). In agreement with the calculations, vinyl pyridine **1** was significantly more reactive at a slightly acidic than at neutral pH, achieving a 61% yield of the addition product within 1 h (10% at pH 7.6). On the other hand, alkynyl pyridine **3** was much less reactive (0-1% yield of addition product at both pH 5.7 and 7.6) than the vinyl pyridine analogue.

3. Quantum Mechanical Calculations

Computational Details. Full geometry optimizations were carried out with Gaussian 16[1] using the M06-2X hybrid functional[2] and 6-31+G(d,p) basis set in combination with ultrafine integration grids. Bulk solvent effects in water were considered implicitly through the IEF-PCM polarizable continuum model.[3] The possibility of different conformations was taken into account. Frequency analyses were carried out at the same level used in the geometry optimizations, and the nature of the stationary points was determined in each case according to the appropriate number of negative eigenvalues of the Hessian matrix. The quasiharmonic approximation reported by Trular et al. was used to replace the harmonic oscillator approximation for the calculation of the vibrational contribution to enthalpy and entropy.[4] Scaled frequencies were not considered. Mass-weighted intrinsic reaction coordinate (IRC) calculations were carried out by using the Gonzalez and Schlegel scheme[5] in order to ensure that the TSs indeed connected the appropriate reactants and products. Gibbs free energies (ΔG) were used for the discussion on the relative stabilities of the considered structures. Free energies calculated using the gas phase standard state concentration (1 atm = 1/24.5 M) were converted to reproduce the standard state concentration in solution (1 M) by adding or subtracting 1.89 kcal mol^{-1} for bimolecular additions and decompositions, respectively. The lowest energy conformer for each calculated stationary point was considered in the discussion; all the computed structures can be obtained from authors upon request. Cartesian coordinates, electronic energies, entropies, enthalpies, Gibbs free energies, and lowest frequencies of the calculated structures are available below.

Figure S17. Lowest-energy structures calculated with PCM(H_2O)/M06-2X/6-31+G(d,p)

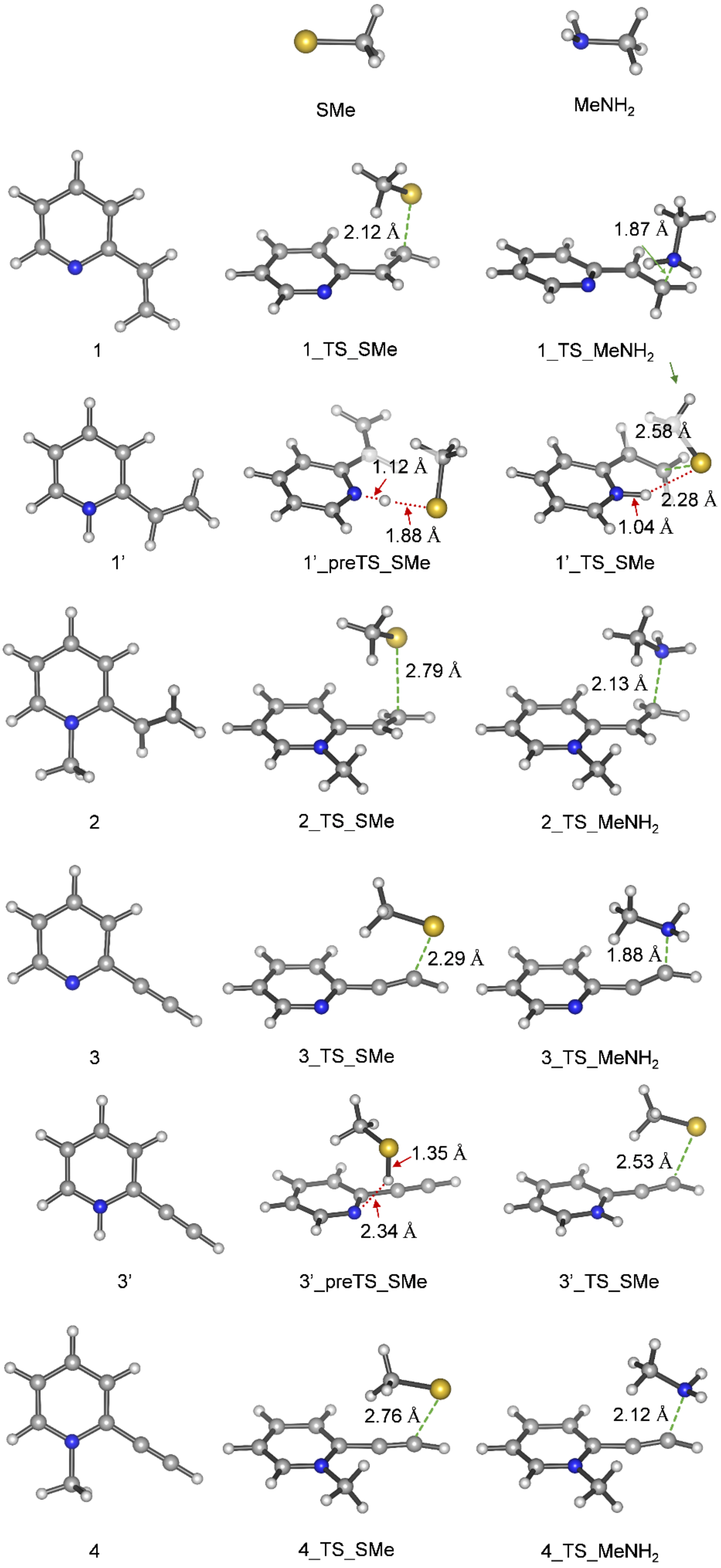

Table of energies, entropies and lowest frequencies of the calculated lowest-energy structures

Structure	E_{elec} (Hartree)[a]	E_{elec} + ZPE (Hartree)[a]	H (Hartree)[a]	S (cal mol^{-1} K^{-1})[b]	G (Hartree)[a,b]	Lowest freq. (cm^{-1})	# of imag freq.
**$MeNH_2$**	-95.815844	-95.751246	-95.746882	57.4	-95.774173	304.0	0
**SMe**	-438.170102	-438.133140	-438.129148	58.1	-438.156747	713.4	0
**1**	-325.563590	-325.440880	-325.433396	80.7	-325.471567	82.1	0
**1_TS_SMe**	-763.715803	-763.555550	-763.544811	100.0	-763.590562	-196.0	1
**1_TS_$MeNH_2$**	-421.354533	-421.164339	-421.153692	97.7	-421.199056	-415.3	1
**1'**	-326.004604	-325.867664	-325.860001	82.3	-325.898358	45.5	0
**1'_preTS_SMe**	-764.199415	-764.027527	-764.015248	110.2	-764.064641	17.7	0
**1'_TS_SMe**	-764.187139	-764.012918	-764.002175	97.9	-764.048190	-222.2	1
**2**	-365.293064	-365.128040	-365.119063	87.3	-365.160518	99.8	0
**2_TS_SMe**	-803.469636	-803.266810	-803.254345	106.8	-803.303701	-140.5	1
**2_TS_$MeNH_2$**	-461.104480	-460.872174	-460.859751	106.6	-460.908418	-272.5	1
**3**	-324.314151	-324.215377	-324.208216	78.9	-324.245694	139.4	0
**3_TS_SMe**	-762.467473	-762.331258	-762.320343	102.2	-762.366670	-267.3	1
**3_TS_$MeNH_2$**	-420.102720	-419.936747	-419.926094	98.7	-419.971270	-411.6	1
**3'**	-324.749772	-324.637040	-324.629739	79.5	-324.667503	138.6	0
**3'_preTS_SMe**	-762.960290	-762.813864	-762.801358	110.0	-762.851039	24.7	0
**3'_TS_SMe**	-762.923835	-762.773831	-762.762840	101.6	-762.809393	-253.0	1
**4**	-364.041521	-363.900763	-363.891949	86.5	-363.933071	119.9	0
**4_TS_SMe**	-802.214030	-802.036055	-802.023336	109.6	-802.073352	-205.0	1
**4_TS_$MeNH_2$**	-459.849653	-459.642499	-459.629905	107.4	-459.679152	-315.3	1

[a]Energy values calculated with PCM(H_2O)/M06-2X/6-31+G(d,p). 1 Hartree = 627.51 kcal mol^{-1}.
[b]Thermal corrections at 298.15 K.

Cartesian coordinates of the lowest-energy calculated structures

Structure SMe

C	1.132156	0.000007	0.000017
H	1.528559	0.652767	0.783142
H	1.528179	-1.004843	0.173660
H	1.528499	0.351880	-0.956885
S	-0.711136	0.000010	-0.000001

Structure $MeNH_2$

C	-0.708121	0.000000	0.017287
H	-1.118454	-0.881675	-0.480975
H	-1.118454	0.881676	-0.480974
H	-1.061351	-0.000001	1.056829
N	0.751111	0.000000	-0.125811
H	1.144607	0.811873	0.341037
H	1.144607	-0.811873	0.341037

Structure 1

C	1.200026	-1.323664	0.000117
C	-0.480297	0.241293	-0.000015
C	0.450979	1.287105	0.000042
C	1.809810	0.987035	-0.000093
C	2.199092	-0.348486	-0.000092
H	1.467211	-2.377786	0.000127
H	0.106050	2.316198	0.000145
H	2.549039	1.781684	-0.000175
H	3.244281	-0.636186	-0.000245
N	-0.104372	-1.049213	0.000129
C	-1.925805	0.549925	0.000112
H	-2.178558	1.608212	0.000409
C	-2.896223	-0.367482	-0.000179
H	-2.662476	-1.427534	-0.000440
H	-3.940434	-0.074454	-0.000072

Structure 1_TS_SMe

C	-2.906352	0.454553	0.632220
C	-0.707877	0.668952	-0.112166
C	-0.833649	-0.597636	-0.774313
C	-2.026025	-1.289268	-0.728919
C	-3.115081	-0.761359	-0.015822
H	-3.714564	0.894592	1.217489
H	0.024501	-1.017109	-1.290657
H	-2.114110	-2.248407	-1.233989
H	-4.067968	-1.273488	0.049455
N	-1.773126	1.155394	0.605800
C	0.484066	1.429560	-0.158322
H	0.553575	2.282444	0.511379
C	1.620198	1.049807	-0.933209
H	1.412072	0.541241	-1.873786
H	2.365280	1.830644	-1.065082
S	2.866766	-0.445095	-0.102735
C	1.954989	-0.835226	1.411688
H	1.504807	-1.829451	1.363747
H	2.605360	-0.773793	2.286199
H	1.153052	-0.089211	1.511458

Structure $1\_TS\_MeNH_2$

C	1.727276	-1.381626	0.293491
C	0.457255	0.564353	0.248267
C	1.570798	1.265018	-0.298403
C	2.757739	0.603395	-0.531409
C	2.858062	-0.764018	-0.224407
H	1.753517	-2.443706	0.534201
H	1.470011	2.324376	-0.516617
H	3.608551	1.138120	-0.945101
H	3.770758	-1.324422	-0.390378
N	0.557816	-0.768679	0.516282
C	-0.790289	1.216651	0.504035
H	-0.962468	2.193162	0.063265
C	-1.837812	0.528756	1.129761
H	-1.582785	-0.207947	1.889797
H	-2.749975	1.082635	1.342164
C	-2.937894	-0.216931	-1.300041
H	-2.140998	0.463767	-1.614066
H	-3.073747	-0.991659	-2.057286
H	-3.863964	0.349009	-1.186121
N	-2.556822	-0.774533	-0.003451
H	-3.318158	-1.281519	0.443330
H	-1.748503	-1.392921	-0.080770

Structure 1'

C	-1.710877	-1.031559	0.013861
C	0.517669	-0.167186	-0.013300
C	0.002334	1.129420	-0.019882
C	-1.370655	1.327954	-0.005899
C	-2.246208	0.237274	0.011425
H	-2.298950	-1.939584	0.025545
H	0.678779	1.973745	-0.039284
H	-1.765497	2.337768	-0.010934
H	-3.320029	0.368403	0.021285
N	-0.372532	-1.185901	0.001201
C	1.937096	-0.531730	-0.024763
C	2.939897	0.347940	0.031310
H	3.966189	-0.000197	0.018560
H	2.785164	1.420028	0.093785
H	2.155569	-1.595255	-0.078877
H	-0.009033	-2.136274	0.005000

Structure 1'_preTS_SMe

C	-0.926039	-1.761724	-0.052930
C	-0.871525	0.600062	-0.164659
C	-2.250374	0.642852	0.055707
C	-2.953776	-0.541593	0.229802
C	-2.288486	-1.769293	0.177467
H	-0.319270	-2.658668	-0.113688
H	-2.762970	1.596491	0.077717
H	-4.024411	-0.510757	0.400319
H	-2.813235	-2.707085	0.306982
N	-0.270169	-0.604973	-0.216923
C	-0.002431	1.770505	-0.355377
H	0.996919	1.550680	-0.725636

C	-0.365910	3.025848	-0.082269
H	0.327517	3.843206	-0.244973
H	-1.343402	3.281171	0.315378
S	2.706152	-0.834280	-0.471931
C	2.841601	0.159813	1.068611
H	3.144325	1.187669	0.851943
H	3.577773	-0.275825	1.747882
H	1.882004	0.196235	1.597524
H	0.829137	-0.678648	-0.402209

Structure 1'_TS_SMe

C	-1.521173	-1.340944	-0.690230
C	-0.717267	0.817334	-0.029289
C	-1.976051	1.079354	0.556277
C	-2.983261	0.144371	0.476407
C	-2.764949	-1.091364	-0.171333
H	-1.243741	-2.271062	-1.170452
H	-2.128828	2.035043	1.044197
H	-3.953657	0.358738	0.911567
H	-3.544764	-1.837425	-0.248404
N	-0.548351	-0.403376	-0.608736
C	0.397045	1.711943	-0.030360
H	0.418583	2.480925	0.733805
C	1.452309	1.517082	-0.884486
H	2.313388	2.172817	-0.834291
H	1.318953	0.989001	-1.821548
S	2.615387	-0.681897	-0.206076
C	1.946191	-0.728853	1.483879
H	2.743905	-0.696908	2.229087
H	1.346655	-1.628152	1.649691
H	1.296298	0.145068	1.646845
H	0.428406	-0.667600	-0.847324

Structure 2

C	-1.678564	0.770283	-0.091814
C	0.476750	-0.182329	0.125366
C	-0.100721	-1.451292	0.146462
C	-1.475032	-1.601945	0.034140
C	-2.280059	-0.469939	-0.081963
H	-2.241584	1.690007	-0.183067
H	0.544786	-2.310453	0.278131
H	-1.916854	-2.591792	0.051279
H	-3.357214	-0.536429	-0.161888
N	-0.337655	0.901361	0.005801
C	0.242282	2.260293	-0.036152
H	1.030759	2.288659	-0.786726
H	-0.545050	2.959527	-0.303497
H	0.641368	2.514418	0.946043
C	1.928374	0.023617	0.244747
C	2.812427	-0.826952	-0.282195
H	3.876220	-0.658849	-0.158851
H	2.507076	-1.693650	-0.861115
H	2.271332	0.898618	0.787530

Structure 2_TS_SMe

C	-2.808860	-0.369699	0.322672
C	-0.631550	0.351327	-0.312258
C	-0.326911	-0.980011	-0.660921
C	-1.268366	-1.977502	-0.528083
C	-2.543089	-1.669022	-0.028455
H	-3.764509	-0.058458	0.724088
H	0.686666	-1.198021	-0.981513
H	-1.017260	-2.999756	-0.789770
H	-3.309566	-2.422404	0.097013
N	-1.879665	0.608878	0.180959
C	-2.242543	1.984328	0.560096
H	-2.046189	2.650488	-0.280061
H	-3.300245	2.007575	0.809166
H	-1.656492	2.293569	1.426528
C	0.337171	1.415997	-0.434932
H	0.226652	2.296875	0.186113
C	1.418750	1.298678	-1.246768
H	1.499414	0.514946	-1.988066
H	2.133665	2.108551	-1.316349
S	3.289744	-0.334756	0.019872
C	2.243557	-0.490693	1.504268
H	1.823945	-1.497351	1.598560
H	2.802933	-0.264770	2.416077
H	1.400992	0.215098	1.450256

Structure 2_TS_MeNH$_2$

C	-2.572692	0.032602	0.385077
C	-0.310561	0.218740	-0.371070
C	-0.325851	-1.182676	-0.590640
C	-1.444912	-1.934928	-0.334232
C	-2.605394	-1.317186	0.170194
H	-3.420515	0.581538	0.774002
H	0.576077	-1.659152	-0.952824
H	-1.428388	-3.005117	-0.509602
H	-3.504811	-1.875253	0.393528
N	-1.463509	0.775824	0.119288
C	-1.524076	2.223996	0.365168
H	-1.291314	2.758769	-0.556374
H	-2.529685	2.477750	0.689931
H	-0.810232	2.496416	1.144021
C	0.827636	1.043678	-0.628141
H	0.827926	2.066544	-0.278135
C	1.963675	0.565511	-1.242380
H	1.956273	-0.356039	-1.812781
H	2.730669	1.274528	-1.530140
C	2.562987	-0.539245	1.438492
H	1.949849	-1.437925	1.354230
H	3.237824	-0.647760	2.291772
H	1.897317	0.311724	1.619625
N	3.275051	-0.320237	0.182530
H	4.037365	0.342119	0.297332
H	3.665983	-1.180197	-0.192125

Structure 3

C	1.399412	-1.209499	-0.000212
C	-0.550411	-0.011462	0.000809
C	0.139383	1.206342	0.000165
C	1.530002	1.180532	-0.000137
C	2.177221	-0.051148	-0.000198
H	1.872735	-2.187696	-0.000135
H	-0.411266	2.140085	0.000056

H	2.095662	2.106289	-0.000355
H	3.258896	-0.121803	-0.000471
N	0.064976	-1.206150	0.000180
C	-1.993616	-0.019432	0.000080
C	-3.201927	-0.010178	-0.000455
H	-4.271246	-0.004753	-0.000667

Structure 3_TS_SMe

C	2.575113	0.255691	-1.187345
C	0.772530	-0.623784	-0.038666
C	1.414596	-0.341832	1.198532
C	2.656811	0.261624	1.196942
C	3.267792	0.577049	-0.024403
H	3.014482	0.478562	-2.158228
H	0.914804	-0.601315	2.125886
H	3.152342	0.487500	2.136881
H	4.242458	1.048255	-0.072702
N	1.372010	-0.325591	-1.222117
C	-0.488702	-1.259581	-0.060805
C	-1.722438	-1.474036	-0.041352
H	-2.447221	-2.269356	-0.044653
C	-2.026014	1.578479	-0.006505
H	-2.077386	2.190458	0.897207
H	-1.025813	1.129504	-0.062791
H	-2.162774	2.225284	-0.876333
S	-3.244569	0.234287	0.024447

Structure 3_TS_MeNH$_2$

C	2.143170	0.235116	-1.197083
C	0.353787	-0.653109	-0.040535
C	1.013560	-0.408985	1.190626
C	2.261970	0.184706	1.183806
C	2.855925	0.521748	-0.038443
H	2.567562	0.476147	-2.169885
H	0.526919	-0.687704	2.119484
H	2.773996	0.384580	2.120606
H	3.834436	0.984529	-0.090882
N	0.932516	-0.334170	-1.222829
C	-0.920086	-1.287892	-0.063886
C	-2.178007	-1.216534	-0.051869
H	-3.060956	-1.834385	-0.067644
C	-2.058538	1.535508	0.082276
H	-1.483968	1.411917	1.001172
H	-1.376267	1.448317	-0.764459
H	-2.533874	2.518483	0.074669
N	-3.045053	0.454169	-0.001118
H	-3.613726	0.533211	-0.842396
H	-3.677048	0.461573	0.797622

Structure 3'

C	1.428674	-1.201219	-0.000028
C	-0.578281	0.072438	0.000107
C	0.157743	1.251817	-0.000020
C	1.548611	1.185223	0.000015
C	2.194395	-0.050398	0.000009
H	1.840873	-2.201366	-0.000101
H	-0.367303	2.198151	-0.000128
H	2.128927	2.100923	-0.000032
H	3.273531	-0.128301	-0.000031
N	0.089518	-1.107143	0.000015
H	-0.457939	-1.965807	-0.000025
C	-2.003094	0.032230	-0.000016
C	-3.208934	0.004265	-0.000057
H	-4.279393	-0.019731	0.000150

Structure 3'_preTS_SMe

C	1.272449	-1.113648	1.169417
C	0.778566	0.893358	0.182313
C	1.708672	0.676852	-0.840813
C	2.438475	-0.506968	-0.832187
C	2.215787	-1.425552	0.189881
H	1.073159	-1.806456	1.982503
H	1.838993	1.419666	-1.619532
H	3.163641	-0.709333	-1.613206
H	2.756764	-2.363749	0.234695
N	0.563589	0.017421	1.178354
C	-0.029052	2.088643	0.183296
C	-0.712222	3.085252	0.162702
S	-2.640897	-0.889212	0.179304
C	-1.565902	-0.912179	-1.293264
H	-2.102159	-1.466239	-2.064181
H	-0.625494	-1.423404	-1.078204
H	-1.369611	0.099229	-1.651162
H	-1.750132	-0.326261	1.015199
H	-1.316582	3.967437	0.148485

Structure 3'_TS_SMe

C	-2.647914	-0.187766	-1.211446
C	-0.798198	0.603263	0.105289
C	-1.494714	0.196714	1.270723
C	-2.728994	-0.398701	1.165489
C	-3.328547	-0.604215	-0.097569
H	-3.020548	-0.289647	-2.222568
H	-1.020231	0.357647	2.232052
H	-3.249525	-0.714739	2.062634
H	-4.297384	-1.071729	-0.198020
N	-1.433210	0.397866	-1.086678
C	0.457137	1.210804	0.113654
C	1.641914	1.573820	0.107169
H	2.381016	2.368268	0.139342
S	3.416281	-0.222246	-0.033375
C	2.194951	-1.567021	-0.071120
H	2.327720	-2.259195	0.764084
H	1.183449	-1.136340	0.005562
H	2.243272	-2.135745	-1.005029
H	-0.949602	0.690972	-1.930439

Structure 4

C	1.398951	1.070574	0.000276
C	-0.415770	-0.431503	-0.000109
C	0.463256	-1.509676	0.000003
C	1.834207	-1.280345	0.000279
C	2.307424	0.028284	0.000429
H	1.702956	2.109254	0.000348
H	0.051469	-2.510745	-0.000127
H	2.524201	-2.116245	0.000384

H	3.365832	0.254168	0.000659
N	0.072646	0.839864	0.000013
C	-0.868665	1.978901	-0.000129
H	-1.489802	1.922237	0.893112
H	-0.291694	2.899295	-0.000196
H	-1.489730	1.922066	-0.893406
C	-1.830556	-0.616777	-0.000316
C	-3.022387	-0.804615	-0.000473
H	-4.080514	-0.968144	-0.000631

Structure 4_TS_SMe

C	-2.532996	0.920094	-0.443252
C	-0.784847	-0.321143	0.569311
C	-1.499388	-1.506868	0.325512
C	-2.724954	-1.457522	-0.307260
C	-3.255982	-0.220800	-0.699881
H	-2.880240	1.907636	-0.718796
H	-1.059018	-2.442261	0.646646
H	-3.273742	-2.373238	-0.496606
H	-4.214101	-0.141733	-1.196216
N	-1.330444	0.868436	0.174669
C	-0.573011	2.107765	0.413972
H	0.388562	2.036378	-0.097299
H	-1.146426	2.945845	0.026730
H	-0.409926	2.223210	1.485883
C	0.463132	-0.312673	1.220092
C	1.593791	-0.318254	1.680483
H	2.432423	-0.325423	2.341200
S	3.516423	-0.194178	-0.301980
C	2.219032	-0.026821	-1.570502
H	1.228059	-0.186342	-1.119900
H	2.221107	0.967431	-2.026171
H	2.334982	-0.766369	-2.367328

Structure 4_TS_MeNH$_2$

C	2.357870	0.673745	0.532679
C	0.377776	-0.094403	-0.534180
C	0.956880	-1.378037	-0.642979
C	2.223683	-1.608014	-0.157508
C	2.944542	-0.562260	0.446033
H	2.849301	1.526776	0.982880
H	0.373105	-2.160416	-1.111368
H	2.662604	-2.596166	-0.239580
H	3.941826	-0.707671	0.839261
N	1.109360	0.900669	0.051471
C	0.515499	2.240796	0.157154
H	-0.376599	2.191018	0.783489
H	1.246273	2.913428	0.598551
H	0.240256	2.584148	-0.841063
C	-0.898524	0.194516	-1.025457
C	-2.098459	0.332077	-1.277360
H	-2.915666	0.603593	-1.915757
C	-2.556640	-0.790542	1.347962
H	-2.045783	-1.673467	0.957753
H	-1.797672	-0.054516	1.626103
H	-3.121197	-1.069011	2.242232
N	-3.397126	-0.228262	0.297269
H	-3.907968	0.591536	0.614298
H	-4.069876	-0.903373	-0.056047

4. Synthesis

General remarks

Chemicals were purchased from Fisher Chemicals or Sigma-Aldrich and used without further purification or were prepared in the laboratory and used without additional modification. All solvents were commercially available grade. All reactions were carried out under argon atmosphere unless otherwise mentioned.

Reaction mixtures were analysed by analytical thin-layer chromatography and flash column chromatography was performed on Merck TLC Silica gel 60 F254 glass plates and Silica Gel high purity grade (Merck grade 9385 pore size 60 Å, 230–400 mesh particle size), respectively. Visualization was accomplished with UV light (254 nm), ninhydrin or $KMnO_4$. NMR spectra were recorded using a 400 MHz DPX-400 Dual Spectrometer for the initial reaction studies and during synthesis. NMR spectra were recorded using a 500 MHz AVIII HD Smart Probe Spectrometer for the measurement of kinetics. All chemical shifts are reported in ppm from trimethylsilane, and methanol-*d4*, DMF-*d7* or D_2O were used as solvents. Mass spectroscopy was performed using a Waters micromass ZQ (LCMS) with Waters 2795 HPLC and a Waters 2996 photodiode array detector. This system is an automated service utilizing electrospray (ESI) ionization. The mobile phases are 95% aqueous acetonitrile with 0.05% formic acid and 10 mM ammonium acetate with 0.1% formic acid. The separation technology is based on a 50x4.6 mm C18 column (currently a Phenomenex Kinetix solid core column). There are several methods available enabling the user to produce mass spectra for compounds up to 2kDa in positive and negative modes of ionization. In some cases, a Waters LCT Premier combined with an Agilent 1100 autosampler was also used. The system runs using 50% aqueous acetonitrile with 0.25% formic acid as mobile phase and can measure accurate masses from 150 Da to 1500 Da. UV spectra for recording absorption curves and kinetics of the reactions were recorded using a Cary 300 UV spectrometer. CD measurements were made in a 1 mm cuvette using Applied Photophysics' Chirascan CD spectrometer equipped with a Quantum TC125 temperature control unit 25 ºC.

1-(prop-2-yn-1-yl)-2-vinylpyridin-1-ium trifluoromethanesulfonate

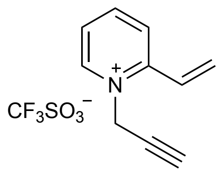

This compound was synthesized following a procedure similar to that described in the literature.[6] Thus, propargyl alcohol (87 µL, 1.5 mmol) and dry pyridine (121 µL, 1.5 mmol) were dissolved in dry toluene (1 mL) under an Argon atmosphere. The resulting solution was added to a solution of Tf_2O (252 µL, 1.5 mmol) in dry toluene (1 mL) at 0 ºC under argon. After stirring for 15 min at this temperature, a white solid is formed and allowed to precipitate. The supernatant was taken with a syringe and transferred to a schlenk. To this propargyl triflate solution, cooled at 0 ºC, a solution of 2-vinylpyridine (107 µL, 1 mmol) in 1 mL of dry toluene was added over 10 min. The reaction

was stirred 10 min at room temperature, and then stopped adding 10 mL of Et_2O for complete product precipitation. The desired product was washed with Et_2O and used for the next steps without further purification (174 mg, 0.59 mmol, 59%). ^1H NMR (300MHz, Methanol-*d4*) δ ppm = 9.08 (dd, *J*=6.3, 1.4 Hz, H-pyr), 8.61 (td, *J*=7.7, 1.5 Hz, H-pyr), 8.39 (dd, *J*=8.2, 1.5 Hz, H-pyr), 8.05 (ddd, *J*=7.8, 6.3, 1.5 Hz, H-pyr), 7.37 (dd, *J*= 17.0, 11.3 Hz, H-vinyl), 6.54 (d, *J*=17.0 Hz, H-vinyl), 6.24 (d, *J*=11.35 Hz, H-vinyl), 5.59 (d, *J*=2.6 Hz, CH_2), 3.50 (t, *J*=2.6 Hz, CH). ^{13}C NMR (75 MHz, Methanol-*d4*) δ ppm = 152.8 (Cq), 146.4 (C-pyr), 144.2 (C-pyr), 129.9 (CH_2 vinyl), 126.7, 126.5 (3C, C-pyr, CH vinyl), 80.1 (C-propargyl), 73.5 (C-propargyl), 47.7 (CH_2-N); MS (ESI^+) *m/z* 144.08 (M^+).

Characterization of *cis* and *trans* alkene mono-adducts derived from thiol addition to alkynyl pyridinium derivatives

Compound **4** (21 mg, 0.084 mmol) was dissolved in sodium phosphate buffer in H_2O (1 mL, pH 7.6, 100 mM) and 1-propanethiol (7.6 µL, 0.084 mmol) was then added. The reaction mixture was stirred for 15 min at room temperature and subsequently purified by semi-preparative HPLC (isocratic 20% solvent B for 5 min, then linear gradient 20% to 40% solvent B in 20 min. Solvent A: 0.1% TFA in H_2O. Solvent B: acetonitrile). The *cis*- and *trans*-alkene derivatives resulting from the *S*-Michael addition to alkynylpyridinium **4** could be initially separated (t_R PrSH-**4**-*cis* 16.6 min, t_R PrSH-**4**-*trans* 17.9 min), but they could not be fully isolated and characterized since they progressively isomerize at room temperature to give a *cis*/*trans* mixture.

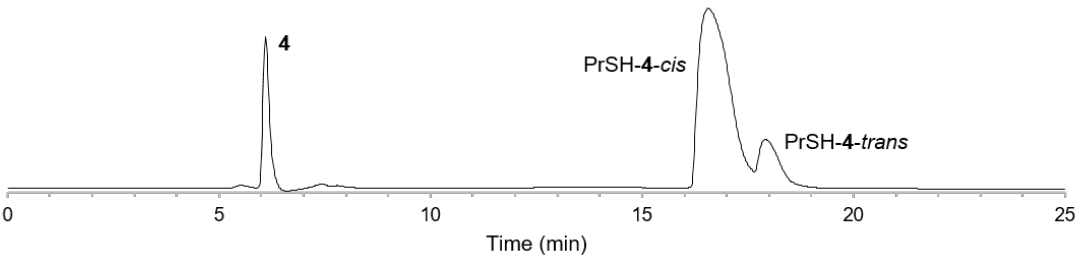

Figure S18. Semi-preparative HPLC chromatograph obtained for the reaction between compound **4** and PrSH (isocratic 20% B for 5 min, then linear gradient 20% to 40% of solvent B in 20 min. Solvent A: 0.1% TFA in H_2O. Solvent B: acetonitrile).

Compound PrSH-**4**-*cis*. HRMS (ESI) (m/z) 194.1002 $[M]^+$, calcd for $C_{11}H_{16}NS^+$: 194.0998. ^1H NMR (400 MHz, D_2O) δ (ppm) 1.00 (t, *J* = 7.4 Hz, 3H, $\underline{CH_3}CH_2CH_2$), 1.75 (qt, *J* = 7.4, 7.2 Hz, 2H, $CH_3\underline{CH_2}CH_2$), 2.99 (t, *J* = 7.2 Hz, 2H, $CH_3CH_2\underline{CH_2}$), 4.21 (s, 3H, N-Me), 6.65 (d, *J* = 11.0 Hz, 1H, Py-$\underline{CH}$=CH-SPr), 7.50 (d, *J* = 11.0 Hz, 1H, Py-CH=$\underline{CH}$-SPr), 7.72-7.78 (m, 1H, H^5), 8.26-8.30 (m, 1H, H^3), 8.38-8.45 (m, 1H, H^4), 8.66 (d, *J* = 6.4 Hz, 1H, H^6). ^{13}C NMR (100 MHz, D_2O) δ (ppm) 12.0 ($\underline{CH_3}CH_2CH_2$), 23.2 ($CH_3\underline{CH_2}CH_2$), 37.4 ($CH_3CH_2\underline{CH_2}$), 45.6 (N-Me), 113.2 (Py-$\underline{CH}$=CH-SPr), 124.4 (C^5), 127.5 (C^3), 144.4 (Py-CH=$\underline{CH}$-SPr), 145.6 (C^4), 145.9 (C^6), 151.2 (C^2).

Compound PrSH-**4**-*trans*. HRMS (ESI) (m/z) 194.1003 $[M]^+$, calcd for $C_{11}H_{16}NS^+$: 194.0998. ^1H NMR (300 MHz, D_2O) δ (ppm) 0.99 (t, *J* = 7.4 Hz, 3H, $\underline{CH_3}CH_2CH_2$), 1.73 (qt, *J* = 7.4, 7.2 Hz,

2H, $CH_3\underline{CH_2}CH_2$), 2.98 (t, J = 7.2 Hz, 2H, $CH_3CH_2\underline{CH_2}$), 4.14 (s, 3H, N-Me), 6.65 (d, J = 15.3 Hz, 1H, Py-$\underline{CH}$=CH-SPr), 7.55-7.62 (m, 1H, H^5), 7.87 (d, J = 15.3 Hz, 1H, Py-CH=$\underline{CH}$-SPr), 7.99-8.03 (m, 1H, H^3), 8.18-8.25 (m, 1H, H^4), 8.43 (d, J = 6.4 Hz, 1H, H^6). ^{13}C NMR (75 MHz, D_2O) δ (ppm) 12.4 ($\underline{CH_3}CH_2CH_2$), 21.9 ($CH_3\underline{CH_2}CH_2$), 34.0 ($CH_3CH_2\underline{CH_2}$), 45.3 (N-Me), 112.4 (Py-$\underline{CH}$=CH-SPr), 123.5 (C^5), 124.1 (C^3), 143.6 (Py-CH=$\underline{CH}$-SPr), 144.4 (C^6), 147.5 (C^4), 152.2 (C^2).

Characterization of a dithioacetal bis-adduct derived from thiol addition to alkynyl pyridinium derivatives

The reaction between electrophile **4** and an excess (2.2 equiv.) of 1-propanethiol was monitored by ^{1}H NMR (400 MHz) at 298 K in sodium phosphate buffer in D_2O (pH 7.6, 100 mM). Electrophile concentration was 3.0 mM. A ^{1}H NMR spectrum was recorded 4 min, 1 hour, and 45 hours (number of scans: 16) after mixing the reagents. The ^{1}H NMR data for the obtained bis-adduct were extracted from the spectrum of the reaction mixture after 45 h.

Compound PrSH-**4**-bis-adduct. ^{1}H NMR (400 MHz, PBS pH 7.6, 100 mM in D_2O) δ (ppm) 0.87-0.98 (m, 6H, 2 x $CH_3CH_2CH_2$), 1.50-1.67 (m, 4H, 2 x $CH_3CH_2CH_2$), 2.61-2.79 (m, 4H, 2 x $CH_3CH_2CH_2$), 4.37 (s, 3H, N-Me), 7.87-7.96 (m, 1H, H5), 8.09 (d, J = 8.6 Hz, 1H, H3), 8.42-8.51 (m, 1H, H4), 8.76 (d, J = 6.3 Hz, 1H, H6).

a

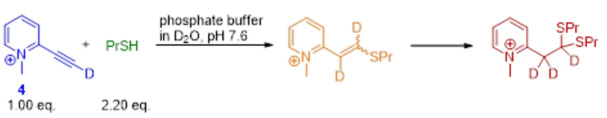

b

Time	4 (%)	PrSH (%)	Adduct (%)	Bis-adduct (%)
4 min	0	116	100	0
1 h	0	105	92	8
45 h	0	21	23	77

c

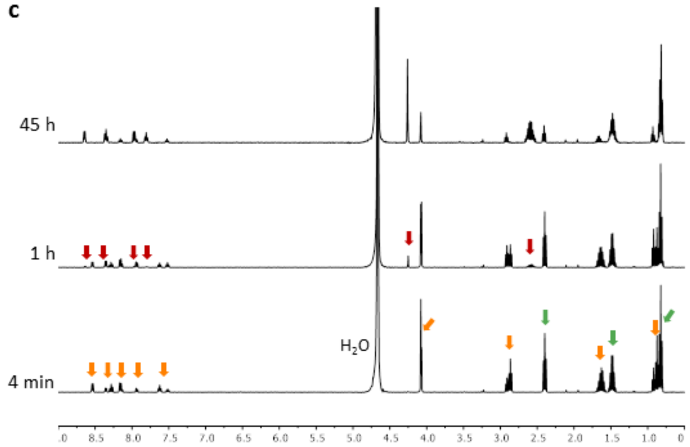

Figure S19. a) Monitoring of reaction between **4** (blue) and 2.2 equivalents of **PrSH** (green) in sodium phosphate buffer in D_2O (pH 7.6, 100 mM) by ^{1}H NMR (400 MHz) at 298 K. The corresponding adduct and subsequent bis-adduct are shown in orange and red, respectively. b) Relative ratio (%) of compounds **4**, **PrSH** and the reaction adduct and bis-adduct at different reaction times. c) Overlay of ^{1}H NMR spectra at different reaction times. Blue, green, orange and red arrows point to signals of compounds **4**, **PrSH** and the corresponding adduct and bis-adduct, respectively.

Synthesis of compound 5

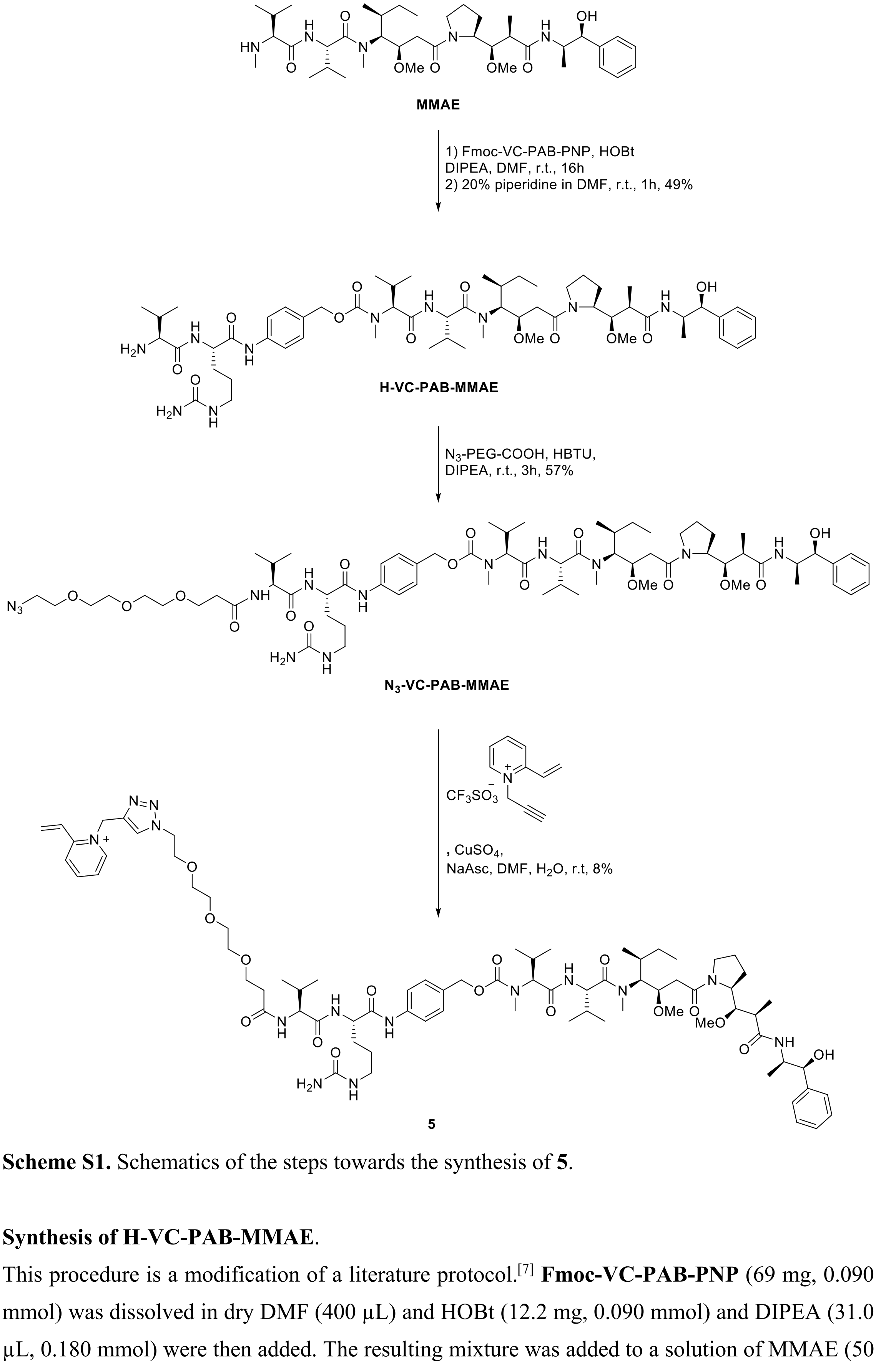

5

Scheme S1. Schematics of the steps towards the synthesis of **5**.

Synthesis of H-VC-PAB-MMAE.

This procedure is a modification of a literature protocol.[7] **Fmoc-VC-PAB-PNP** (69 mg, 0.090 mmol) was dissolved in dry DMF (400 μL) and HOBt (12.2 mg, 0.090 mmol) and DIPEA (31.0 μL, 0.180 mmol) were then added. The resulting mixture was added to a solution of MMAE (50

mg, 0.069 mmol) in dry DMF (100 μL) and stirred for 16 h at room temperature. 300 μL of DMF and 200 μL of piperidine were then added to obtain a final piperidine concentration of 20% in DMF. After stirring for 1 h, Fmoc deprotection was confirmed by MALDI-TOF, and the crude mixture was diluted with CH_3CN and directly purified by HPLC (see Figure bellow for details). Product containing fractions by mass (t_R=13.80) were lyophilized overnight to obtain compound **H-VC-PAB-MMAE** as a white solid (42 mg, 0.034 mmol, 49% yield). HRMS: (m/z) $[M + Na]^+$ calcd. for $C_{58}H_{95}N_{10}NaO_{12}$, 1123.7125; found 1123.7103.

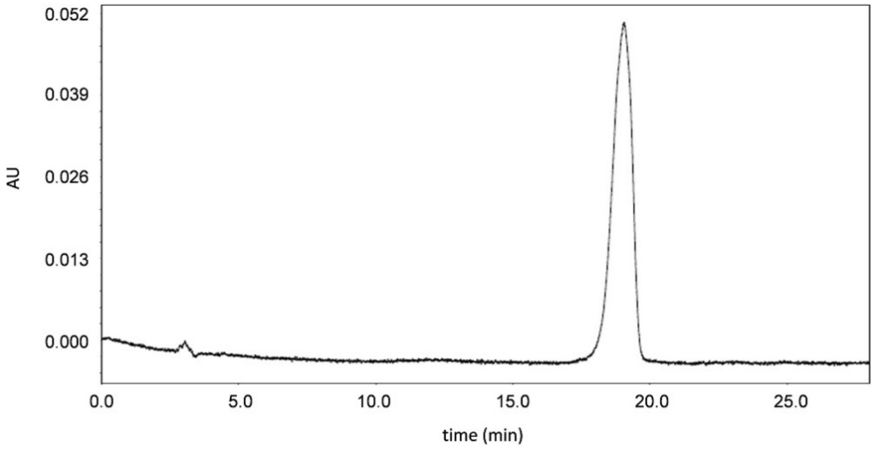

Figure S20. Analytical HPLC trace of H-VC-PAB-MMAE. Phenomenex Luna C18(2) column (5 μ, 250 mm x 4.6 mm) at a flow rate of 1 mL/min with gradient 68% solvent **A** / 32% solvent **B** to 57 % solvent **A** / 43% solvent **B** over 20 min (A = deionized water with 0.1% TFA, B = CH_3CN) detecting absorbance at 254 nm.

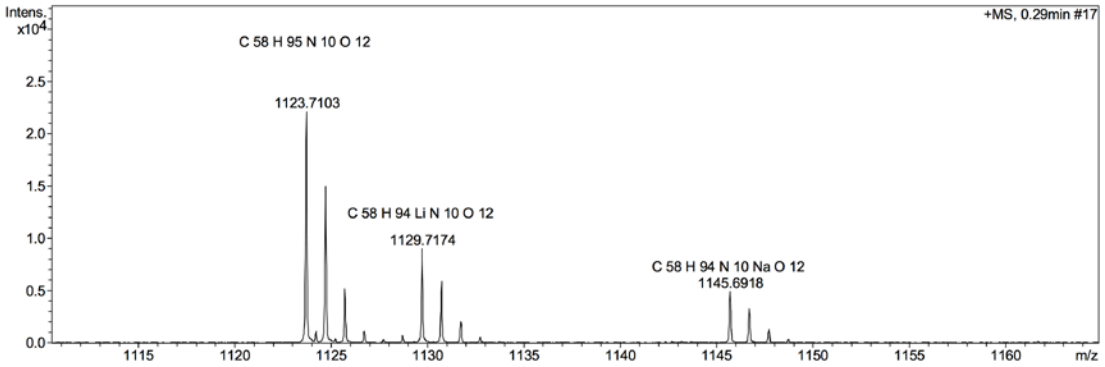

Figure S21. HRMS-ESI spectrum of H-VC-PAB-MMAE. $[M+Na]^+$ calcd. for $C_{58}H_{95}N_{10}NaO_{12}$, 1123.7125; found 1123.7103.

Synthesis of N_3-VC-PAB-MMAE: 3-(2-(2-(2-azidoethoxy)ethoxy)ethoxy) propanoic acid (10 mg, 0.039 mmol) was dissolved in dry DMF (250 μL) reacted with DIPEA (13.6 μL, 0.078 mmol) and HBTU (14 mg, 0.035 mmol). This solution was added to H-VC-PAB-MMAE dissolved in 250 μL of DMF. The reaction mixture was stirred 3 h at room temperature, then diluted with 2

mL of CH_3CN and directly purified by HPLC (see Figure bellow for details). Product containing fractions by mass (t_R=25.3) were lyophilized overnight to obtain compound **N_3-VC-PAB-MMAE** as a white solid (10 mg, 0.0074 mmol, 57% yield). HRMS: (m/z) $[M + Na]^+$ calcd. for $C_{67}H_{109}N_{13}NaO_{16}$, 1374.8007; found 1374.7974.

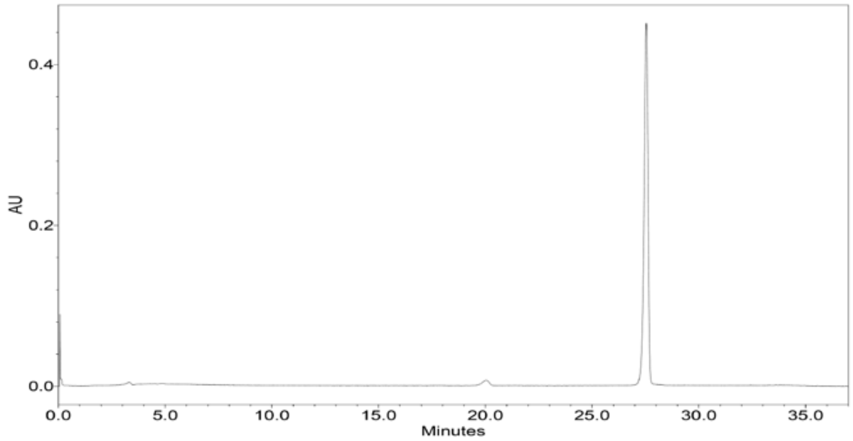

Figure S22. Analytical HPLC trace of N_3-VC-PAB-MMAE. Phenomenex Luna C18(2) column (5 µ, 250 mm x 4.6 mm) at a flow rate of 1 mL/min with gradient 68% solvent **A** / 32% solvent **B** to 57 % solvent **A** / 43% **B** over 20 min, then 57% solvent **A** to 32% solvent **A** / 68 % solvent **B** over 10 min (A = deionized water with 0.1% TFA, B = CH_3CN) detecting absorbance at 254 nm.

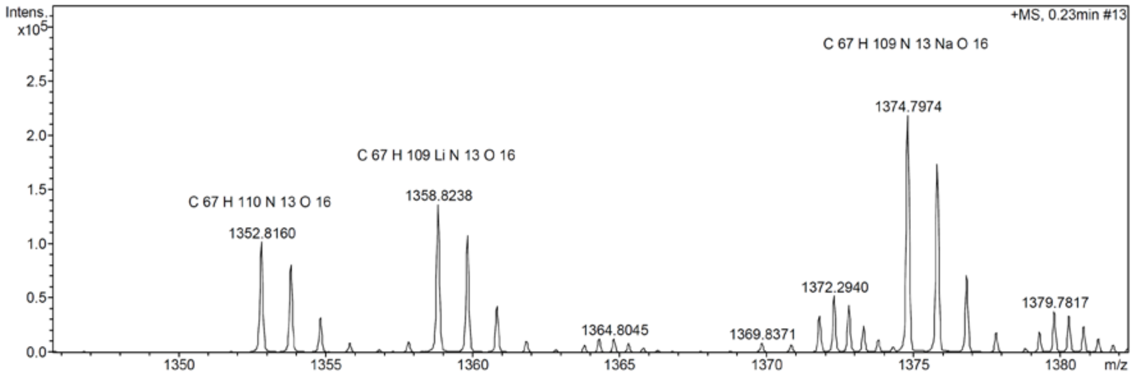

Figure S23. HRMS-ESI spectrum of N_3-VC-PAB-MMAE. $[M+Na]^+$ calcd. for $C_{67}H_{109}N_{13}NaO_{16}$, 1374.8007; found 1374.7974.

Synthesis of 5

To a solution of **N_3-VC-PAB-MMAE** (9.4 mg, 0.0069 mmol) and **1-(prop-2-yn-1-yl)-2-vinylpyridin-1-ium trifluoromethanesulfonate** (5.4 mg, 0.021 mmol) in a mixture DMF:H_2O 1:1 (200 µL), 22 µL of a 0.1 M solution of $CuSO_4$ in H_2O and 34 µL of a 0.1 M solution of Na ascorbate were added. The reaction was stirred for 6 hours at room temperature, then it was directly purified by HPLC (see Figure bellow for details). Product containing fraction (tR=16.2)

were collected and lyophilized overnight to obtain **4** as a white solid (0.8 mg, 0.5 μM, 8% yield). HRMS: (m/z) $[M]^+$ calcd. for $C_{77}H_{119}N_{14}O_{16}$, 1495.8923; found 1495.8883.

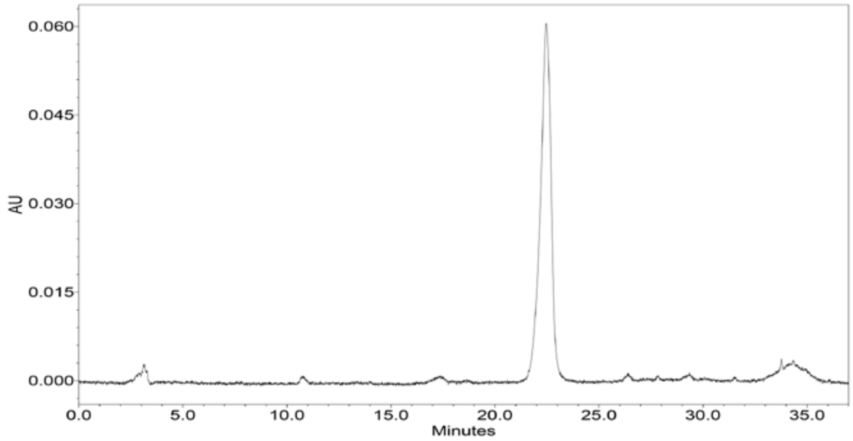

Figure S24. Analytical HPLC trace of 5. Phenomenex Luna C18(2) column (5 μ, 250 mm x 4.6 mm) at a flow rate of 1 mL/min with gradient 68% solvent **A** / 32% solvent **B** to 57 % solvent **A** / 43% **B** over 20 min, then 57% solvent **A** to 32% solvent **A** / 68 % solvent **B** over 10 min (A = deionized water with 0.1% TFA, B = CH_3CN) detecting absorbance at 254 nm.

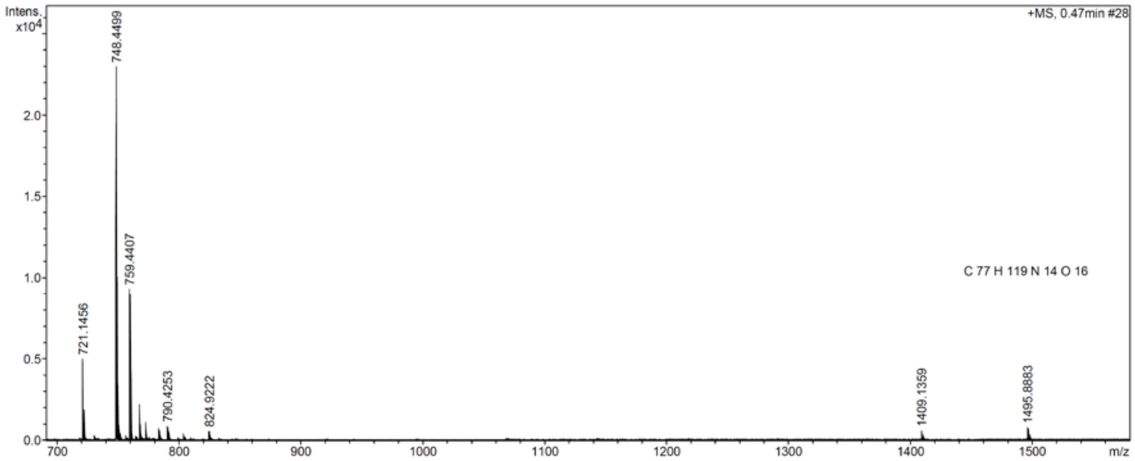

Figure S25. HRMS-ESI spectrum of 5. $[M]^+$ calcd. for $C_{77}H_{119}N_{14}O_{16}$, 1495.8923; found 1495.8883.

5. General Procedures for Protein Modification and Characterization

General procedure for protein and antibody conjugation with quaternized vinyl pyridine derivatives

To an eppendorf tube with NaP_i (50 mM or 20 mM, pH 8.0) and DMF (10% of total volume), an aliquot of a stock solution of protein (final concentration 10 μM) was added. Afterwards, a solution of the quaternized pyridine derivative (1 to 10 equiv.) in DMF was added and the resulting mixture was vortexed for 10 seconds. The reaction was mixed for 1 or 2 h at 37 ºC. A 10 μL aliquot of each reaction time was analysed by LC-MS and conversion to the expected product was observed.

LC-MS method for analysis of protein conjugation

LC-MS was performed on a Xevo G2-S TOF mass spectrometer coupled to an Acquity UPLC system using an Acquity Q6 UPLC BEH300 C4 column (1.7 mm, 2.1 × 50 mm). Solvents A, a water with 0.1% formic acid and B, 71% acetonitrile, 29% water and 0.075% formic acid were used as the mobile phase at a flow rate of 0.2 mL min^{-1}. The gradient was programmed as follows: 72% A to 100% B after 25 min then 100% B for 2 min and after that 72% A for 18 min. The electrospray source was operated with a capillary voltage of 2.0 kV and a cone voltage of 40 V. Nitrogen was used as the desolvation gas at a total flow of 850 L h^{-1}. Total mass spectra were reconstructed from the ion series using the MaxEnt algorithm preinstalled on MassLynx software (v. 4.1 from Waters) according to the manufacturer's instructions. To obtain the ion series described, the major peak(s) of the chromatogram were selected for integration and further analysis.

Stability of bioconjugates in human plasma

A 20 μL aliquot of the bioconjugate (10 μM) in NaP_i buffer (20 mM, pH 8.0) was thawed. 1 μL of reconstituted human plasma was added at room temperature and the resulting mixture vortexed for 10 seconds. The resulting reaction mixture was then mixed at 37 ºC. After 1 and 48 h, a 10 μL aliquot of each reaction mixture was analysed by LC-MS.

Stability of bioconjugates in the presence of GSH (1 mM)

A 20 μL aliquot of the bioconjugate (10 μM) in NaP_i buffer (20 mM, pH 8.0) was thawed. 1 μL of a 20 mM glutathione solution [6 mg glutathione dissolved in 1 mL of NaP_i buffer (20 mM, pH 8.0)] was added at room temperature and the resulting mixture vortexed for 10 seconds. The resulting reaction mixture was then mixed at 37 ºC. After 1 and 48 h, a 10 μL aliquot of each reaction mixture was analysed by LC-MS.

6. Ubiquitin-K63C Modification and Characterization

Ubiquitin-K63C (6 lysines, 1 free cysteine, no disulfides)

Sequence:

SAQIFVKTLT GKTITLEVEP SDTIENVKAK IQDKEGIPPD QQRLIFAGKQ LEDGRTLSDY NIQCESTLHL VLRLRGG

Isotopically Averaged Molecular Weight = 8566 Da

This mutant of ubiquitin was expressed and purified as previously reported.[8]

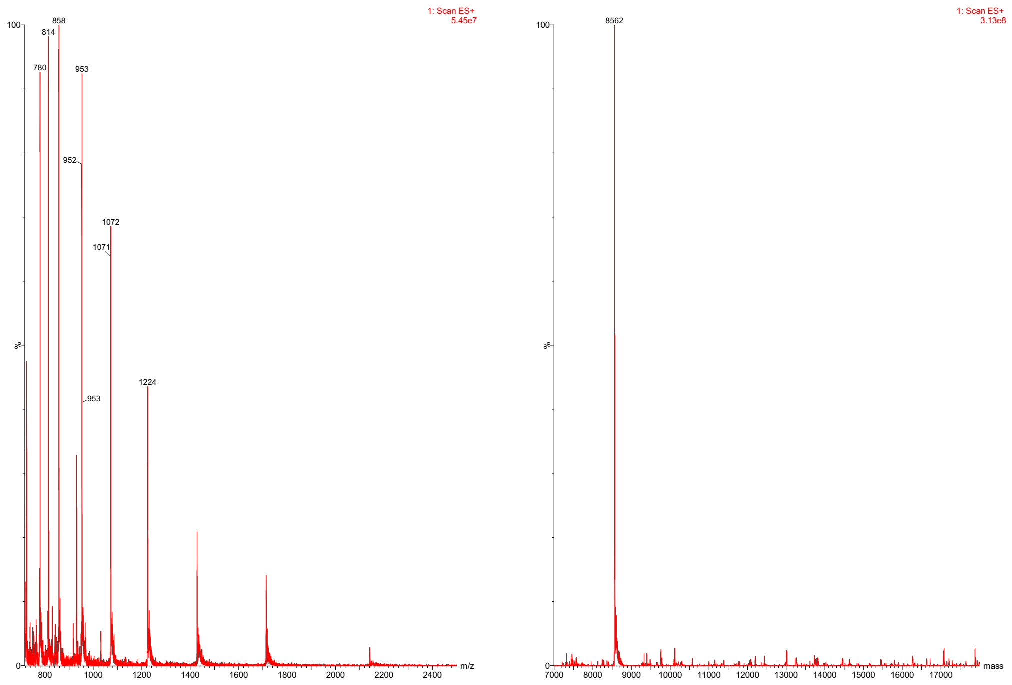

Figure S26. Combined ion series and deconvoluted mass spectrum of ubiquitin-K63C. Calcd. mass, 8566 Da, found: 8562 Da.

Reaction of ubiquitin-K63C with **1**

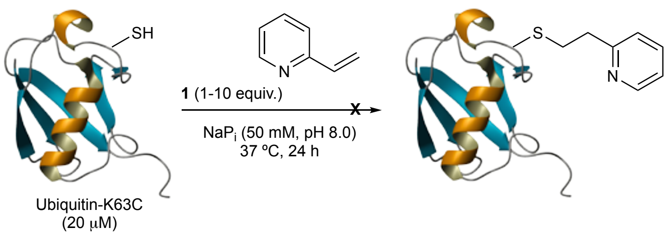

The reaction was performed according to the general procedure. To an eppendorf with 27 µL of NaP$_i$ (50 mM, pH 8.0) and 3.2 µL of DMF, was added an 8.8 µL aliquot of a stock solution of ubiquitin-K63C (90 µM) and the resulting mixture was vortexed for 10 seconds. Afterwards, a 0.95 mM solution of 2-vinylpyridine **1** (0.84 µL, 1 equiv.) in DMF was added and the reaction

mixed for 24 h at 37 ºC. At each reaction time, a 10 µL aliquot was analyzed by LC-MS and no conversion to the expected product was observed (calculated mass, 8669 Da; observed mass, 8567 Da). The same occurred when using 10 equiv. of **1** at 37 ºC for 24 h.

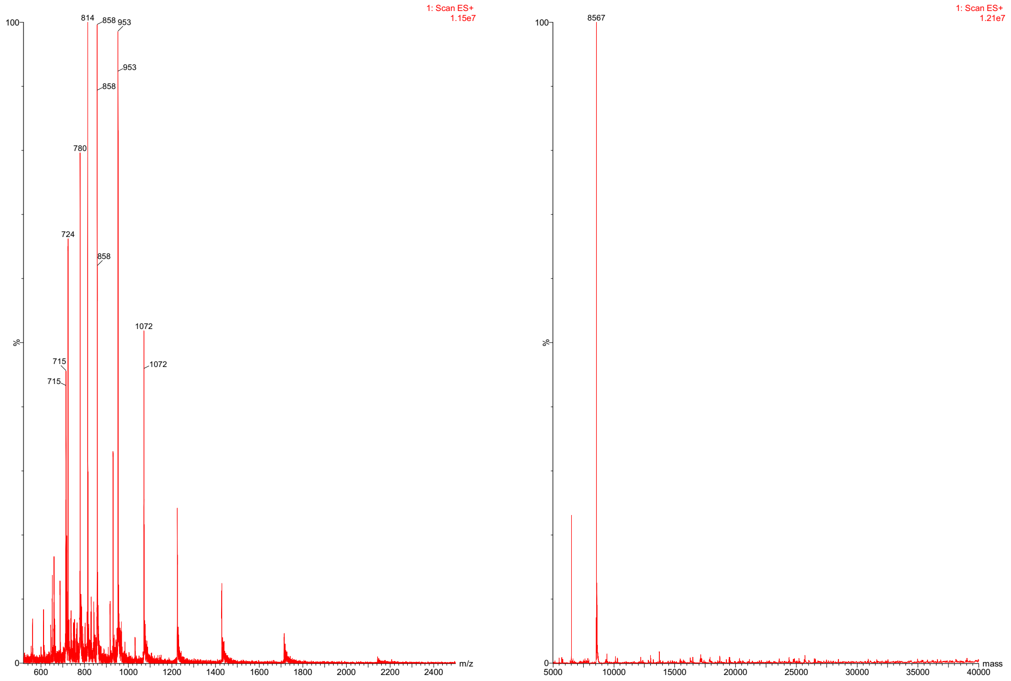

Figure S27. Combined ion series and deconvoluted mass spectra of the reaction of between ubiquitin-K63C (20 µM) with 1 equiv. of **1** after 24 h at 37 ºC.

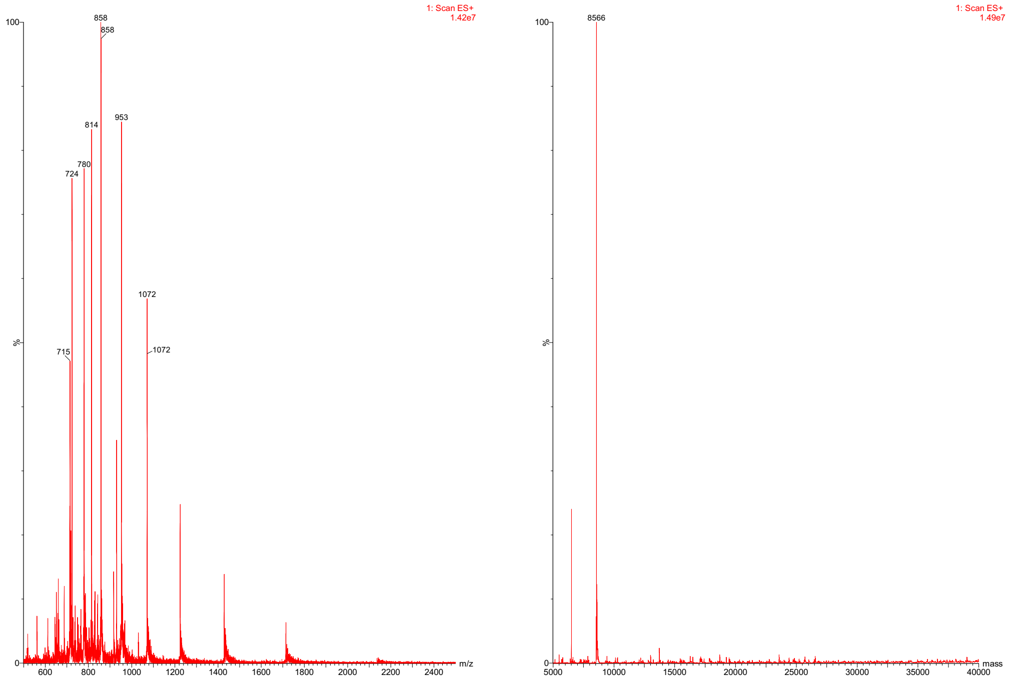

Figure S28. Combined ion series and deconvoluted mass spectra of the reaction of between ubiquitin-K63C (20 µM) with 10 equiv. of **1** after 24 h at 37 ºC.

Reaction of ubiquitin-K63C with **2**

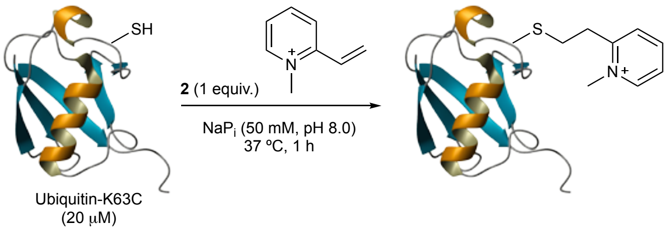

The reaction was performed according to the general procedure. To an eppendorf with 27 µL of NaP_i (50 mM, pH 8.0) and 3 µL of DMF, was added an 8.8 µL aliquot of a stock solution of ubiquitin-K63C (90 µM) and the resulting mixture was vortexed for 10 seconds. Afterwards, a 0.83 mM solution of 1-methyl-2-vinylpyridine **2** (0.96 µL, 1 equiv.) in DMF was added and the reaction mixed for 1 h at 37 ºC. At each reaction time, a 10 µL aliquot was analysed by LC-MS and conversion to the expected product was observed (calculated mass, 8684 Da; observed mass, 8685 Da). The same occurred when using 10 equiv. of **2** and leaving the reaction for 2 h, which shows the selectivity for cysteine over other nucleophilic residues even when the reagent is present in excess.

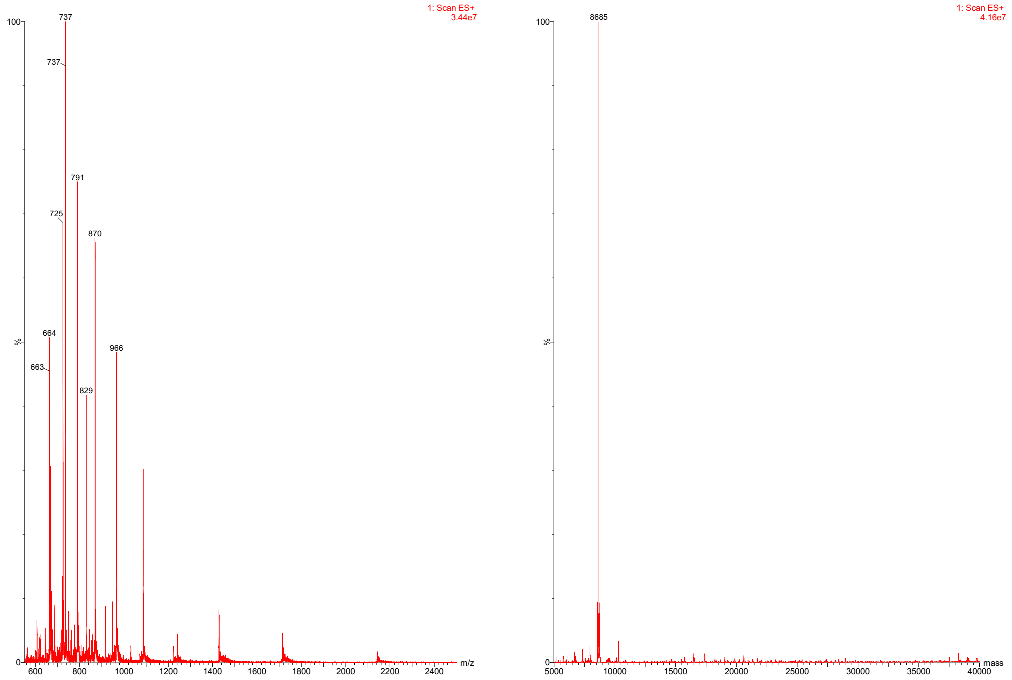

Figure S29. Combined ion series and deconvoluted mass spectra of the reaction of between ubiquitin-K63C (20 µM) with 1 equiv. of **2** after 1 h at 37 ºC.

7. Annexin V-315C Modification and Characterization

Annexin V-Cys315 (22 lysines, 1 free cysteine, no disulfides)

Sequence:

AQVLRGTVTD FPGFDERADA ETLRKAMKGL GTDEESILTL LTSRSNAQRQ 50
EISAAFKTLF GRDLLDDLKS ELTGKFEKLI VALMKPSRLY DAYELKHALK 100
GAGTNEKVLT EIIASRTPEE LRAIKQVYEE EYGSSLEDDV VGDTSGYYQR 150
MLVVLLQANR DPDAGIDEAQ VEQDAQALFQ AGELKWGTDE EKFITIFGTR 200
SVSHLRKVFD KYMTISGFQI EETIDRETSG NLEQLLLAVV KSIRSIPAYL 250
AETLYYAMKG AGTDDHTLIR VMVSRSEIDL FNIRKEFRKN FATSLYSMIK 300
GDTSGDYKKA LLLL**C**GEDD 319

Isotopically Averaged Molecular Weight = 35805 Da

This mutant of ubiquitin was expressed and purified as previously reported.[9]

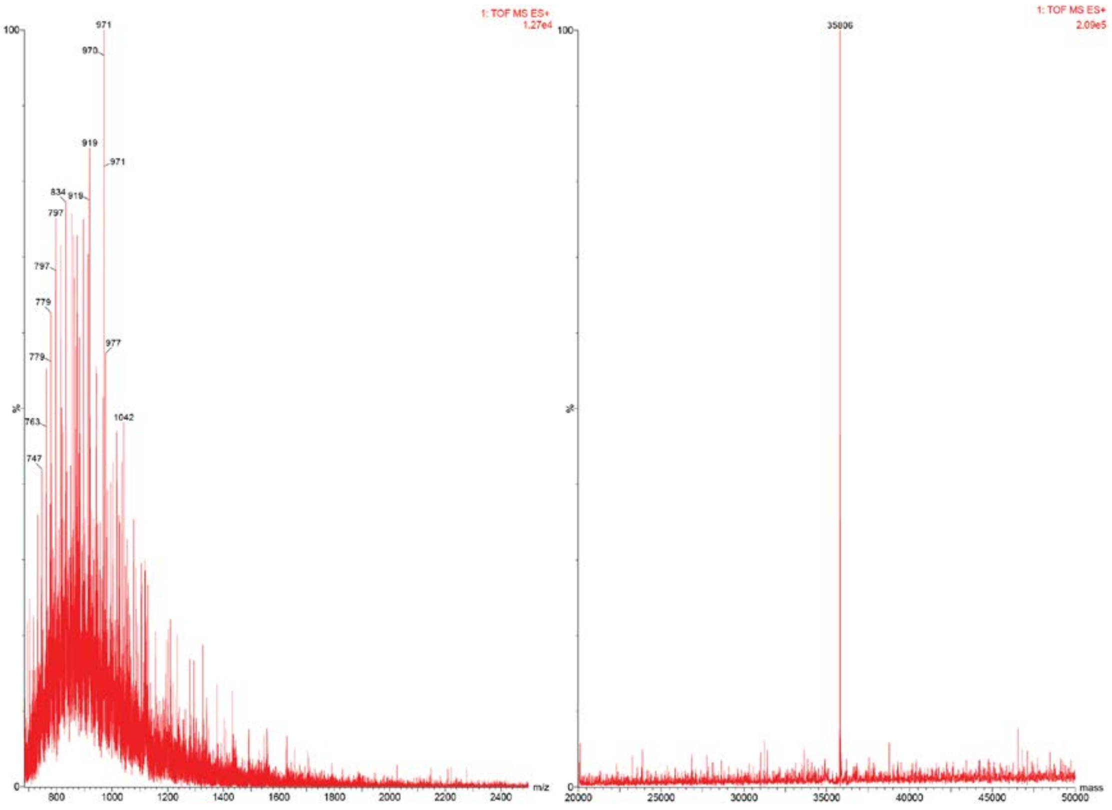

Figure S30. Combined ion series and deconvoluted mass spectrum of Annexin V-Cys315. Calcd. mass, 35805 Da, found: 35806 Da.

Reaction of Annexin V-Cys315 with **2**

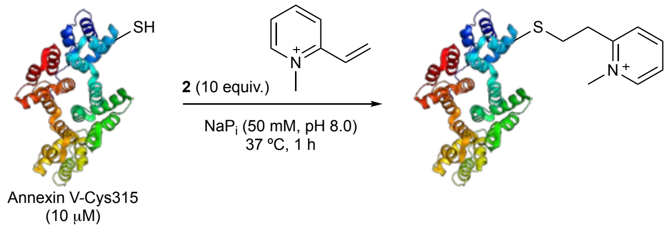

The reaction was performed according to the general procedure. To an eppendorf with 22 µL of NaP_i (50 mM, pH 8.0) and 3.1 µL of DMF, was added a 14 µL aliquot of a stock solution of Annexin V-Cys315 (27.9 µM) and the resulting mixture was vortexed for 10 seconds. Afterwards, a 8.3 mM solution of 1-methyl 2-vinylpyridine **2** (0.96 µL, 10 equiv.) in DMF was added and the reaction mixed for 1 h at 37 ºC. At each reaction time, a 10 µL aliquot was analysed by LC-MS and conversion to the expected product was observed (calculated mass, 35923 Da; observed mass, 35924 Da).

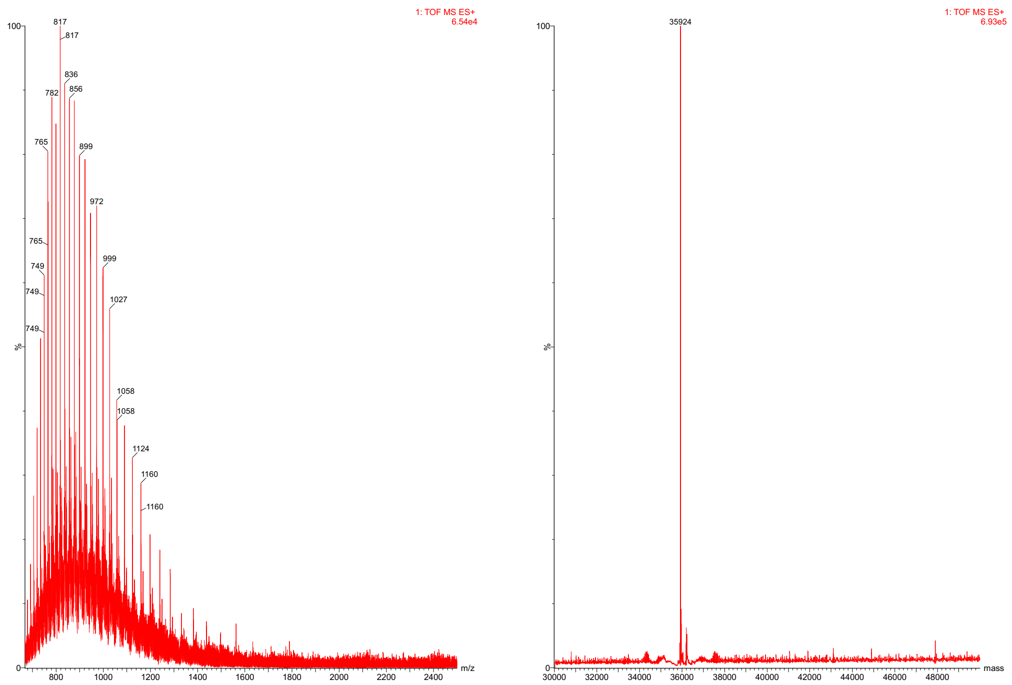

Figure S31. Combined ion series and deconvoluted mass spectra of the reaction of between annexin V-Cys315 (20 µM) with 10 equiv. of **2** after 1 h at 37 ºC.

Optimisation of reaction conditions for Annexin V-Cys315 with 2

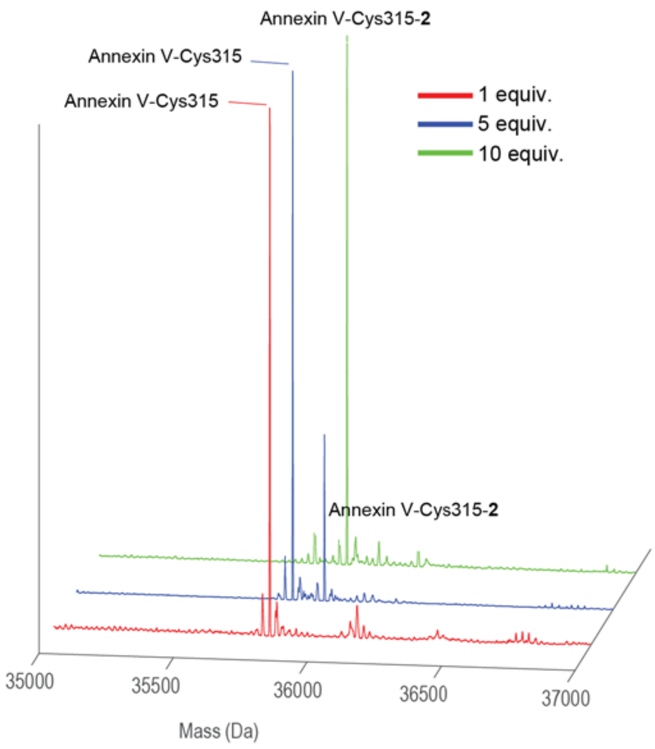

Figure S32. Overlaid electrospray MS spectra of Annexin V-Cys315 incubated with 1, 5 or 10 equivalents of **2** for 1 h at 37 ºC. Expected mass of 35923 and observed mass of 35924 for Annexin V-Cys315-**2**. Combined ion series and mass spectrum after deconvolution using MaxEnt algorithm.

8. C2Am-Cys95 Modification and Characterization

C2Am-Cys95 (14 lysines, 1 free cysteine, no disulfides)

Sequence:

GSPGISGGGG GILDSMVEKL GKLQYSLDYD FQNNQLLVGI IQAAELPALD 50

MGGTSDPYVK VFLLPDKKKK FETKVHRKTL NPVFNEQFTF KVPY<u>**C**</u>ELGGK 100

TLVMAVYDFD RFSKHDIIGE FKVPMNTVDF GHVTEEWRDL QSAEK 145

Isotopically Averaged Molecular Weight = 16222 Da

This mutant of C2Am was expressed and purified as previously reported.[10]

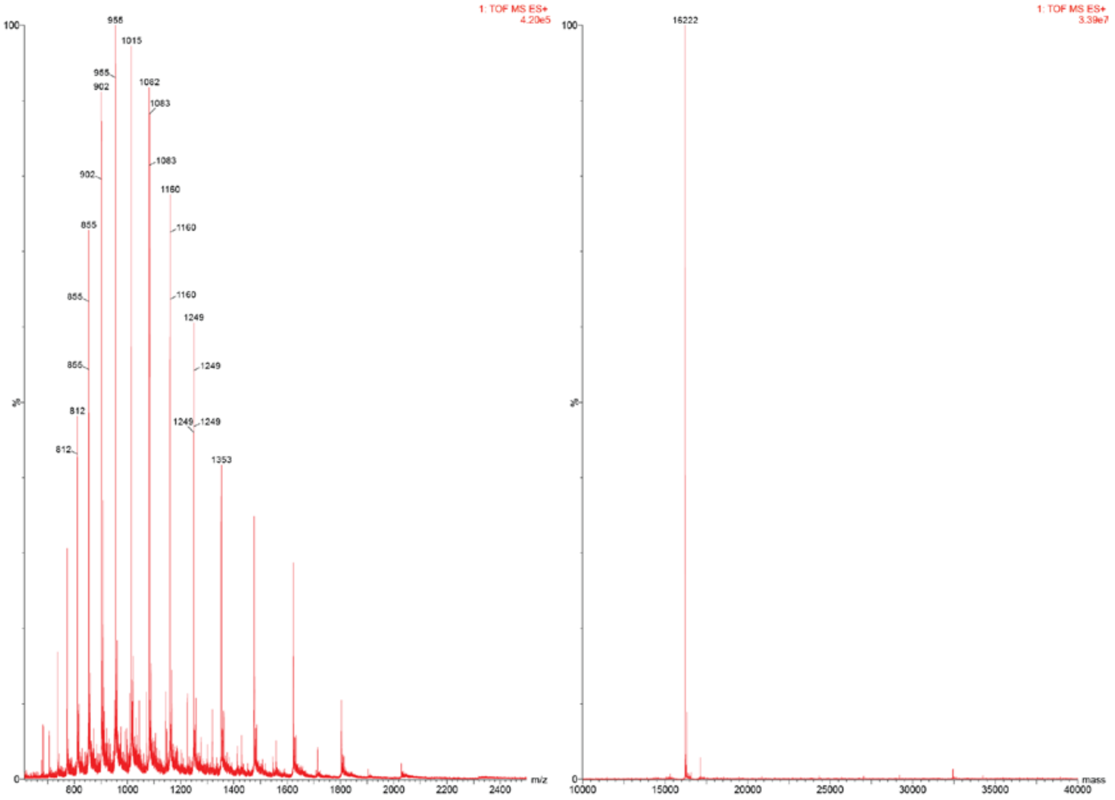

Figure S33. Combined ion series and deconvoluted mass spectra of C2Am-Cys95. Calcd. mass, 16222 Da, found: 16222 Da.

Reaction of C2Am-Cys95 with **1**

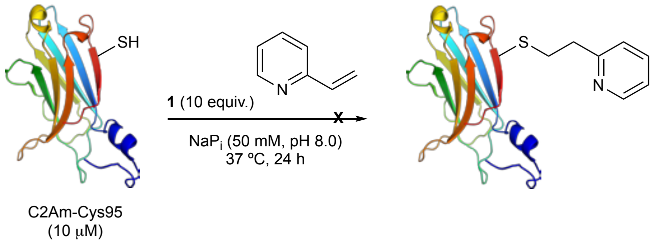

The reaction was performed according to the general procedure. To an eppendorf with 30 μL of NaPi (50 mM, pH 8.0) and 3.2 μL of DMF, was added a 6.5 μL aliquot of a stock solution of C2Am-Cys95 (61.5 μM) and the resulting mixture was vortexed for 10 seconds. Afterwards, a 9.5 mM solution of 2-vinylpyridine **1** (0.84 μL, 10 equiv.) in DMF was added and the reaction mixed for 24 h at 37 ºC. At each reaction time, a 10 μL aliquot was analysed by LC-MS and no conversion to the expected product was observed (calculated mass, 16325 Da; observed mass, 16222 Da).

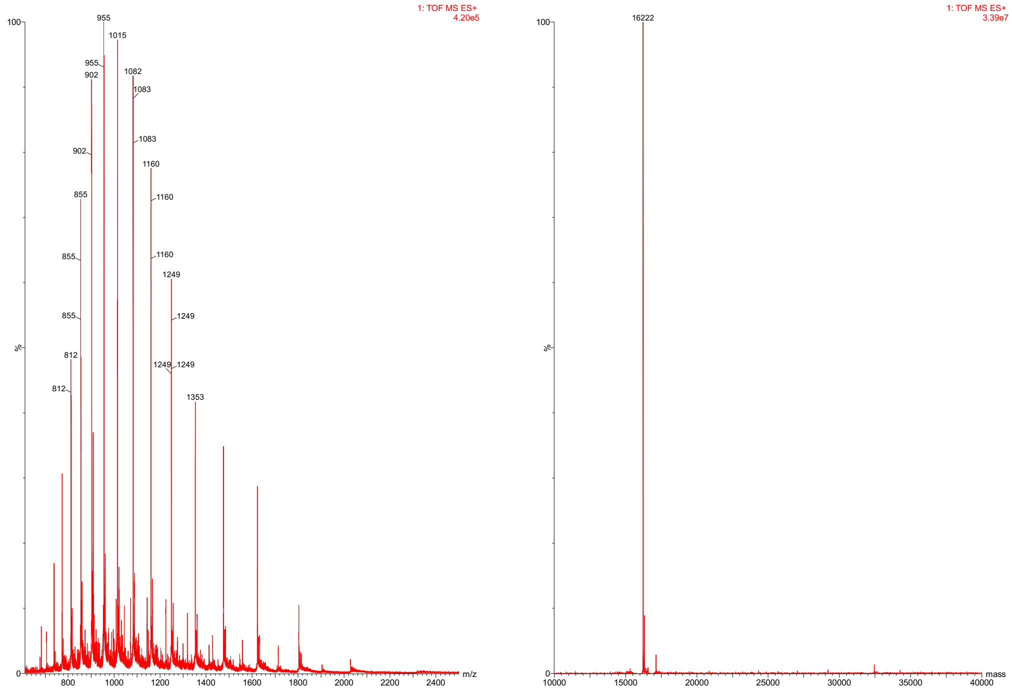

Figure S34. Combined ion series and deconvoluted mass spectra of the reaction of between C2Am-Cys95 (10 μM) with 10 equiv. of **1** after 24 h at 37 ºC.

Reaction of C2Am-Cys95 with **2**

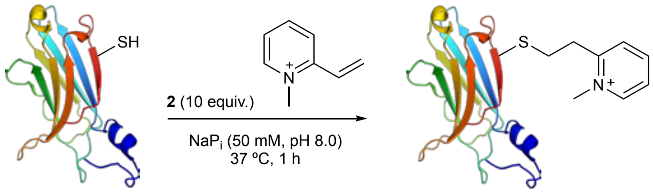

C2Am-Cys95
(10 μM)

The reaction was performed according to the general procedure. To an eppendorf with 30 μL of NaP$_i$ (50 mM, pH 8.0) and 3.2 μL of DMF, was added a 6.5 μL aliquot of a stock solution of C2Am (61.5 μM) and the resulting mixture was vortexed for 10 seconds. Afterwards, a 8.3 mM solution of 1-methyl 2-vinylpyridine **2** (0.96 μL, 10 equiv.) in DMF was added and the reaction mixed for 1 h at 37 ºC. At each reaction time, a 10 μL aliquot was analysed by LC-MS and conversion to the expected product was observed (calculated mass, 16340 Da; observed mass, 16339 Da). Under identical conditions but at pH 7.0, the reaction proceeded in the same manner.

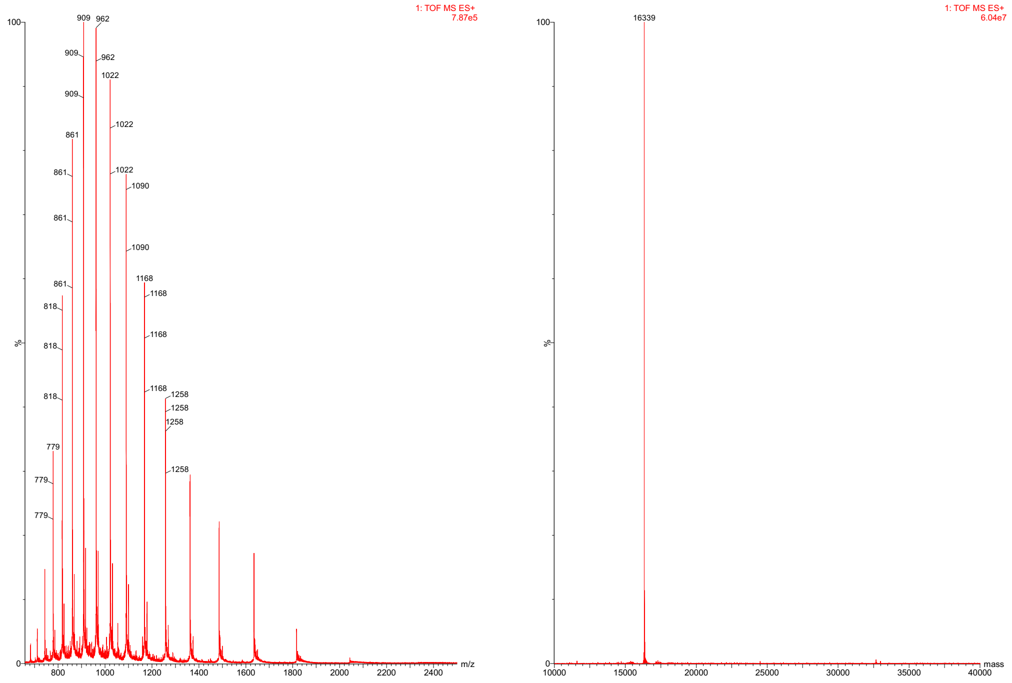

Figure S35. Combined ion series and deconvoluted mass spectra of the reaction of between C2Am-Cys95 (10 μM) with 10 equiv. of **2** after 1 h at 37 ºC in NaP$_i$ (50 mM, pH 8.0).

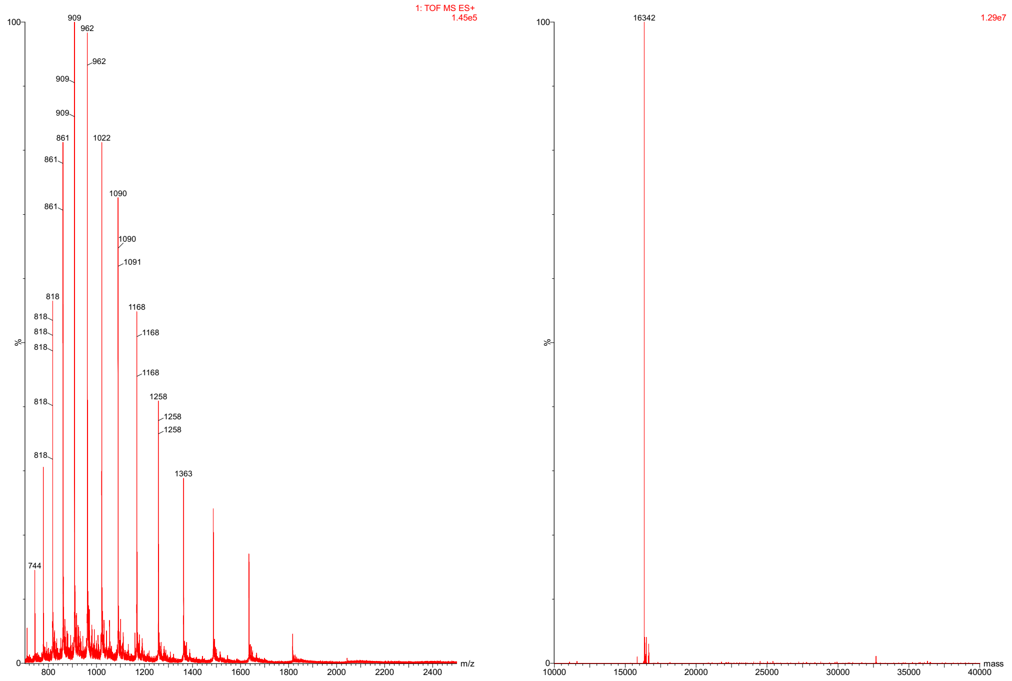

Figure S36. Combined ion series and deconvoluted mass spectra of the reaction of between C2Am-Cys95 (10 µM) with 10 equiv. of **2** after 1 h at 37 ºC in NaP_i (50 mM, pH 7.0).

<u>Control:</u> Reaction of C2Am-Cys95-**2** with 5,5′-dithiobis(2-nitrobenzoic acid)

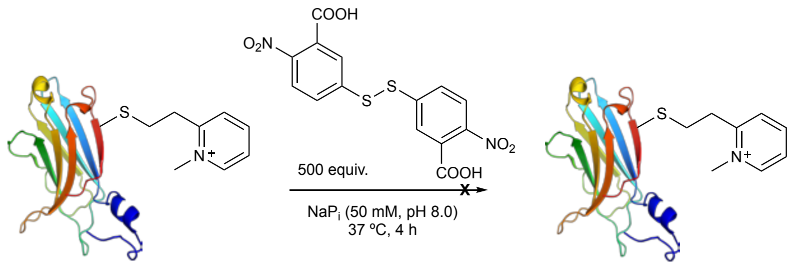

A 40 µL aliquot of C2Am-Cys95-**2** (10 µM) was transferred to a 0.5 mL eppendorf tube. An aliquot of 0.8 µL (500 equiv.) of a stock suspension of 5,5′-dithiobis(2-nitrobenzoic acid) (0.500 mM) was added and the resulting mixture vortexed for 10 seconds. After 4 h of additional mixing at 37 ºC, small molecules were removed from the reaction mixture by loading the sample into a Zeba Spin Desalting Column previously equilibrated with NaP_i (50 mM, pH 8.0). The sample was eluted via centrifugation (2 min, 1000xg). A 10 µL aliquot was analysed by LC-MS and no conversion to a potentially doubly modified protein was observed (calculated mass, 16537 Da; observed mass, 16341 Da).

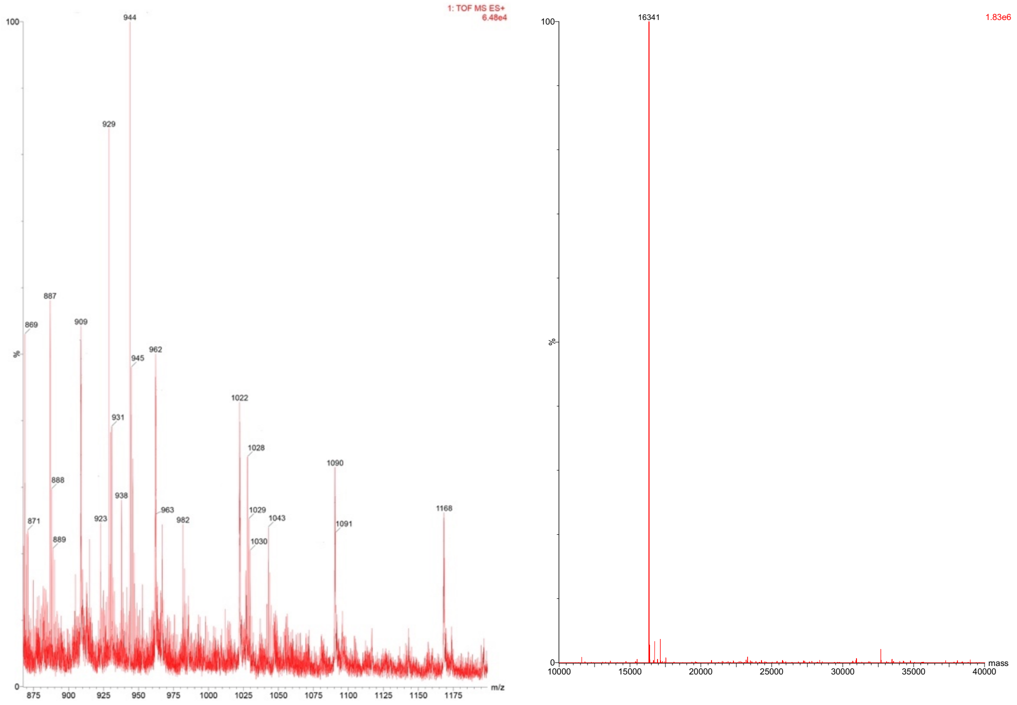

Figure S37. Combined ion series and deconvoluted mass spectra of the reaction of between C2Am-Cys95-**2** with 500 equiv. of 5,5′-dithiobis(2-nitrobenzoic acid) after 4 h at 37 ºC.

<u>Control:</u> Reaction of C2Am-Cys95-Ellman's with **2**

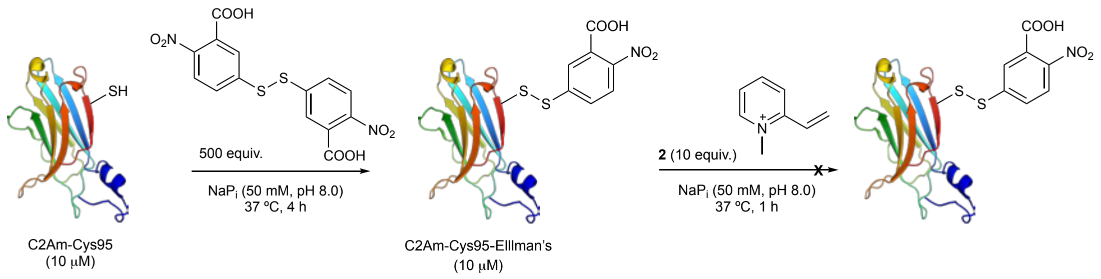

A 40 µL aliquot of C2Am-Cys95-Ellman's (10 µM) was transferred to a 0.5 mL eppendorf tube. An aliquot of 1.0 µL (10 equiv.) of a stock solution of **2** (8.3 mM) was added and the resulting mixture vortexed for 10 seconds. After 1 h of additional mixing, at 37 ºC, a 10 µL aliquot was analysed by LC-MS and no conversion to the potentially doubly modified protein was observed (calculated mass, 16537 Da; observed mass, 16421 Da).

1st Step – Reaction of C2Am-Cys95 with with 5,5′-dithiobis(2-nitrobenzoic acid)

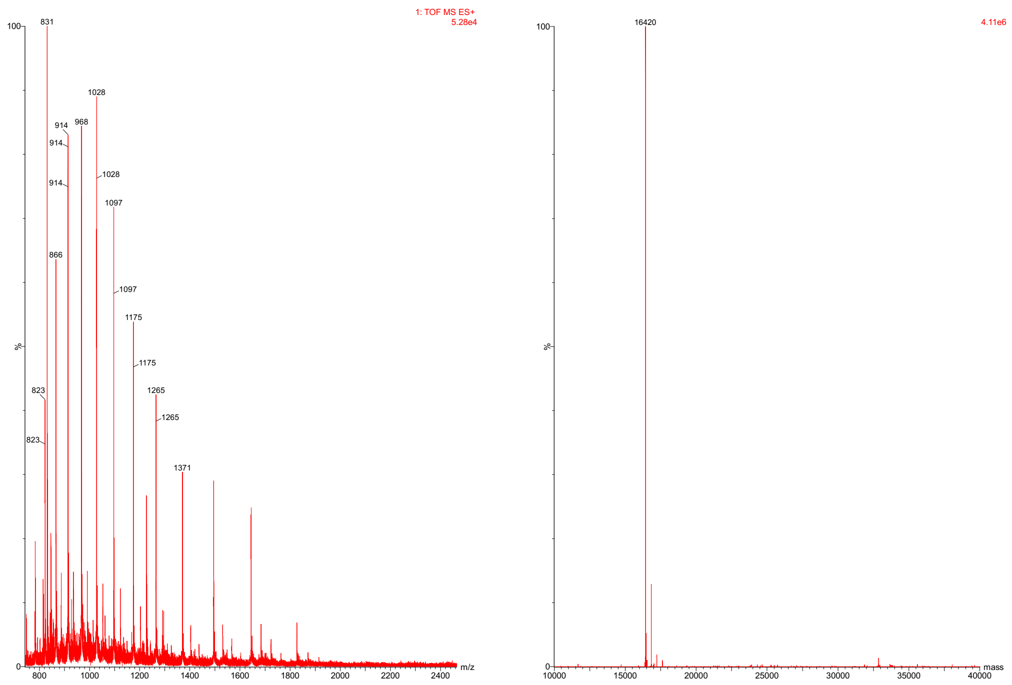

Figure S38. Combined ion series and deconvoluted mass spectra of the reaction of between C2Am-Cys95 with 500 equiv. of 5,5′-dithiobis(2-nitrobenzoic acid) after 4 h at 37 ºC.

2nd Step – Reaction of C2Am-Cys95-Ellman's with **2**

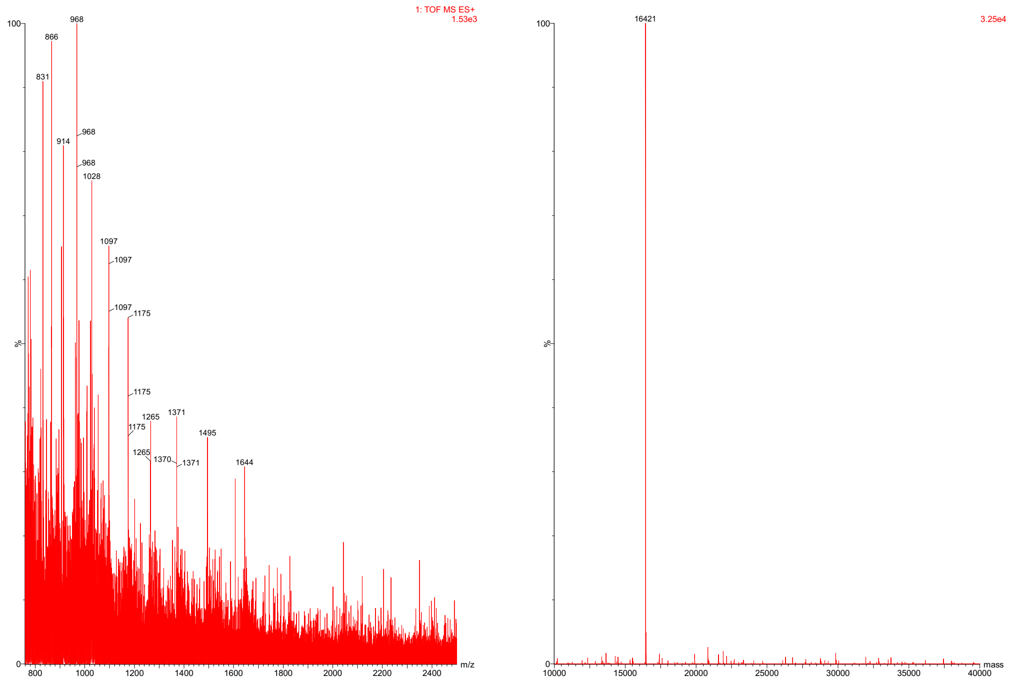

Figure S39. Combined ion series and deconvoluted mass spectra of the reaction of between C2Am-Cys95-Ellman's with 10 equiv. of **2** after 1 h at 37 ºC.

Stability studies for C2Am-Cys95-2 conjugate – pH

A 40 µL aliquot of C2Am-**2** (10 µM) was transferred to a 0.5 mL eppendorf tube. A buffer exchange column Viva 500 (10 KDa) was use to change the buffer to pH 5.0 to pH 8.0. The samples were eluted via centrifugation (5 min, 1000xg) using 50 mM sodium phosphate buffer at the desired pH. 10 µL of this diluted sample was injected after 24, 48 and 168 hours (calculated mass, 16340 Da; observed mass, 16341 Da).

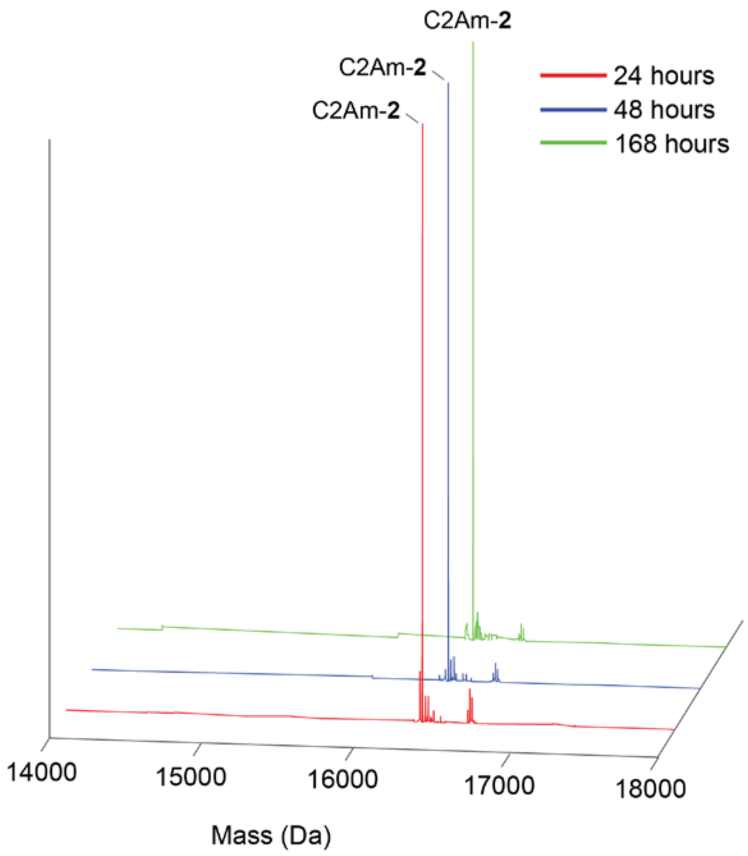

Figure S40. Overlaid electrospray MS spectra of C2Am-**2** in NaP_i (50 mM) at pH 5.0. Expected mass is 16340 Da and observed mass is 16341 Da. Combined ion series and mass spectra after deconvolution using MaxEnt algorithm.

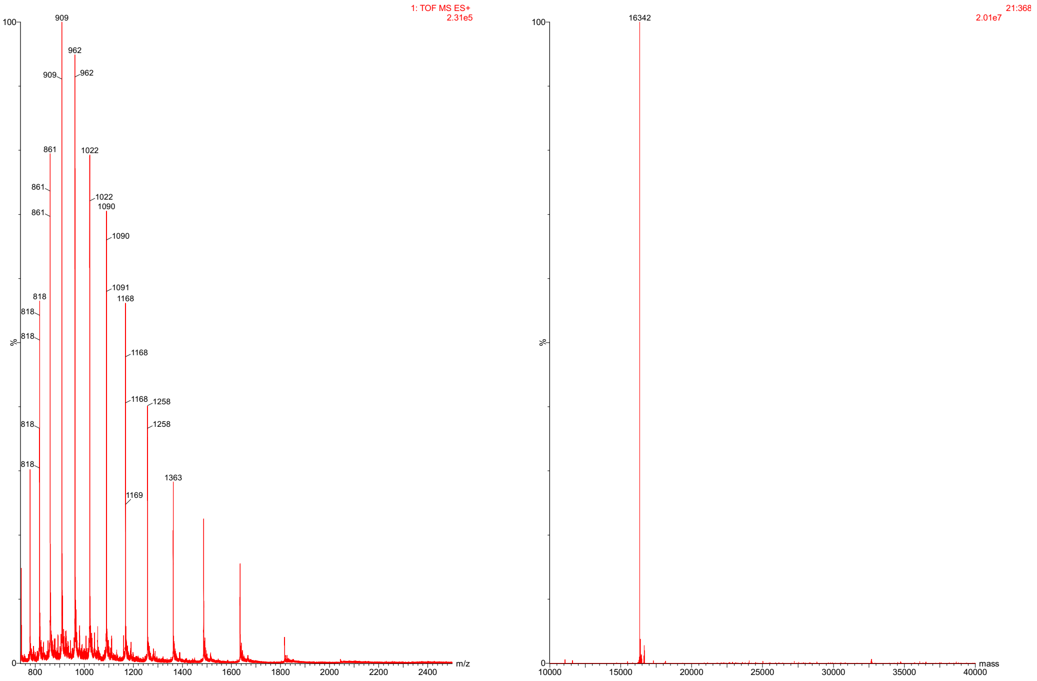

Figure S41. Combined ion series and deconvoluted mass spectra of the stability of C2Am-**2** after 24 h in pH 5.0 at 37 ºC.

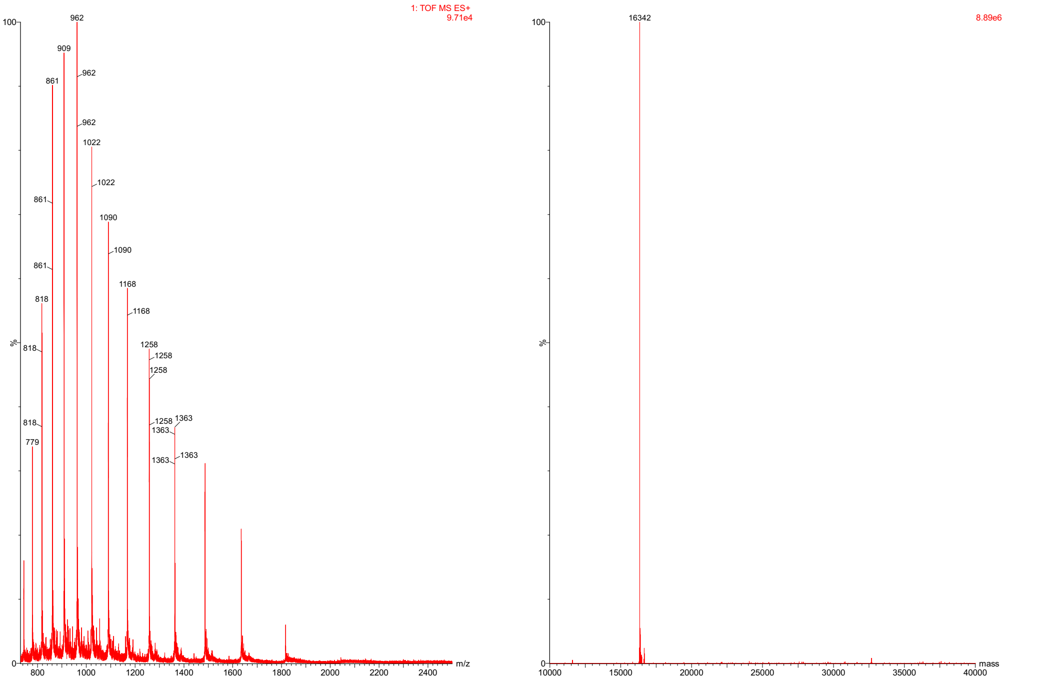

Figure S42. Combined ion series and deconvoluted mass spectra of the stability of C2Am-**2** after 48 h in pH 5.0 at 37 ºC.

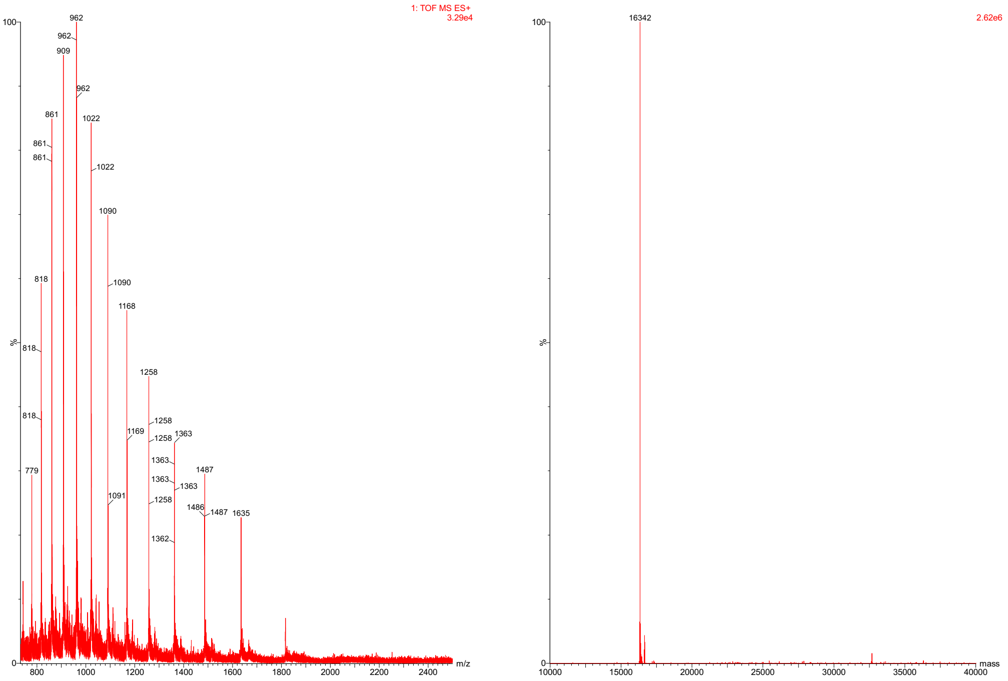

Figure S43. Combined ion series and deconvoluted mass spectra of the stability of C2Am-**2** after 168 h in pH 5.0 at 37 ºC.

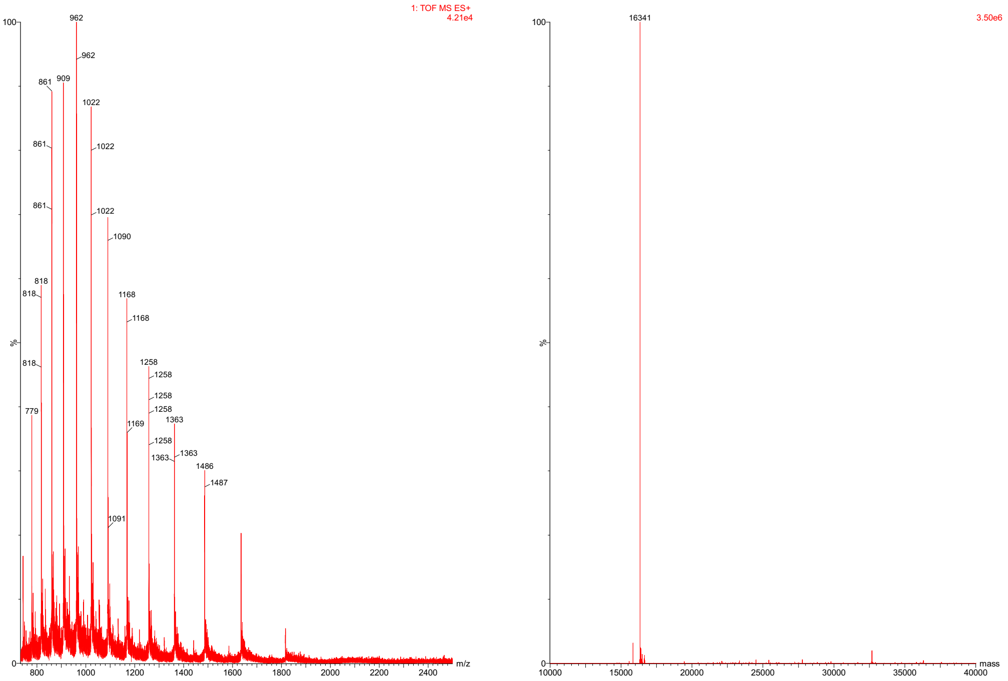

Figure S44. Combined ion series and deconvoluted mass spectra of the stability of C2Am-**2** after 48 h in pH 8.0 at 37 ºC.

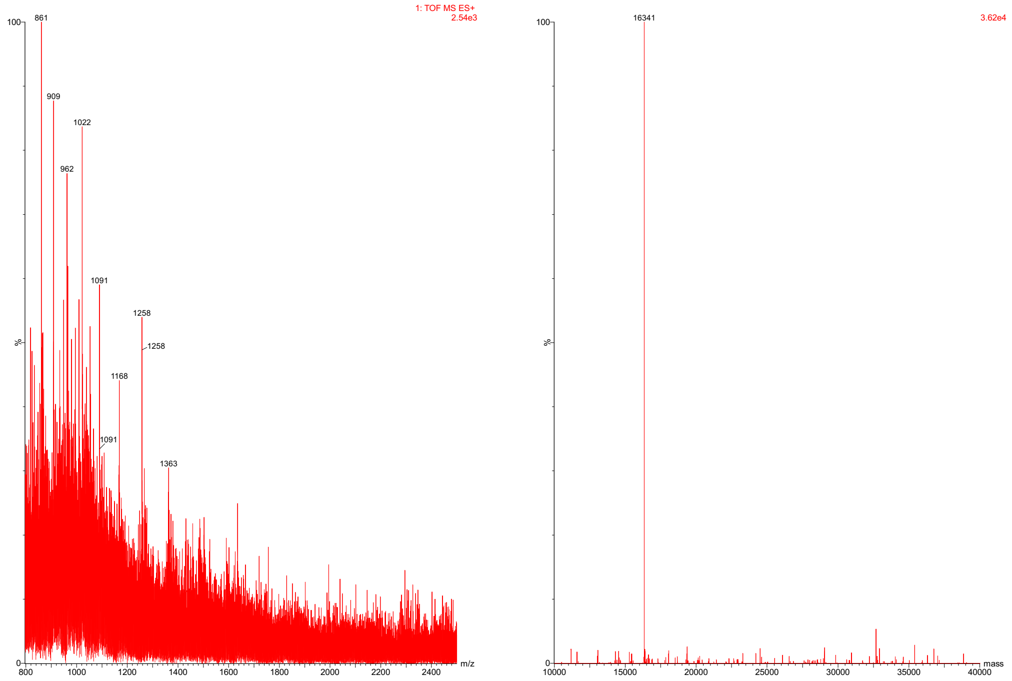

Figure S45. Combined ion series and deconvoluted mass spectra of the stability of C2Am-**2** after 168 h in pH 8.0 at 37 ºC.

Stability studies for C2Am-2 conjugate in plasma

A 20 µL aliquot of C2Am-**2** (10 µM) in 50 mM NaP_i buffer at pH 8.0 was thawed. 1 µL of reconstituted human plasma (Sigma-Aldrich) was added at room temperature and the resulting mixture vortexed for 10 seconds. The resulting reaction mixture was then mixed at 37 °C. After 1, 24 and 48 h, a 10 µL aliquot of each reaction mixture was analysed by LC-MS. No significant degradation of the adduct was observed at either time point.

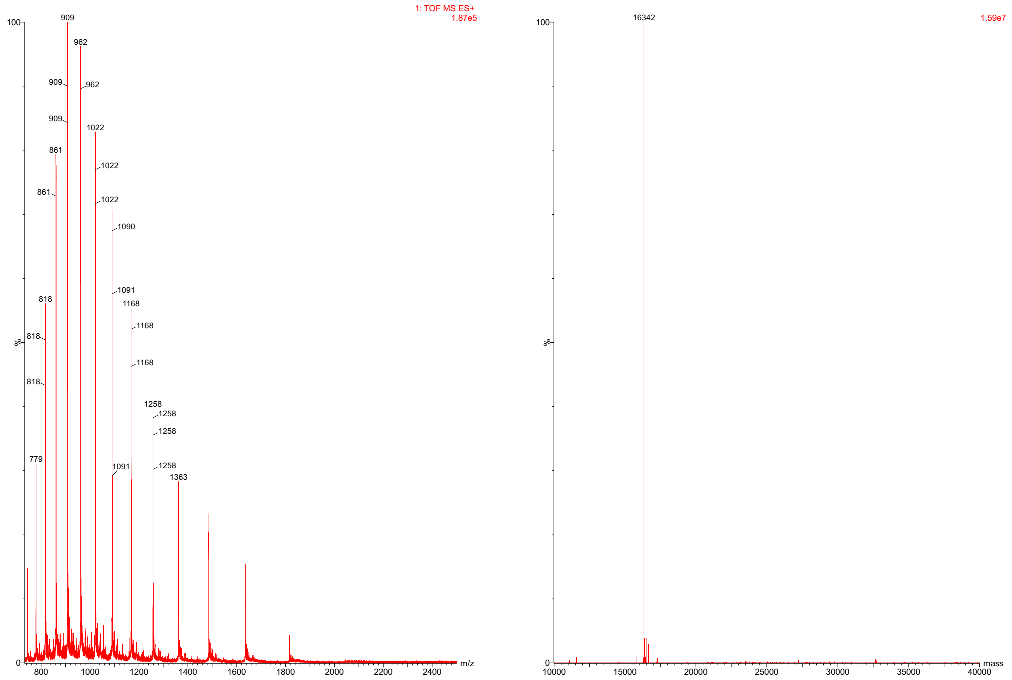

Figure S46. Combined ion series and deconvoluted mass spectra of C2Am-**2** after incubation in human plasma for 48 h at 37 ºC.

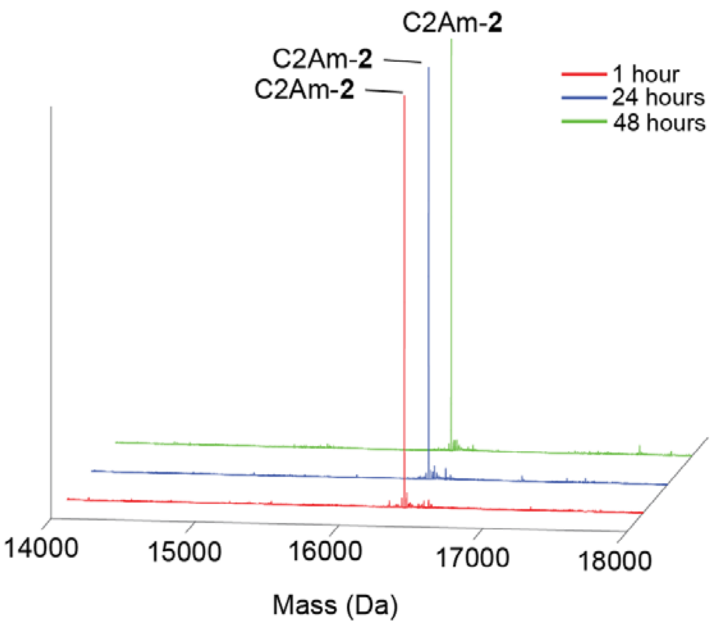

Figure S47. Overlaid electrospray MS spectra of C2Am-**2** in NaP_i (50 mM) in human plasma at different times. Expected mass is 16340 Da and observed mass is 16341 Da. Combined ion series and mass spectra after deconvolution using MaxEnt algorithm.

9. rHSA-Cys34 Modification and Characterization

rHSA (59 lysine, 1 free cysteine, 17 disulfides)

Sequence:

DAHKSEVAHR FKDLGEENFK ALVLIAFAQY LQQCPFEDHV KLVNEVTEFA 50
KTCVADESAE NCDKSLHTLF GDKLCTVATL RETYGEMADC CAKQEPERNE 100
CFLQHKDDNP NLPRLVRPEV DVMCTAFHDN EETFLKKYLY EIARRHPYFY 150
APELLFFAKR YKAAFTECCQ AADKAACLLP KLDELRDEGK ASSAKQRLKC 200
ASLQKFGERA FKAWAVARLS QRFPKAEFAE VSKLVTDLTK VHTECCHGDL 250
LECADDRADL AKYICENQDS ISSKLKECCE KPLLEKSHCI AEVENDEMPA 300
DLPSLAADFV ESKDVCKNYA EAKDVFLGMF LYEYARRHPD YSVVLLLRLA 350
KTYETTLEKC CAAADPHECY AKVFDEFKPL VEEPQNLIKQ NCELFEQLGE 400
YKFQNALLVR YTKKVPQVST PTLVEVSRNL GKVGSKCCKH PEAKRMPCAE 450
DYLSVVLNQL CVLHEKTPVS DRVTKCCTES LVNRRPCFSA LEVDETYVPK 500
EFNAETFTFH ADICTLSEKE RQIKKQTALV ELVKHKPKAT KEQLKAVMDD 550
FAAFVEKCCK ADDKETCFAE EGKKLVAASQ AALGL 585

Isotopically Averaged Molecular Weight = 66472 Da; with 17 internal disulfides: 66439 Da

This protein was kindly provided by Albumedix Ltd.

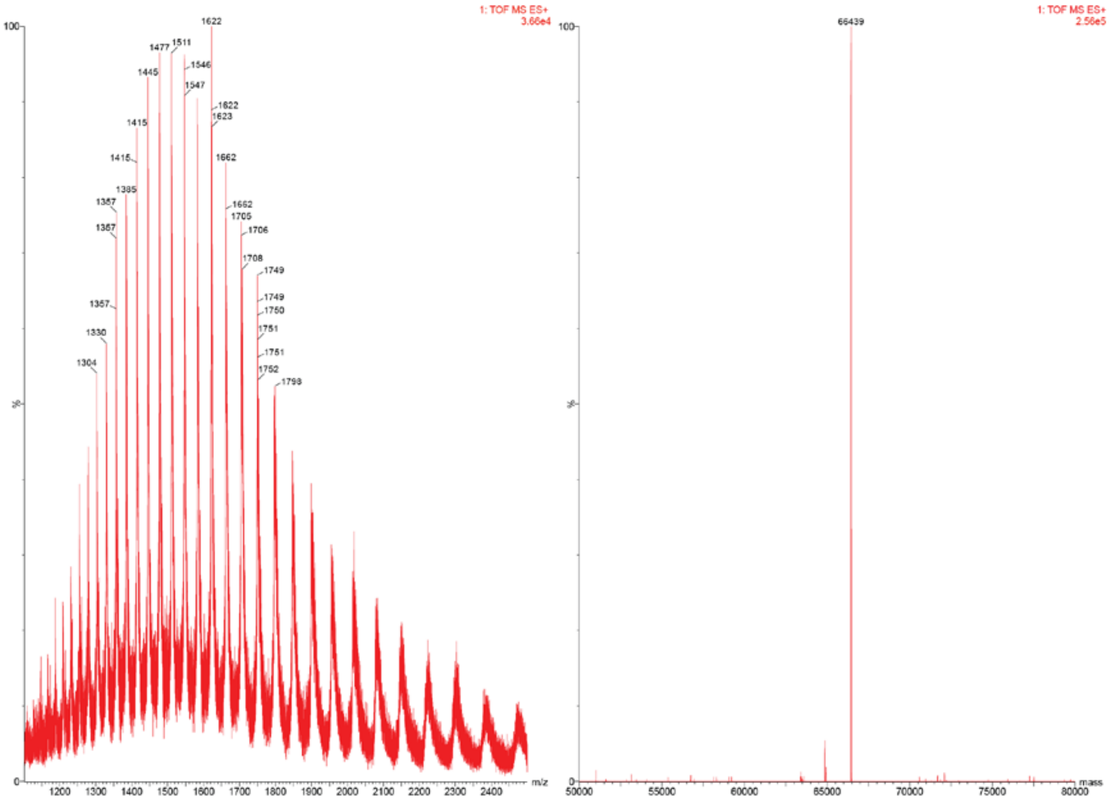

Figure S48. Combined ion series and deconvoluted mass spectra of rHSA-Cys34.

Reaction of rHSA with **2** at pH 8.0

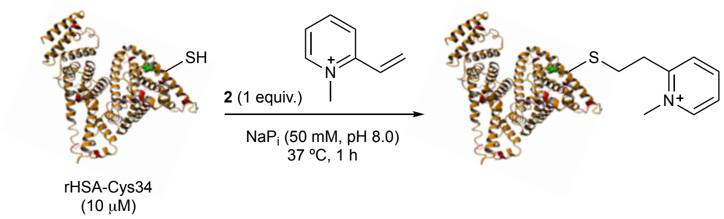

The reaction was performed according to the general procedure. To an eppendorf with 9.4 μL of NaP$_i$ (50 mM, pH 8.0) and 3 μL of DMF, was added a 27 μL aliquot of a stock solution of rHSA (15.05 μM) and the resulting mixture was vortexed for 10 seconds. Afterwards, a 0.83 mM solution of **2** (1 μL, 1 equiv.) in DMF was added and the reaction mixed for 1 h at 37 ºC. A 10 μL aliquot was analysed by LC-MS and conversion to the expected product was observed (calculated mass, 66557 Da; observed mass, 66555 Da). The same occurred when using 10 equiv. of **2** and leaving the reaction for 2 h.

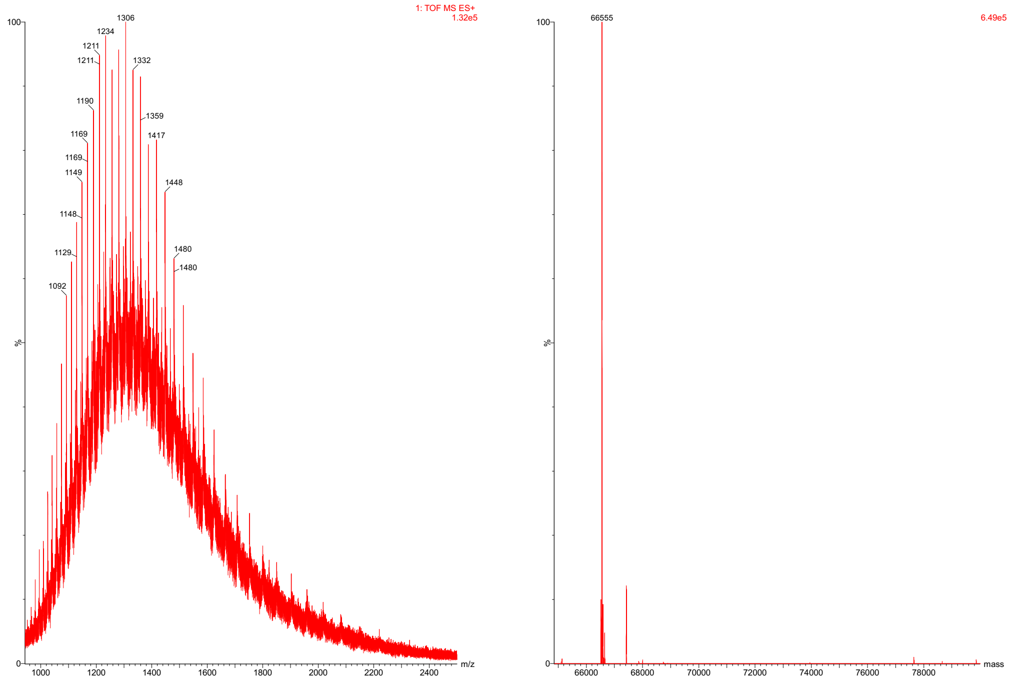

Figure S49. Combined ion series and deconvoluted mass spectra of the reaction of between rHSA-Cys34 (10 μM) with 1 equiv. of **2** after 1 h at 37 ºC.

Control: Reaction of rHSA with **1** or **2** at pH 5.5

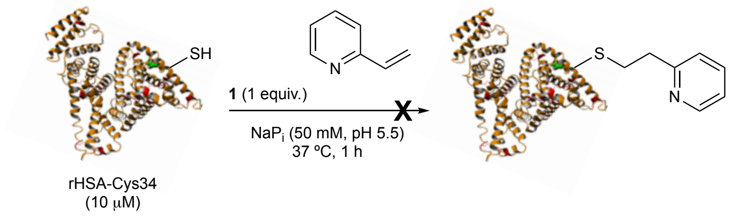

The reaction was performed according to the general procedure. To an eppendorf with 9.4 μL of NaPi (50 mM, pH 5.5) and 3.42 μL of DMF, was added a 27 μL aliquot of a stock solution of rHSA (15.05 μM) and the resulting mixture was vortexed for 10 seconds. Afterwards, a 1.04 mM solution of **1** (0.58 μL, 1 equiv.) in DMF was added and the reaction mixed for 1 h at 37 ºC. A 10 μL aliquot was analysed by LC-MS, and no conversion of the starting protein to a product was detected.

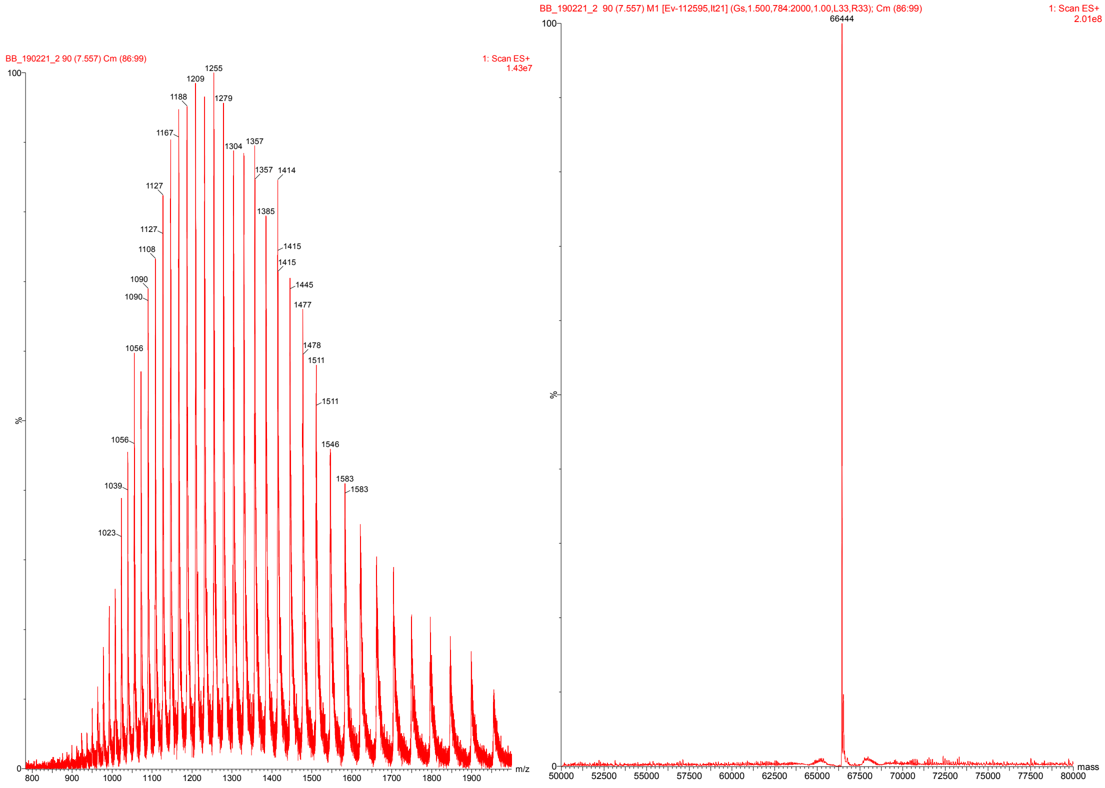

Figure S50. Combined ion series and deconvoluted mass spectra of the reaction of between rHSA-Cys34 (10 μM) with 1 equiv. of **1** after 1 h at 37 ºC (pH 5.5).

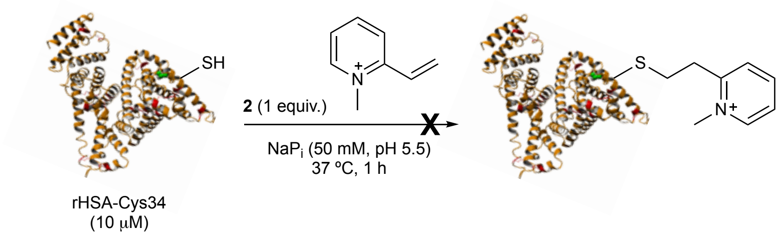

The reaction was performed according to the general procedure. To an eppendorf with 9.4 μL of NaPi (50 mM, pH 5.5) and 3 μL of DMF, was added a 27 μL aliquot of a stock solution of rHSA (15.05 μM) and the resulting mixture was vortexed for 10 seconds. Afterwards, a 0.83 mM solution of **2** (1 μL, 1 equiv.) in DMF was added and the reaction mixed for 1 h at 37 ºC. A 10 μL aliquot was analysed by LC-MS, and no conversion of the starting protein to a product was detected.

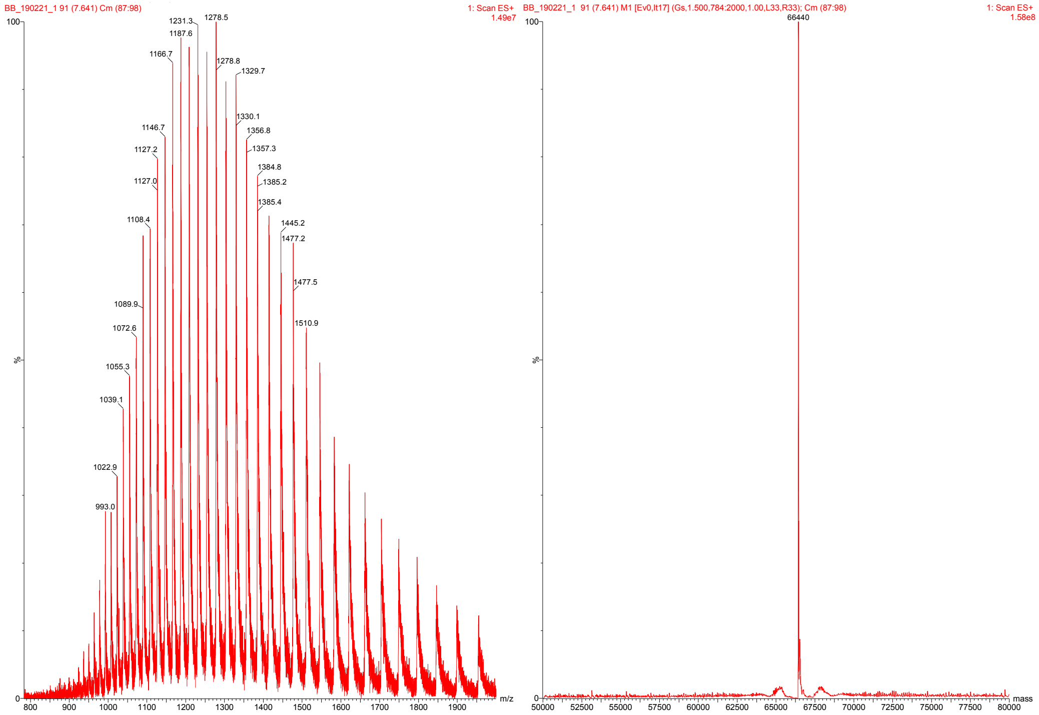

Figure S51. Combined ion series and deconvoluted mass spectra of the reaction of between rHSA-Cys34 (10 μM) with 1 equiv. of **2** after 1 h at 37 ºC (pH 5.5).

<u>Control:</u> Reaction of rHSA-**2** with 5,5′-dithiobis(2-nitrobenzoic acid)

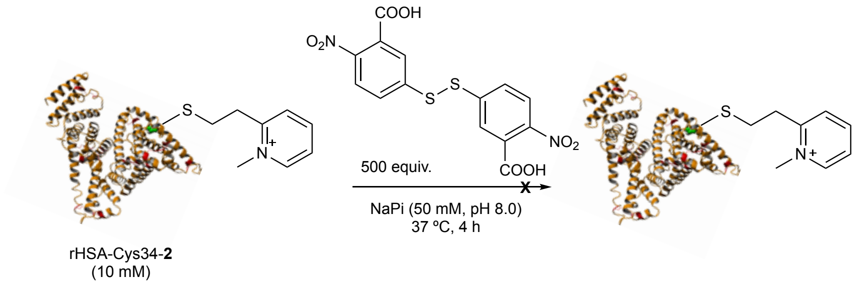

A 40 μL aliquot of rHSA-Cys34-**2** (10 μM) was transferred to a 0.5 mL eppendorf. An aliquot of 0.8 μL (500 equiv.) of a stock suspension of 5,5′-dithiobis(2-nitrobenzoic acid) (0.5 mM) was added and the resulting mixture vortexed for 10 seconds. After 4 h of additional mixing, at 37 ºC, small molecules were removed from the reaction mixture by loading the sample into a Zeba Spin Desalting Column previously equilibrated with NaP_i (50 mM, pH 8.0). The sample was eluted via centrifugation (2 min, 1000xg). A 10 μL aliquot was analysed by LC-MS and no conversion to the doubly modified protein was observed (calculated mass, 66754 Da; observed mass, 66558 Da).

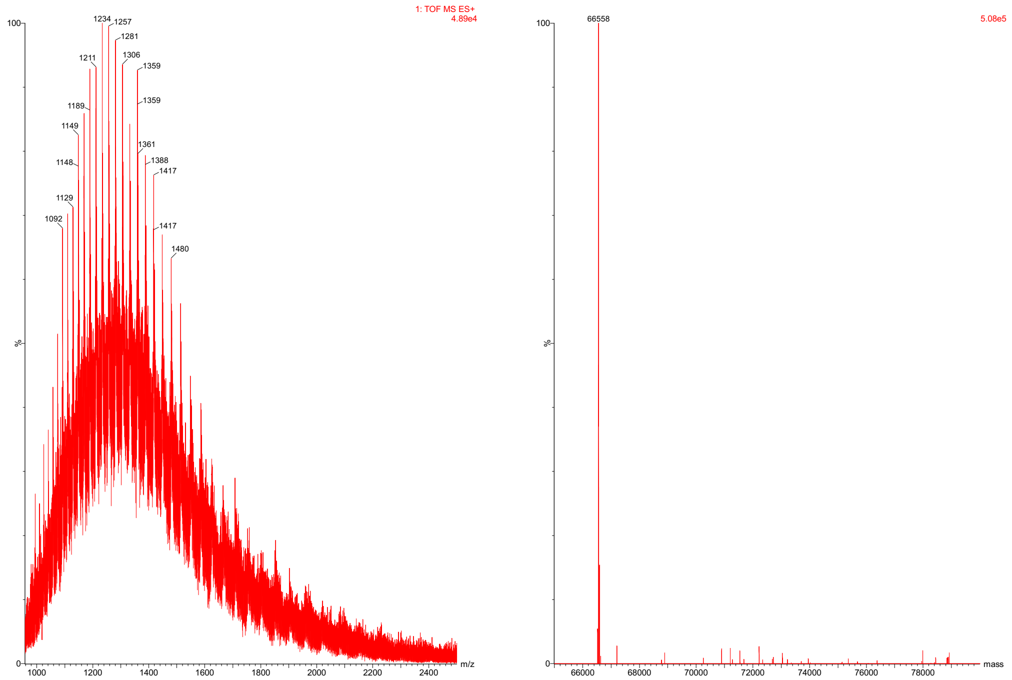

Figure S52. Combined ion series and deconvoluted mass spectra of the reaction of between rHSA-Cys34-**2** with 500 equiv. of 5,5′-dithiobis(2-nitrobenzoic acid) after 4 h at 37 ºC.

Control: Reaction of rHSA-Cys34-Ellman's with **2**

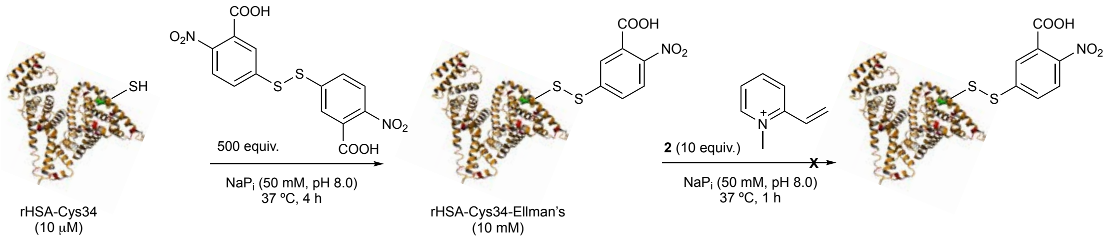

A 40 μL aliquot of rHSA-Cys34-Ellman's (10 μM) was transferred to a 0.5 mL eppendorf. An aliquot of 1 μL (10 equiv.) of a stock solution of **2** (8.3 mM) was added and the resulting mixture vortexed for 10 seconds. After 1 h of additional mixing, at 37 ºC, a 10 μL aliquot was analysed by LC-MS and no conversion to the doubly modified protein was observed (calculated mass, 66754 Da; observed mass, 66635 Da).

1st Step – Reaction of rHSA-Cys34 with with 5,5′-dithiobis(2-nitrobenzoic acid)

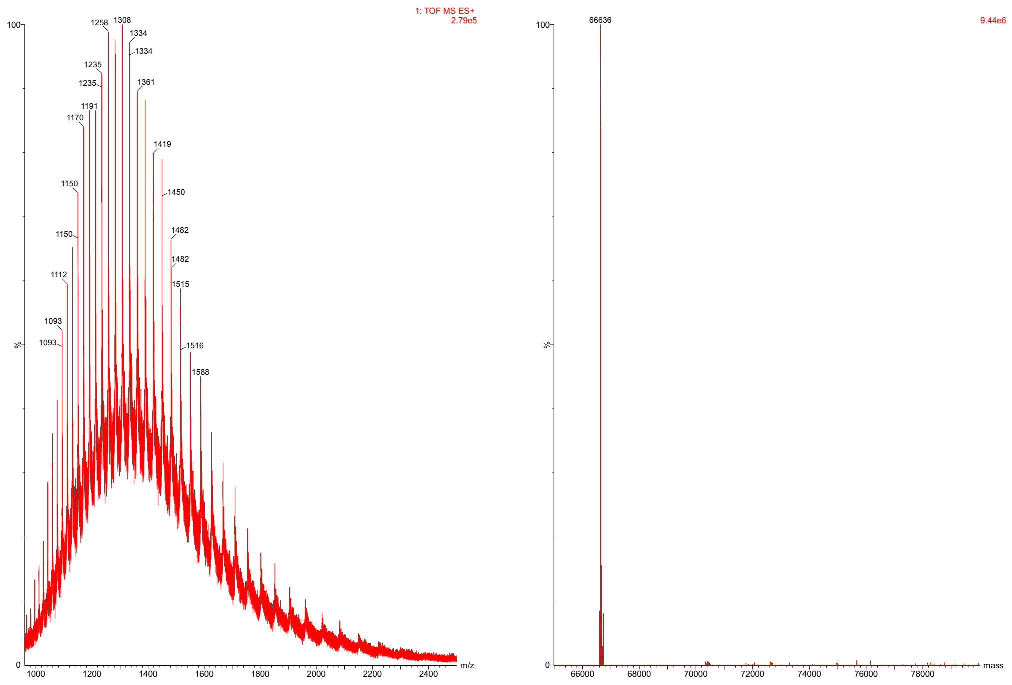

Figure S53. Combined ion series and deconvoluted mass spectra of the reaction of between rHSA-Cys34 with 500 equiv. of 5,5′-dithiobis(2-nitrobenzoic acid) after 4 h at 37 ºC.

2nd Step – Reaction of rHSA-Cys34-Ellman's with **2**

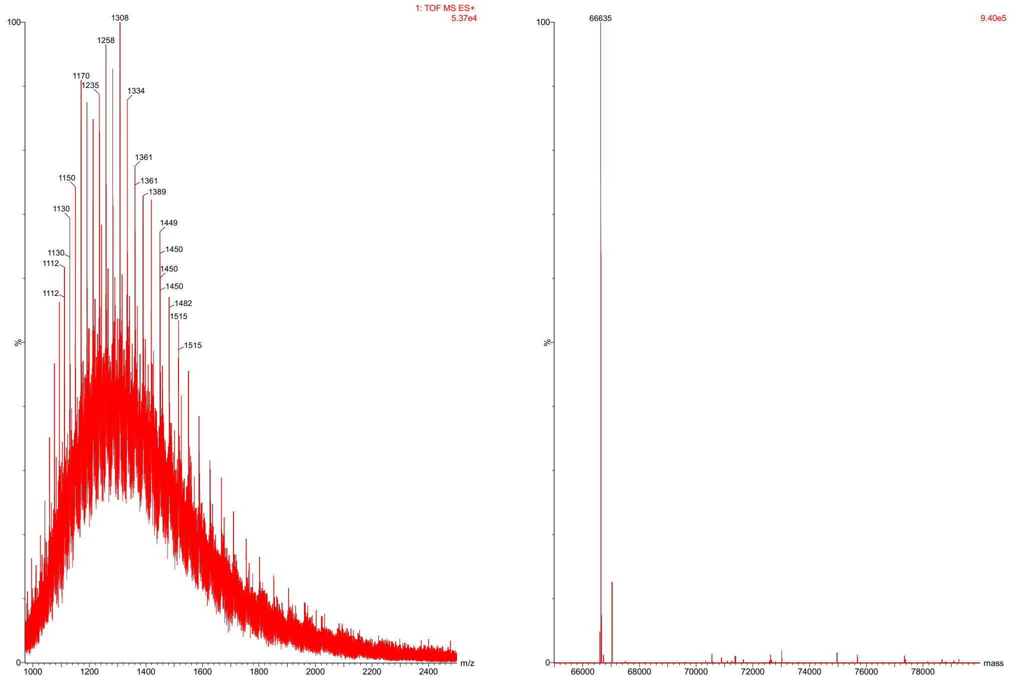

Figure S54. Combined ion series and deconvoluted mass spectra of the reaction of between rHSA-Cys34-Ellman's with 10 equiv. of **2** after 1 h at 37 ºC.

10. Thiomab-LC-C205C Modification and Characterization

Thiomab LC-V205C was kindly provided by Genentech Inc.

Reaction of Thiomab LC-V205C with **2**

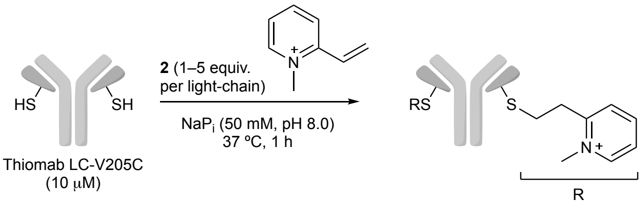

The reaction was performed according to the general procedure. To an eppendorf with 21 μL of NaP$_i$ (50 mM, pH 8.0) and 3.2 μL of DMF, was added a 15 μL aliquot of a stock solution of Thiomab LC-V205C (26.1 μM) and the resulting mixture was vortexed for 10 seconds. Afterwards, an 8.3 mM solution of **2** (1 μL, 10 equiv.) in DMF was added and the reaction mixed for 1 h at 37 ºC. At each reaction time, a 10 μL aliquot was analysed by LC-MS and conversion to a single modification per light-chain as well as two modifications per-light chain were observed (calculated mass for one modification, 23558 Da; calculated mass for two modifications, 23676 Da).

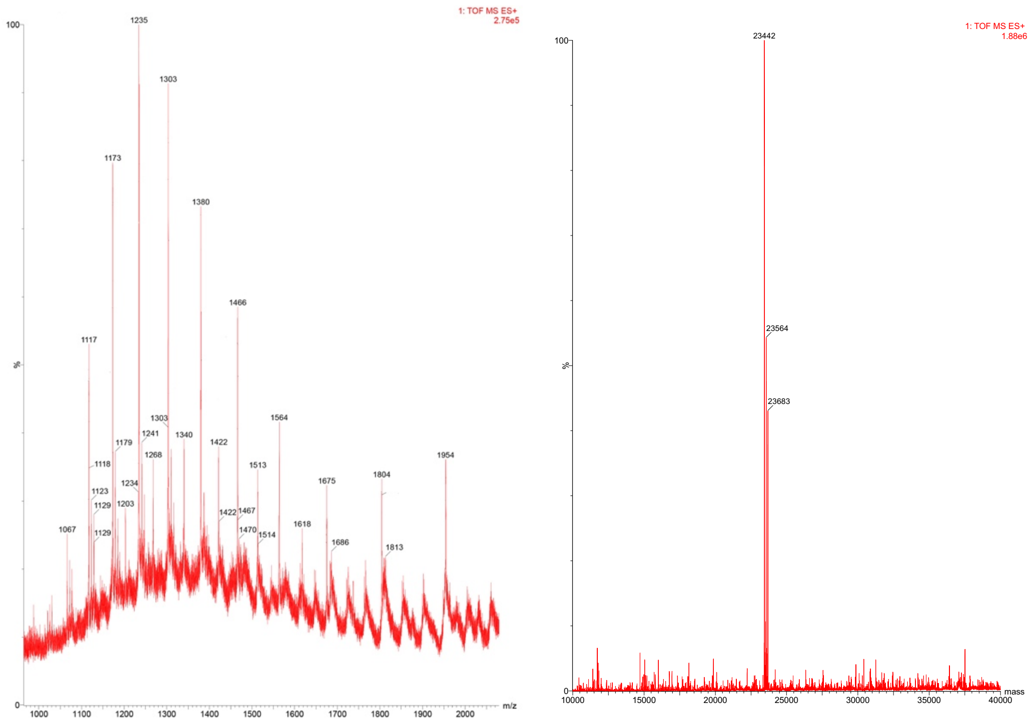

Figure S55. Combined ion series and deconvoluted mass spectra of the light-chain of Thiomab LC-V205C (10 μM) after reaction with **2** (1 equiv. per light-chain) for 1 h at 37 ºC.

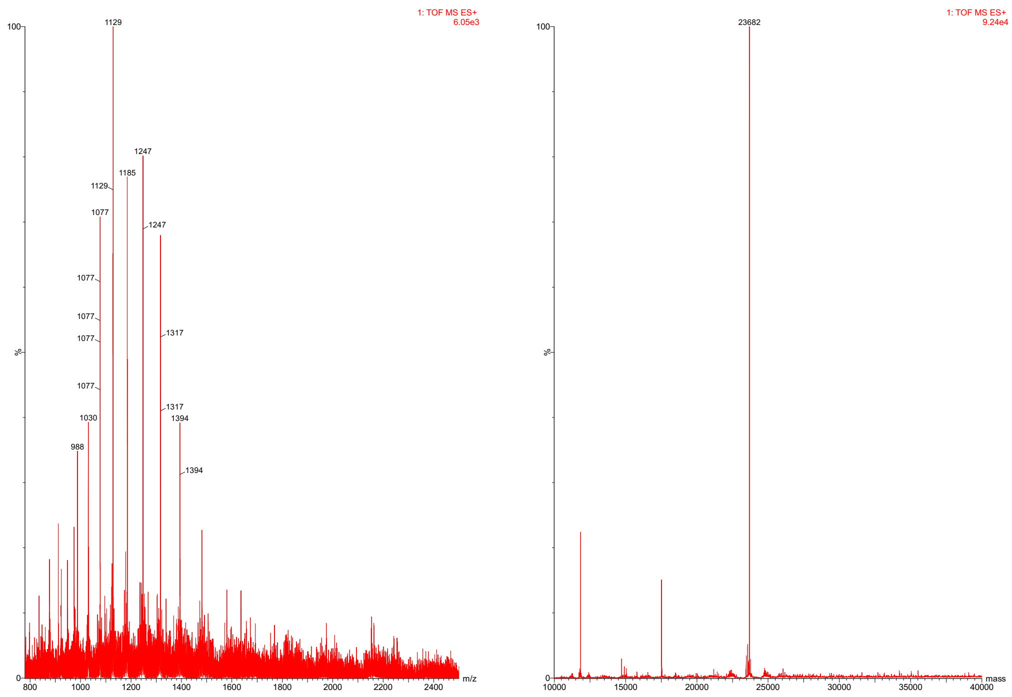

Figure S56. Combined ion series and deconvoluted mass spectra of the light-chain of Thiomab LC-V205C (10 μM) after reaction with **2** (5 equiv. per light-chain) for 1 h at 37 ºC.

Reaction of Thiomab LC-V205C with 5,5′-dithiobis(2-nitrobenzoic acid)

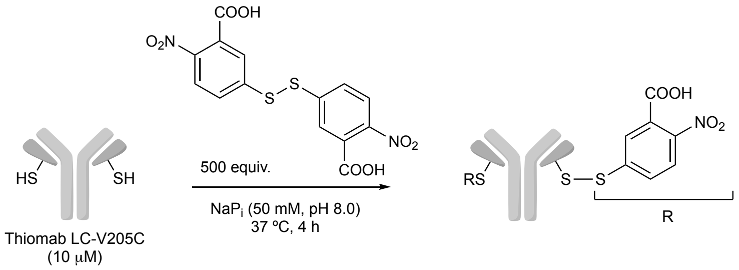

A 40 μL aliquot of Thiomab LC-V205C (10 μM) was transferred to a 0.5 mL eppendorf tube. An aliquot of 0.8 μL (500 equiv.) of a stock suspension of 5,5′-dithiobis(2-nitrobenzoic acid) (0.5 mM) was added and the resulting mixture vortexed for 10 seconds. After 4 h of additional mixing, at 37 ºC, small molecules were removed from the reaction mixture by loading the sample into a Zeba Spin Desalting Column previously equilibrated with NaP$_i$ (50 mM, pH 8.0). The sample was eluted via centrifugation (2 min, 1000xg). A 10 μL aliquot was analysed by LC-MS and conversion a conjugate bearing two modifications in the light-chain was observed (calculated mass for one modification in the light-chain, 23637 Da; calculated mass for two modifications in the light-chain, 23834 Da; observed mass, 23838 Da).

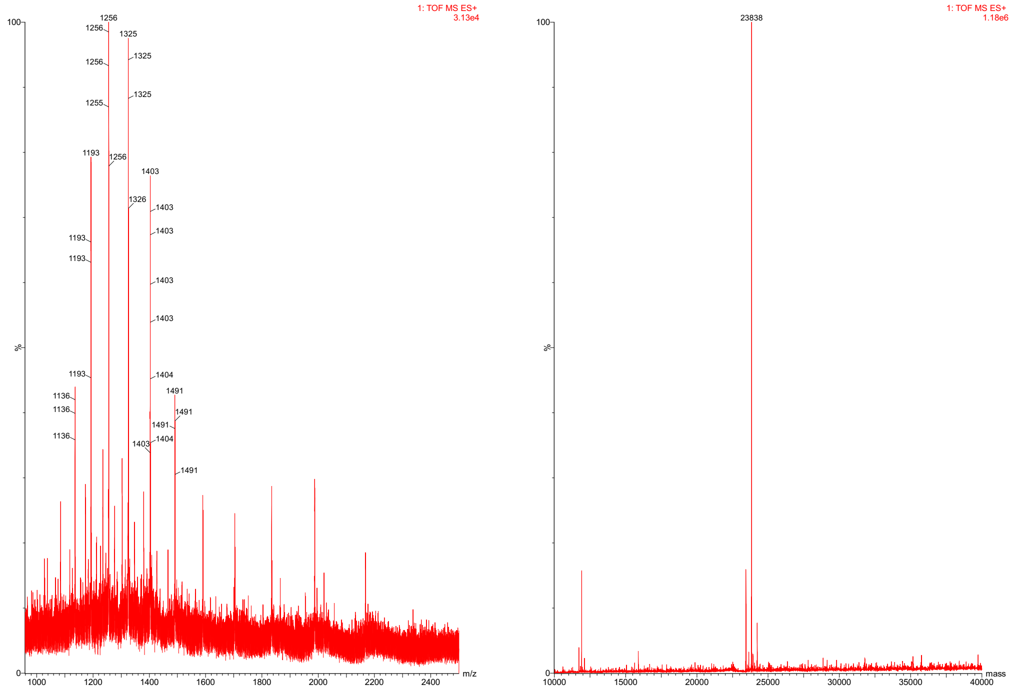

Figure S57. Combined ion series and deconvoluted mass spectra of the reaction between Thiomab LC-V205C and 5,5′-dithiobis(2-nitrobenzoic acid) (500 equiv.) after 4 h at 37 ºC.

Reaction of Thiomab LC-V205C with 1-(prop-2-yn-1-yl)-2-vinylpyridin-1-ium trifluoromethanesulfonate

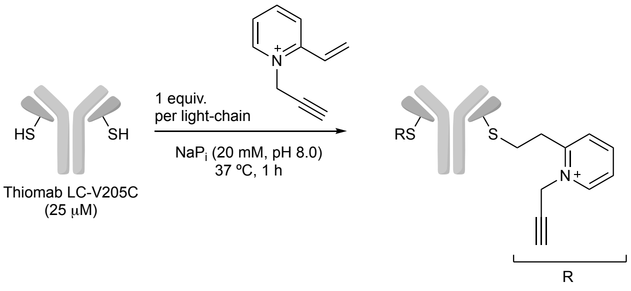

The reaction was performed according to the general procedure. To an eppendorf with 24 μL of NaP$_i$ (20 mM, pH 8.0) and 3.8 μL of DMF, was added a 12.5 μL aliquot of a stock solution of Thiomab LC-V205C (80 μM) and the resulting mixture was vortexed for 10 seconds. Afterwards, a 12.0 mM solution of 1-(prop-2-yn-1-yl)-2-vinylpyridin-1-ium trifluoromethanesulfonate (0.16 μL, 1 equiv. per light-chain) in DMF was added and the reaction mixed for 1 h at 37 ºC. At each reaction time, a 10 μL aliquot was analysed by LC-MS and conversion to the expected product was observed (calculated mass for the light-chain, 23583 Da; observed mass for the light-chain, 23601 Da).

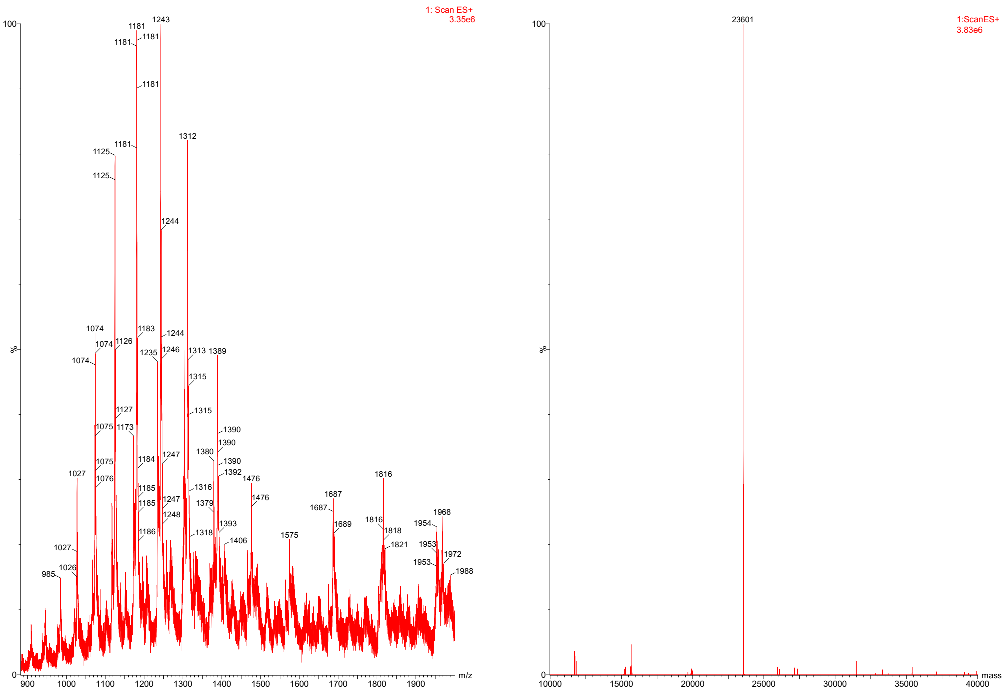

Figure S58. Combined ion series and deconvoluted mass spectra of the light- chain of Thiomab LC-V205C (25 μM) after reaction with 1-(prop-2-yn-1-yl)-2-vinylpyridin-1-ium trifluoromethanesulfonate (1 equiv. per light-chain) for 1 h at 37 ºC.

Reaction of Thiomab LC-V205C with **5**

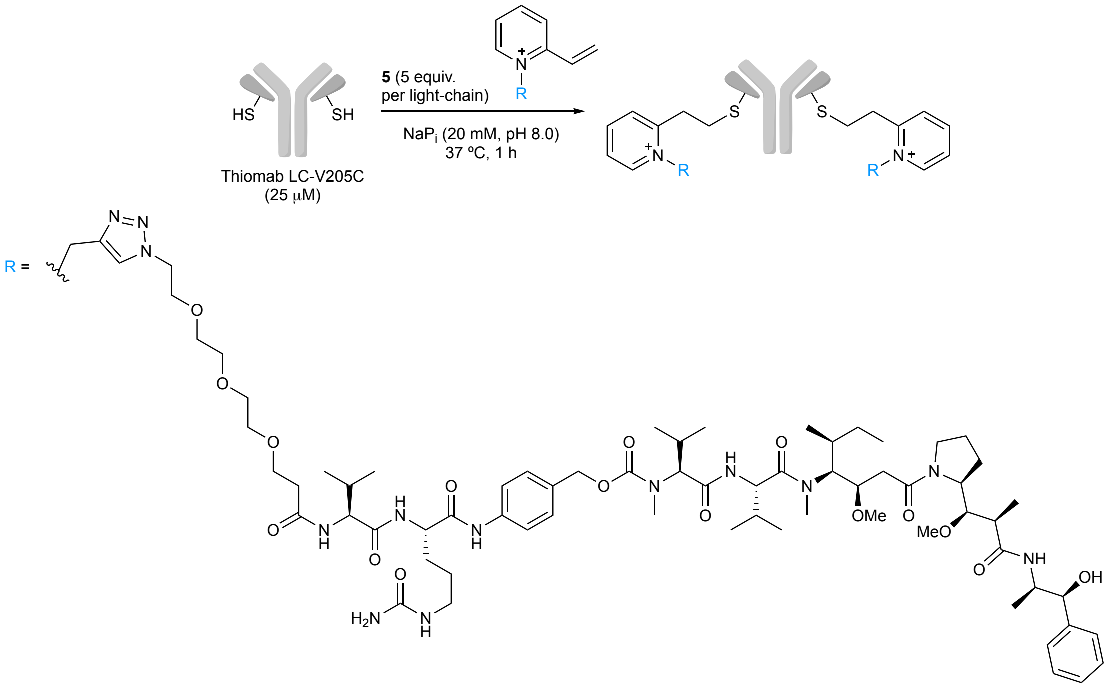

The reaction was performed according to the general procedure. To an eppendorf with 24 µL of NaP_i (20 mM, pH 8.0) and 3.8 µL of DMF, was added a 12.5 µL aliquot of a stock solution of Thiomab LC-V205C (80 µM) and the resulting mixture was vortexed for 10 seconds. Afterwards, a 1.86 mM solution of **5** (5.36 µL, 10 equiv.) in DMF was added and the reaction mixed for 1 h at 37 ºC. At each reaction time, a 10 µL aliquot was analysed by LC-MS and conversion to the expected product was observed (calculated mass for the light-chain, 24935 Da; observed mass for the light-chain, 24935 Da; calculated mass for the heavy-chain, 50595 Da; observed mass for the heavy-chain, 50590 Da).

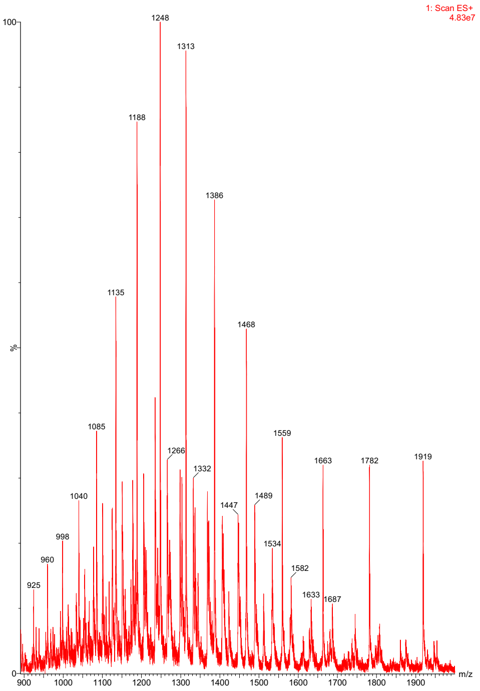

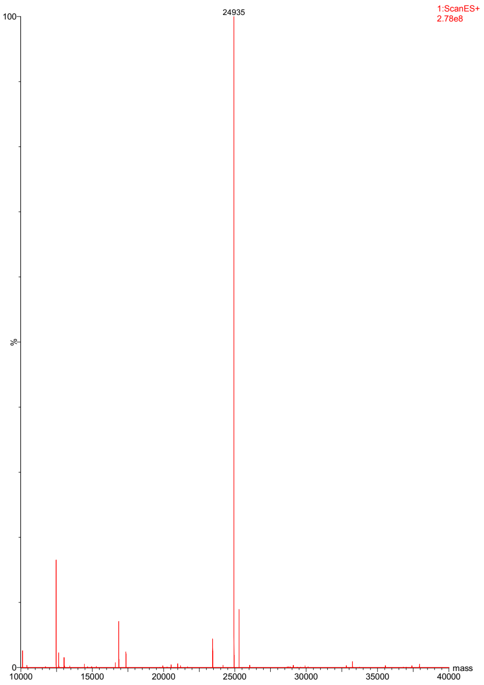

Figure S59. Combined ion series and deconvoluted mass spectra of the light-chain of Thiomab LC-V205C (25 μM) after reaction with **5** (5 equiv. per light chain) for 1 h at 37 ºC.

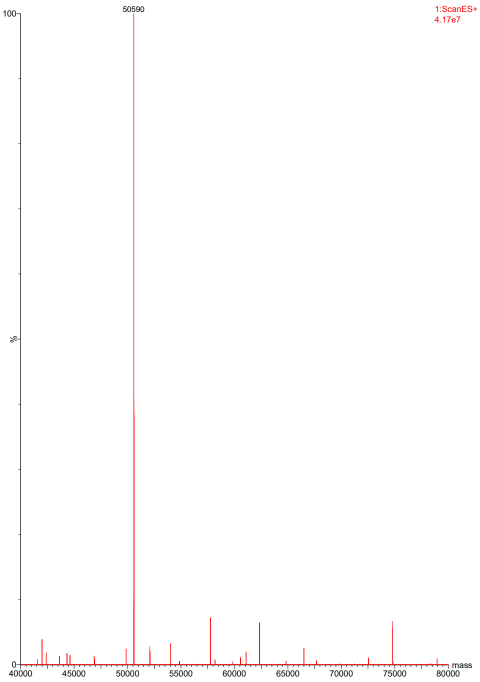

Figure S60. Combined ion series and deconvoluted mass spectra of the heavy-chain of Thiomab LC-V205C (25 μM) after reaction with **5** (5 equiv. per light-chain) for 12 h at 37 ºC.

11. Analysis of Secondary Structural Content by CD

Circular dichroism (CD) spectroscopy was used to analyse protein secondary structure in solution. Samples were concentrated to 100 nM in NaP_i buffer (50 mM, pH 8.0). CD measurements were made using Applied Photophysics' Chirascan CD spectrometer equipped with a Quantum TC125 temperature control unit 25 ºC. The data was acquired in a 1 mm cuvette path length with a response time of 1 s, a per-point acquisition delay of 5 ms and a pre and post-scan delay of 50 ms. Spectra were averaged over three scans, in a wavelength range from 190 nm to 260 nm, and the spectrum from a blank sample containing only buffer was subtracted from the averaged data.

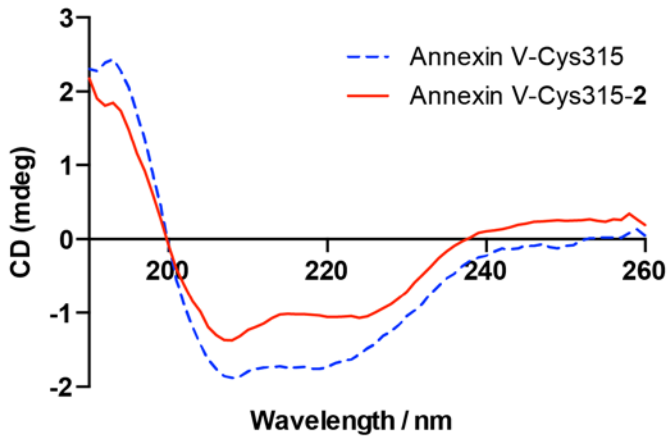

Figure S61. CD spectrum for Annexin V-Cys315 and Annexin V-Cys3515-**2**.

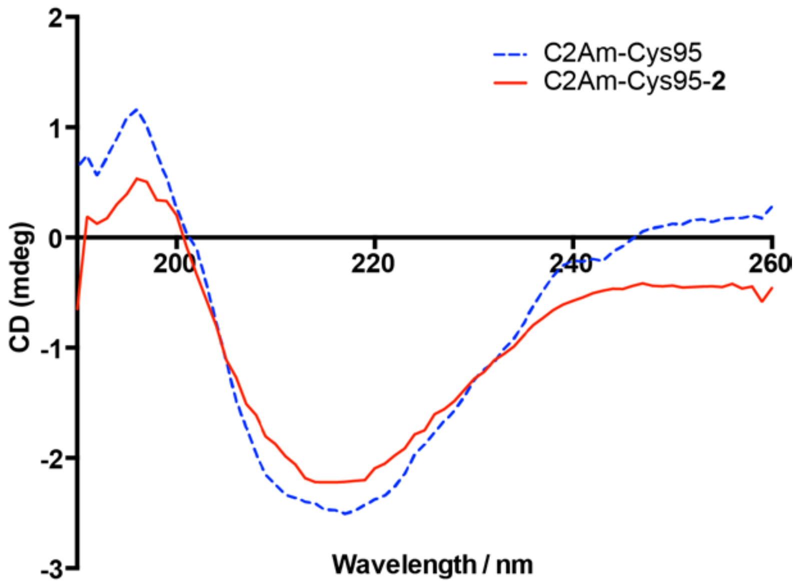

Figure S62. CD spectrum for C2Am-Cys95 and C2Am-Cys95-**2**.

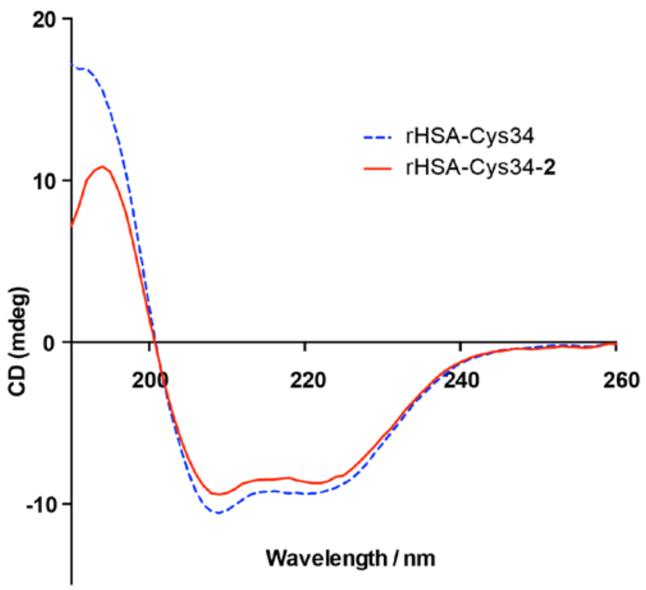

Figure S63. CD spectrum for rHSA-Cys34 and rHSA-Cys34-**2**.

12. Determination of FcRn Binding by Surface Plasmon Resonance (SPR)

A BIAcore 3000 instrument (GE Healthcare) was used with CM5 sensor chips coupled with mFcRn (cynomolgus monkey FcRn) or hFcRn (1,000 resonance units) using amine coupling chemistry as described by the manufacturer. The coupling was performed by injecting 5-10 µg/mL of each protein into 10 mm sodium acetate, pH 5.0 (GE Healthcare). For all experiments, phosphate buffer (67 mM phosphate buffer, 0.15 M NaCl, 0.005% Tween 20) with pH 5.5, 6.0, or 7.4 was used as running buffer and dilution buffer. Regeneration of the surfaces were achieved using injections of HBS-EP buffer (0.01 M HEPES, 0.15 M NaCl, 3 mM EDTA, 0.005% surfactant P20) at pH 7.4 (GE Healthcare).

Albumins were 8-step serially diluted 1:1 (10 µM – 0.156 µM + 0 µM) down a microtitre plate in running buffer and flowed over immobilised shFcRn to obtain kinetic data and confirm KD values. Flow rate was 30 µL/min and injection time was 60 seconds with a 60 seconds' delay before washing. All injections required a 12 seconds' regeneration pulse (HBS-EP) post injection to restore the baseline. Kinetic parameters were extrapolated using BIAevaluation 1:1 Langmuir model (BIAcore AB). Data was reference cell adjusted and zero-adjusted.

Table S2. Assessment of the FcRn binding properties of rHSA-Cys34-**2**. Biacore SPR assessment of human FcRn binding of albumins at 10 µM.

Albumin Variant	k_{on} (x10^3/Ms)	k_{off} (x10^{-3}/Ms)	k_D (µM)
rHSA-Cys34	5.28	62.1	11.8
rHSA-Cys34-**2**	2.16	19.1	8.88

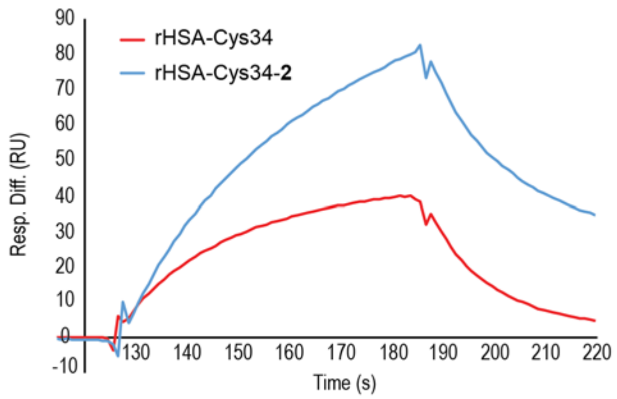

Figure S64. Assessment of the FcRn binding properties of rHSA-Cys34-**2**. Biacore SPR assessment of human FcRn binding of albumins at 10 µM.

13. Cell Specificity Analysis by Flow Cytometry

Cell culture.

SKBR3 cells (human breast adenocarcinoma cell line) were used for the *in vitro* studies. The cells were maintained in a humidified incubator at 37 ºC under 5% CO_2 and grown using 1x D-MEM (Dulbecco's modified Eagle medium) with Sodium Pyruvate and without L-Glutamine (Invitrogen, Life Technologies) supplemented with 10% heat-inactivated fetal bovine serum (FBS) (Gibco, Life Technologies), 1x MEM NEAA (Gibco, Life Technologies) ,1x GlutaMAX (Gibco, Life Technologies), 200 units/mL penicillin and 200 µg/mL streptomycin (Gibco, Life Technologies) and 10 mM HEPES (Gibco, Life Technologies).

Thiomab LC205C-4 specificity as determined by flow cytometry analysis.

The specificity of the antibody Thiomab LC205C-**5** was determined by flow cytometry analysis. For this purpose, SKRB3 cells (with high expression of HER2 receptor) were plated in 96well round bottom plates (100.000 cells per well) and blocked for 1h with 10% FBS in 1x PBS (flow cytometry buffer). After this blocking step, the cells were incubated with 30µL of 50nM MJMP265 at room temperature. After 1 h of incubation cells were washed three times (100µL flow cytometry buffer added and centrifuged for 5 min at 400G) and incubated with 30 µL/well of Goat anti-Human IgG (H+L) Cross-Adsorbed Secondary Antibody, Alexa Fluor 647 (cat. No A21445, Life Technolonies) at 10 µg/mL, for 1 h. After this incubation period, the cells were washed one time as previously described, re-suspended in 400 µL of 10%FBS in PBS and transferred to flow cytometry tubes. Acquisition was done using a BD LSR Fortessa set up with a 640 nm laser and a 670/14 nm band-pass filter (combination used for APC detection). Data analysis was done with FlowJo (version 6.3.4, FlowJo) software. Data represents mean ± s.d of 3 biological replicates and only single-cell events are shown.

14. Microfluidic Determination of Electrophoretic Mobility

Device fabrication.

The electrophoresis device was fabricated using standard soft lithography techniques[11] of poly(dimethylsiloxane) (PDMS, Dow Corning) using SU-8 on silicon wafer master. To decrease the autofluorescence of the PDMS carbon nanopowder (13 nm, Plasmachem GMBH) was added to the PDMS mixture prior to curing. In addition, the channels were sealed with quartz microscope glass (Alfa Aesar, 76.2x25.4x1.0mm). This was done by plasma treated both PDMS and quartz surfaces with oxygen plasma for 30 s (Electronic Diener Femto, 40% power for 30 seconds) and heating at 95 ºC for a 1 min. An additional hydrophobic treatment was performed right before the experiments with oxygen plasma (500 s with 80 % power).

Microfluidic electrophoresis experiments.

The experiments were conducted with microfluidic free flow electrophoresis device[12] withdrawing at 400 $\frac{\mu l}{h}$ rate from the fluid outlets while pushing the electrode solution at 65 $\frac{\mu l}{h}$ from the electrode inlets. Buffer and the analyte sample were loaded to the device using gel loading chips. The voltages (0–120 V) were applied between the electrodes by connecting a voltage source to the metal pins in the electrode outlet. The deflection (σ) of the sample while increasing the voltage was observed with a UV microscope (ex. 280, em. 350 nm) and a CCD camera. The electric mobility (μ_{el}) of the sample was calculated from the equation:

$$\mu_{el} = \frac{v_{drift}}{E} \tag{1}$$

where drift velocity can be estimated from:

$$v_{drift} = \frac{\sigma}{t_{res}} \tag{2}$$

and an electric field is simply from the measured voltage in the channel and device diameter:

$$E = \frac{V}{d} \tag{3}$$

The voltage in the channel was calculated by measuring the voltage efficiency of each device. This was done by comparing the measured current of the buffer and highly conductive reference solution (3 M KCl). Also, the average residence time of the fluids in the chamber was estimated:

$$t_{res} = \frac{w * L * h}{v} = \frac{2mm * 50\mu m * 3.6mm}{269\,\frac{\mu l}{h}} = 4.8\ s$$

where w, L and h are the width, length and height. Further from the mobility the charges (q) can be estimated using:

$$q = \frac{\mu_{el}}{D * kT}$$

15. NMR Spectra

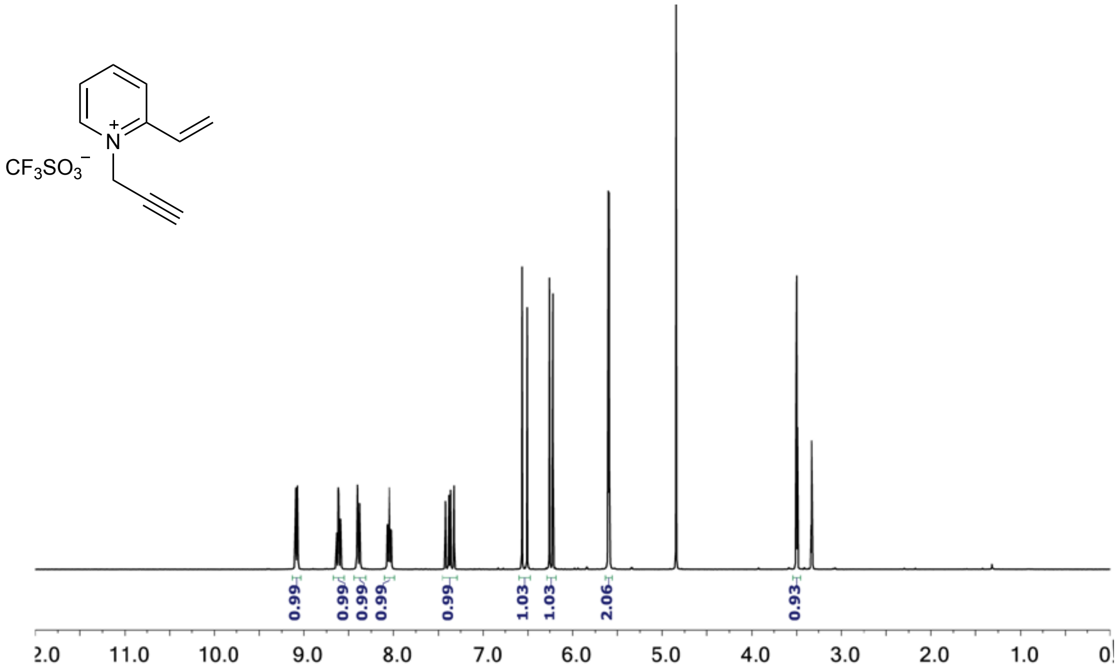

Figure S65. ^{1}H NMR (300 MHz, CD_3OD) of 1-(prop-2-yn-1-yl)-2-vinylpyridin-1-ium trifluoromethanesulfonate recorded at 298K.

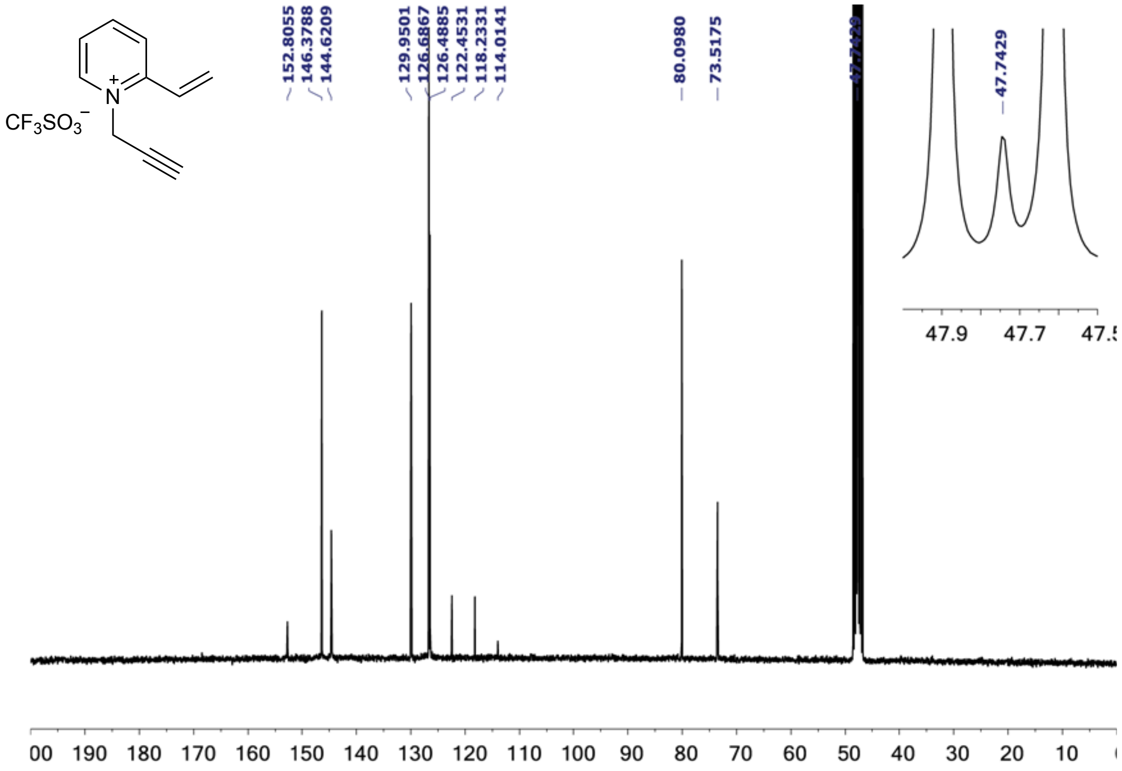

Figure S66. ^{13}C NMR (75 MHz, CD_3OD) of 1-(prop-2-yn-1-yl)-2-vinylpyridin-1-ium trifluoromethanesulfonate recorded at 278 K.

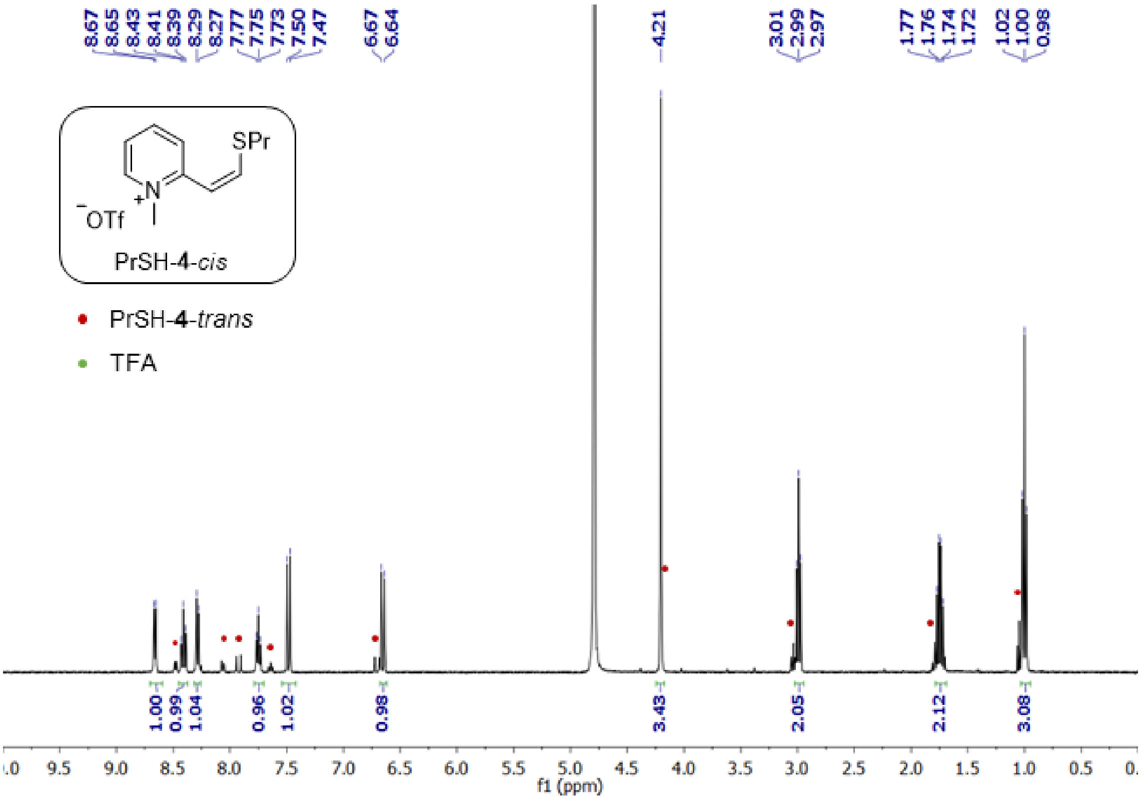

Figure S67. ^{1}H NMR (400 MHz, D_2O) of compound PrSH-**4**-*cis* recorded at 298 K.

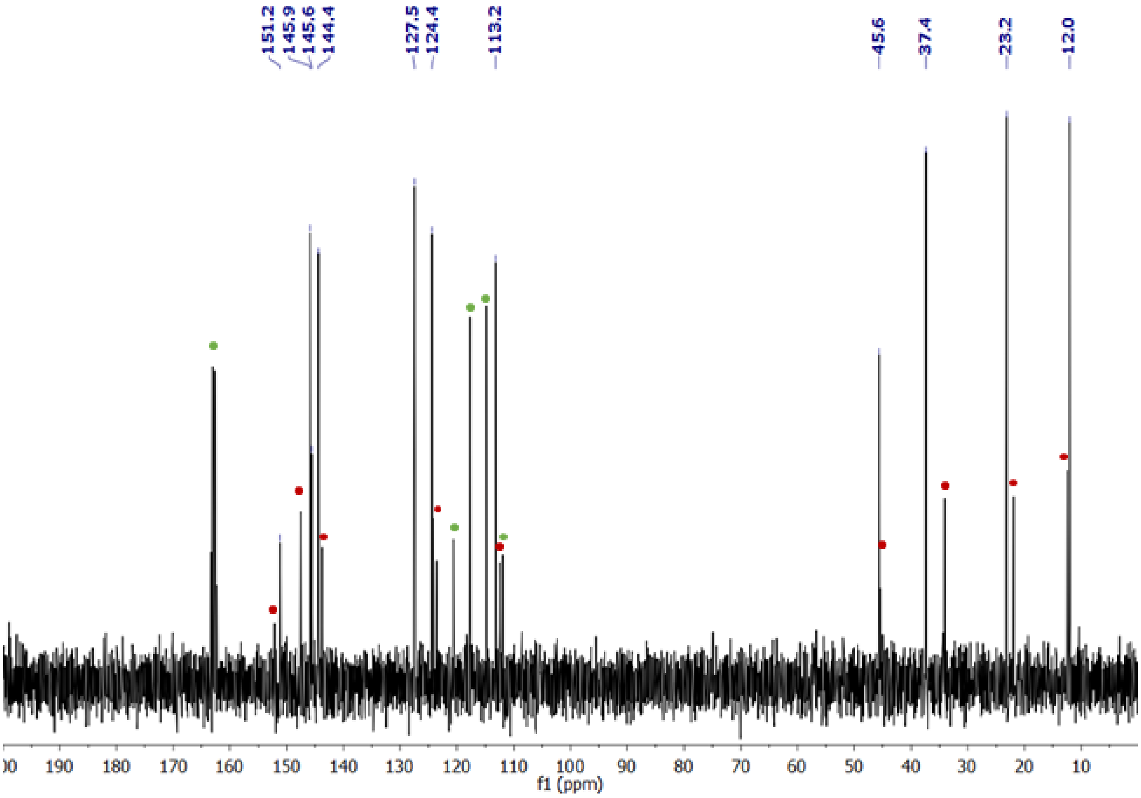

Figure S68. ^{13}C NMR (100 MHz, D_2O) of compound PrSH-**4**-*cis* recorded at 298 K.

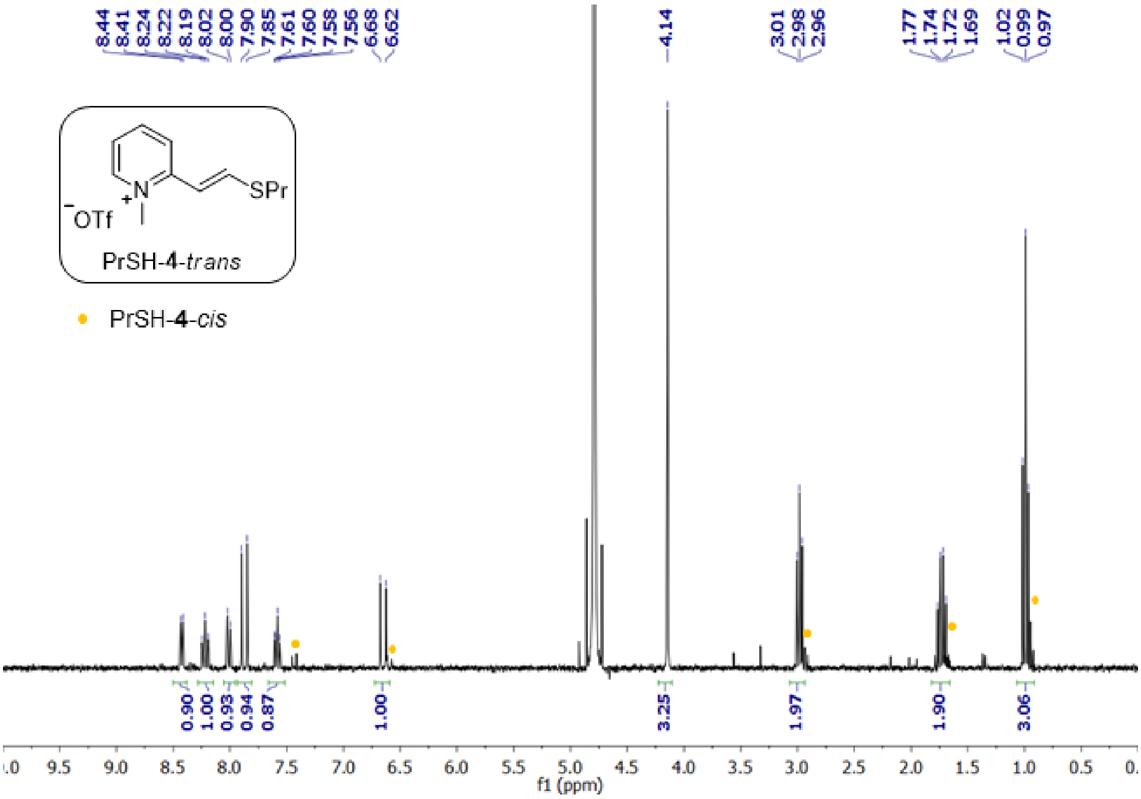

Figure S69. ^{1}H NMR (400 MHz, D_2O) of compound PrSH-**4**-*trans* recorded at 298 K.

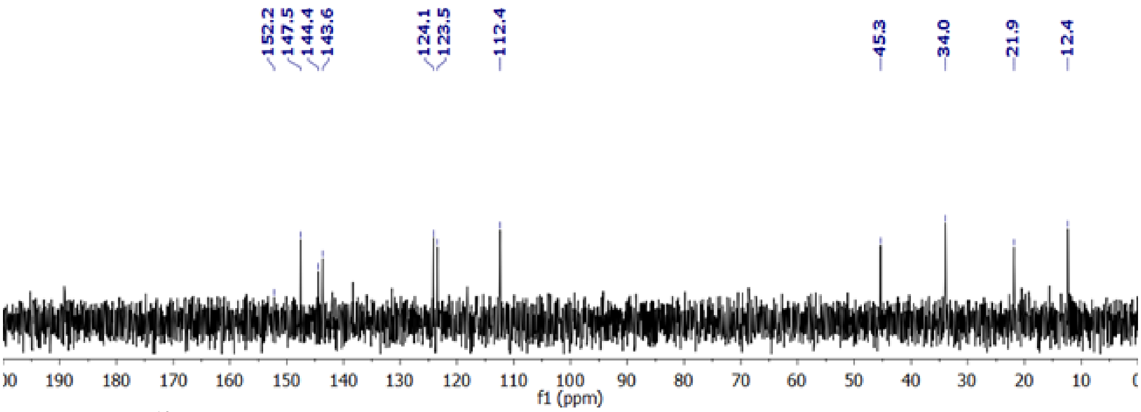

Figure S70. ^{13}C NMR (100 MHz, D_2O) of compound PrSH-**4**-*trans* recorded at 298 K.

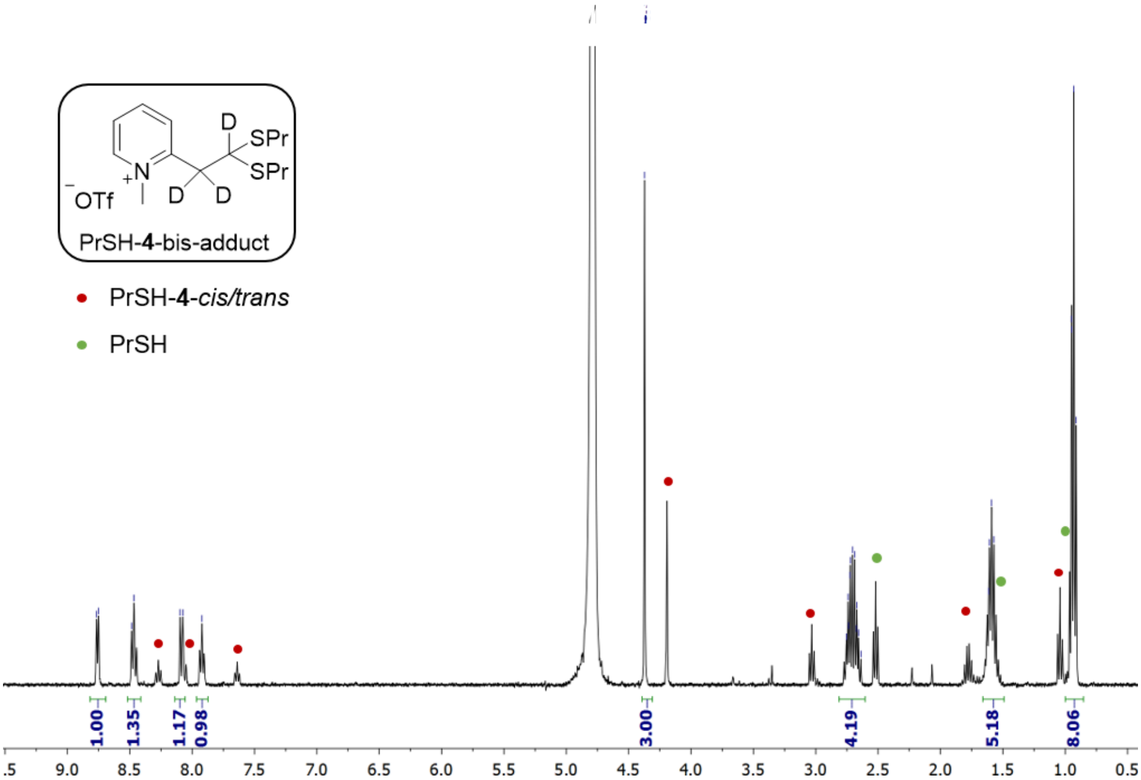

Figure S71. ^{1}H NMR (400 MHz, D_2O) of compound PrSH-**4**-bis-adduct recorded at 298 K.

16. References

[1] M. J. Frisch, G. W. Trucks, H. B. Schlegel, G. E. Scuseria, M. A. Robb, J. R. Cheeseman, G. Scalmani, V. Barone, G. A. Petersson, H. Nakatsuji, X. Li, M. Caricato, A. V. Marenich, J. Bloino, B. G. Janesko, R. Gomperts, B. Mennucci, H. P. Hratchian, J. V. Ortiz, A. F. Izmaylov, J. L. Sonnenberg, Williams, F. Ding, F. Lipparini, F. Egidi, J. Goings, B. Peng, A. Petrone, T. Henderson, D. Ranasinghe, V. G. Zakrzewski, J. Gao, N. Rega, G. Zheng, W. Liang, M. Hada, M. Ehara, K. Toyota, R. Fukuda, J. Hasegawa, M. Ishida, T. Nakajima, Y. Honda, O. Kitao, H. Nakai, T. Vreven, K. Throssell, J. A. Montgomery Jr., J. E. Peralta, F. Ogliaro, M. J. Bearpark, J. J. Heyd, E. N. Brothers, K. N. Kudin, V. N. Staroverov, T. A. Keith, R. Kobayashi, J. Normand, K. Raghavachari, A. P. Rendell, J. C. Burant, S. S. Iyengar, J. Tomasi, M. Cossi, J. M. Millam, M. Klene, C. Adamo, R. Cammi, J. W. Ochterski, R. L. Martin, K. Morokuma, O. Farkas, J. B. Foresman, D. J. Fox, Wallingford, CT, **2016**.

[2] Y. Zhao, D. G. Truhlar, *Theor. Chem. Acc.* **2008**, *120*, 215–241.

[3] G. Scalmani, M. J. Frisch, *J. Chem. Phys.* **2010**, *132*, 114110.

[4] R. F. Ribeiro, A. V. Marenich, C. J. Cramer, D. G. Truhlar, *J. Phys. Chem. B.* **2011**, *115*, 14556–14562.

[5] a) C. Gonzalez, H. B. Schlegel, *J. Chem. Phys.* **1989**, *90*, 2154–2161; b) C. Gonzalez, H. B. Schlegel, *J. Phys. Chem.* **1990**, *94*, 5523–5527.

[6] A. Nuñez, B. Abarca, A. M. Cuadro, J. Alvarez-Builla, J. J. Vaquero, *J. Org. Chem.* **2009**, *74*, 4166–4176.

[7] S. O. Doronina, B. E. Toki, M. Y. Torgov, B. A. Mendelsohn, C. G. Cerveny, D. F. Chace, R. L. DeBlanc, R. P. Gearing, T. D. Bovee, C. B. Siegall, J. A. Francisco, A. F. Wahl, D. L. Meyer, P. D. Senter, *Nat. Biotechnol.* **2003**, *21*, 778.

[8] B. Lee, S. Sun, E. Jiménez-Moreno, A. A. Neves, G. J. L. Bernardes, *Bioorg. Med. Chem.* **2018**, *26*, 3060–3064.

[9] A. M. Freedy, M. J. Matos, O. Boutureira, F. Corzana, A. Guerreiro, P. Akkapeddi, V. J. Somovilla, T. Rodrigues, K. Nicholls, B. Xie, G. Jiménez-Osés, K. M. Brindle, A. A. Neves, G. J. L. Bernardes, *J. Am. Chem. Soc.* **2017**, *139*, 18365–18375.

[10] I. S. Alam, A. A. Neves, T. H. Witney, J. Boren, K. M. Brindle, *Bioconjug. Chem.* **2010**, *21*, 884–891.

[11] D. C. Duffy, J. C. McDonald, O. J. A. Schueller, G. M. Whitesides, *Anal. Chem.* **1998**, *70*, 4974–4984.

[12] K. L. Saar, Y. Zhang, T. Müller, C. P. Kumar, S. Devenish, A. Lynn, U. Łapińska, X. Yang, S. Linse, T. P. J. Knowles, *Lab Chip* **2018**, *18*, 162–170.
